# Comparing the cost-per-QALYs gained and cost-per-DALYs averted literatures

**DOI:** 10.12688/gatesopenres.12786.2

**Published:** 2018-03-05

**Authors:** Peter J. Neumann, Jordan E. Anderson, Ari D. Panzer, Elle F. Pope, Brittany N. D'Cruz, David D. Kim, Joshua T. Cohen

**Affiliations:** 1 Center for the Evaluation of Value and Risk in Health, Institute for Clinical Research and Health Policy Studies, Tufts Medical Center, Boston, MA, USA

**Keywords:** Quality-adjusted life years, Disability-adjusted life years, Cost-effectiveness

## Abstract

**Background**: We examined the similarities and differences between studies using two common metrics used in cost-effectiveness analyses (CEAs): cost per quality-adjusted life year (QALY) gained and cost per disability-adjusted life year (DALY) averted.

**Methods**: We used the Tufts Medical Center CEA Registry, which contains English-language cost-per-QALY gained studies, and the Global Cost-Effectiveness Analysis (GHCEA) Registry, which contains cost-per-DALY averted studies. We examined study characteristics, including intervention type, sponsor, country, and primary disease, and also compared the number of published CEAs to disease burden for major diseases and conditions across geographic regions.

**Results**: We identified 6,438 cost-per-QALY and 543 cost-per-DALY studies published through 2016 and observed rapid growth for both literatures. Cost-per-QALY studies most often examined pharmaceuticals and interventions in high-income countries. Cost-per-DALY studies predominantly focused on infectious disease interventions and interventions in low and lower-middle income countries. We found that while diseases imposing a larger burden tend to receive more attention in the cost-effectiveness analysis literature, the number of publications for some diseases and conditions deviates from this pattern, suggesting “under-studied” conditions (e.g., neonatal disorders) and “over-studied” conditions (e.g., HIV and TB).

**Conclusions**: The CEA literature has grown rapidly, with applications to diverse interventions and diseases.  The publication of fewer studies than expected for some diseases given their imposed burden suggests funding opportunities for future cost-effectiveness research.

## Introduction

Researchers conducting cost-effectiveness analyses (CEAs) commonly use quality-adjusted life years (QALYs) or disability-adjusted life years (DALYs) as health outcome measures to account for both longevity and quality of life (or life with disability) ^1^. These broadly applicable metrics facilitate the comparison of interventions across conditions and diseases.

Analysts have used these measures in different contexts and settings ^2– 6^. CEAs using the cost-per-QALY metric, which first appeared in the late 1970s, have typically focused on interventions in higher income settings ^7, 8^. In the 1990s, the World Bank and the World Health Organization (WHO) developed the DALY to quantify disease burden (reflecting both years of life lost (YLL) and years of life with disability (YLD)) ^9, 10^. CEAs using DALYs have tended to focus on lower- and middle-income countries ^11^.

QALYs and DALYs, which both quantify health related quality of life by assigning a value ranging from zero to one to each year of life, have somewhat different methodological underpinnings ^12^. QALY preference weights range from 0 (corresponding to “dead”) to 1 (corresponding to a hypothetical state of “perfect health”) and reflect a set of health state “attributes,” “dimensions,” or “domains” – e.g., discomfort, mobility, depression, etc. – associated with an individual’s health condition. DALY weights have a similar intuitive interpretation, although for DALYs, 1 corresponds to “dead” and 0 corresponds to “perfect health.” For DALYs, moreover, each weight corresponds not to a set of health state attributes but to a specific disease ^13^.

DALY values have in the past depended on the age of the affected populations. “Age-weighting” reflected the idea that an additional life year accrued during childhood or old age has less value than a year accrued during young and middle adulthood, when productivity contributions to societal well-being are typically greatest ^14, 15^. Because the unequal treatment of different age groups raised substantial ethical concerns, however, the most recent DALY calculation methods omit age-weighting ^16^.

We analyzed the cost-per-QALY gained and cost-per-DALY averted literatures to examine their growth and regional variation, and to investigate the extent to which the focus of each literature corresponds to those diseases and conditions imposing the largest burden on the population.

## Methods

### Data

***The cost-effectiveness analysis literature*.** We analyzed two databases maintained by the Center for the Evaluation of Value and Risk in Health at Tufts Medical Center in Boston, Massachusetts: the Cost-Effectiveness Analysis (CEA) Registry ( www.cearegistry.org), which contains information on cost-per-QALY studies, and the Global Health CEA Registry ( www.ghcearegistry.org), which houses information on cost-per-DALY studies. Both registries contain information on PubMed-indexed, English-language CEAs published through 2016. Previous publications further detail the search strategies, data collection processes, and review methods, which are similar for the two registries ^5, 6^. We received an ethics exemption for this study because it did not involve human subjects. Data from these registries used in this analysis appear in Dataset 1 and Dataset 2; Supplemental File 1 and Supplemental File 2 document the variables in these datasets.

***Disease burden*.** Dataset 3 contains population disease burden estimates (total DALYs incurred), as reported by the Institute for Health Metrics and Evaluation (IHME), and stratified by Global Burden of Disease (GBD) Super Region ^17^. Within each Super Region, we sub-stratified population burden by GBD level two disease category. Dataset 3 also lists the number of articles from the cost-per-QALY literature and from the cost-per-DALY literature for each of these strata and substrata. We counted articles in more than one of the Table 2 strata if, for example, they focused on two countries belonging to two distinct GBD Super Regions.

### Analysis

***Study characteristics*.** Using data from Dataset 1 and Dataset 2, and definitions from the World Bank and the GBD initiative, we stratified studies by: GBD Super Region, World Bank income level, intervention type, study funding source category, prevention stage, and GBD category. As detailed in Table 1, some of these categories are mutually exclusive, while others are not. We computed the proportion of studies in each stratum using total article counts for the cost-per-QALY and cost-per-DALY literature from Dataset 1 and Dataset 2, respectively.

**Table 1.** Characteristics of published CEAs using cost-per-QALY and cost-per-DALY through 2016.

	Cost-per-QALY studies	Cost-per-DALY studies	Overall
**Number of studies**	**6438**	**543**	**6981**
**GBD Super Region**			
High income	89%	20%	84%
Southeast Asia, East Asia, and Oceania	3%	11%	4%
Sub-Saharan Africa	1%	29%	3%
Multiple Regions #	1%	16%	2%
Latin America and Caribbean	1%	8%	2%
Central Europe, Eastern Europe, and Central Asia	1%	2%	1%
South Asia	0%	8%	1%
North Africa and Middle East	1%	2%	1%
NA	3%	3%	3%
**World Bank Income Category**			
Low-Income and Lower-Middle-Income	1%	43%	5%
Upper Middle-Income and High-Income	97%	37%	92%
Both	0%	17%	1%
None	2%	3%	2%
Intervention *			
Pharmaceutical	44%	32%	43%
Surgical	13%	8%	13%
Screening	12%	14%	12%
Care delivery	11%	17%	11%
Medical procedure	12%	4%	12%
Health education or behavior	9%	21%	10%
Immunization	6%	27%	8%
Other	19%	38%	20%
Study funder *			
Government	33%	47%	34%
Pharmaceutical or device company	28%	4%	27%
Foundation	10%	27%	11%
Healthcare organization ^	4%	9%	5%
None/Not determined	24%	24%	24%
Other	8%	20%	9%
Prevention stage *			
Primary	15%	59%	18%
Secondary	16%	20%	16%
Tertiary	62%	38%	60%
**Global Burden of Disease Category**			
Neoplasms	18%	3%	17%
Cardiovascular and circulatory diseases	17%	5%	16%
Diabetes, urogenital, blood, and endocrine diseases	12%	5%	11%
Other communicable, maternal, neonatal, and nutritional disorders	9%	7%	9%
Musculoskeletal disorders	10%	1%	9%
Mental and behavioral disorders	6%	8%	6%
HIV/AIDS and tuberculosis	4%	20%	6%
Digestive diseases	4%	1%	4%
Diarrhea, LRI, and other common infectious diseases	2%	20%	3%
Other	18%	31%	19%

Key: # “Multiple regions” refers to studies that reported cost-effectiveness estimates for countries in different regions. ^ Health care organizations include insurance companies, hospitals, HMOs, WHO. * Not mutually exclusive. GBD: Global burden of disease. GNI: Gross National Income. HMO: Health maintenance organization. LRI: Lower respiratory infection. WHO: World Health Organization.

**Table 2.** Standardized residual deviation from projected number of studies for each disease, by GBD region.

	GBD Region							Summary across all GBD Regions	
*Disease Area*	Asia and Oceania	Europe and Central Asia	High Income	Latin America and the Caribbean	North Africa and the Middle East	South Asia	Sub- Saharan Africa	Mean	Median (b)
Unintentional injury	-0.80	-0.81	-0.90	-0.85	-0.63	-0.81	-0.29	-0.72	-0.81
Transport injuries	-0.74	-0.94	-0.70	-0.98	-0.80	-0.65	-0.33	-0.73	-0.74
Liver Cirrhosis	-0.60	-0.96	-0.61	-0.89	-0.70	-0.69	-0.11	-0.65	-0.69
Neonatal Disorders	-0.65	-0.56	-0.25	-0.45	-0.73	-1.28	-1.55	-0.78	-0.65
Chronic Respiratory	-0.79	-0.59	-0.49	-0.28	-0.81	-0.91	-0.20	-0.58	-0.59
Nature, War, Legal	-0.49	-0.71	-0.24	-0.81	-0.69	-0.53	-0.04	-0.50	-0.53
Neurological Disorders	-0.53	-0.23	-0.87	-0.74	-0.17	-0.51	-0.03	-0.44	-0.51
Cardiovascular	-0.87	-0.98	1.51	0.67	-0.49	-0.89	0.14	-0.13	-0.49
Musculoskeletal	-0.63	-1.08	-0.31	-0.46	-0.47	-0.91	-0.33	-0.60	-0.47
Nutritional Deficiencies	-0.35	-0.43	-0.39	-0.66	-0.43	0.57	-0.49	-0.31	-0.43
Other, NCD	-0.38	-0.72	-1.13	0.05	-0.84	0.59	-0.34	-0.40	-0.38
Mental or behavior disorders	-0.61	0.84	-1.68	-0.91	-0.38	-0.13	-0.16	-0.43	-0.38
Maternal Disorders	-0.33	-0.52	0.00	-0.33	-0.32	0.51	0.46	-0.08	-0.32
Digestive Diseases	-0.25	-0.09	0.55	-0.74	-0.68	-0.57	0.04	-0.25	-0.25
NTD Malaria	-0.12	0.05	-0.20	0.01	0.83	0.09	0.05	0.10	0.05
Diabetes	0.75	1.14	1.61	0.21	0.50	-0.03	-0.57	0.51	0.50
Neoplasms	2.46	1.40	1.00	1.05	2.21	0.12	0.14	1.20	1.05
HIV and TB	1.23	0.64	0.84	1.53	0.51	2.52	3.83	1.59	1.23
Other Communicable, Maternal, Neonatal, or Nutrition	2.41	1.77	2.52	2.32	1.55	1.08	1.22	1.84	1.77
Diarrhea	1.28	2.39	-0.05	2.12	2.45	2.40	-1.66	1.27	2.12

Note: (a) Values reported are Studentized residuals. (b) Table presents diseases and conditions sorted by median deviation. The “unintentional injuries” category appears in the first table row because the median number of published studies was furthest below the corresponding projected number of studies by the greatest amount after standardization (Studentized residual of -0.81). The “diarrhea” category appears in the last table row because the median number of published studies exceeded the corresponding projected number of studies by the greatest amount after standardization (Studentized residual of 2.12). Abbreviations: NCD (non-communicable disease), NTD (neglected tropical disease), HIV (human immunodeficiency virus), TB (tuberculosis)

Based on these counts and proportions, we report the proportion of studies in each stratum, number of cost-per-QALY and cost-per-DALY studies published by year, proportion of published CEAs stratified by World Bank country income category and by study type (cost-per-QALY or cost-per-DALY), and number of cost-per-QALY and cost-per-DALY studies focusing on each country.

***Literature coverage vs. disease burden*.** We characterized the relationship between the number of CEA studies (cost-per-QALY plus cost-per-DALY) focusing on each disease and the corresponding disease burden by regressing within each of the seven GBD super regions CEA publication count against disease burden using ordinary least squares linear regression. Graphical plots of the regression results and original data for the three GBD regions with the most publications, and a table of standardized Studentized residuals for all seven regions (SAS Enterprise Guide version 7.1, Cary, NC) characterize which conditions are, in relative terms, over-studied or under-studied in each region, compared to the other conditions.

## Results

**Figure 1.**
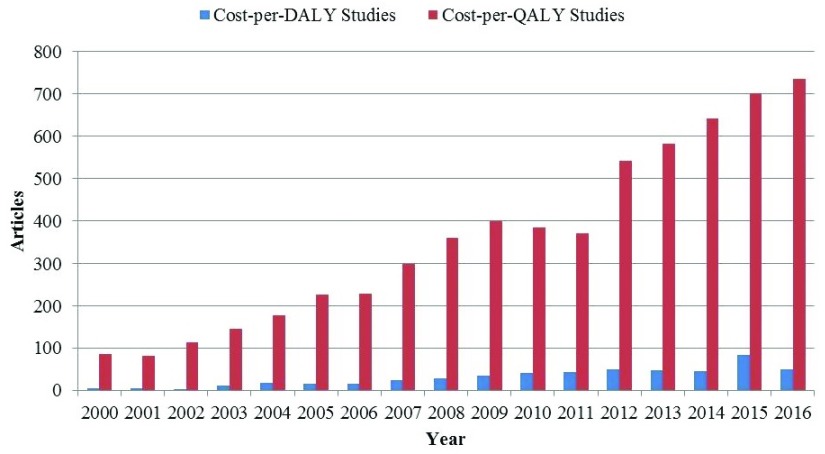
Published cost-per-DALY and cost-per-QALY studies by year. Journals published 360 cost-per- QALY studies during the years 1976 through 2000. Journals published 13 cost-per- DALY studies during the years 1995 through 2000.

We identified 6,438 cost-per-QALY ( Dataset 1) and 543 cost-per-DALY ( Dataset 2) studies published through 2016. The number of published studies in the cost-per-QALY and cost-per-DALY literatures has increased steadily since 2000 ( Figure 1).

### Study characteristics

**Figure 2.**
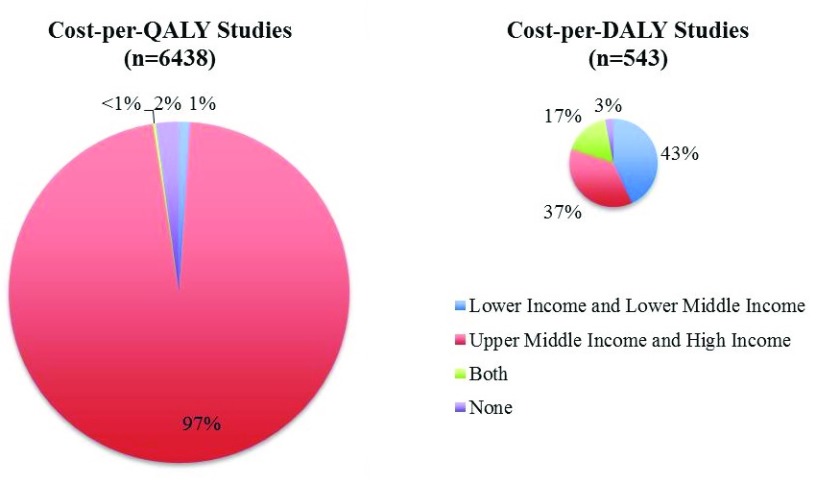
Cost-per-QALY vs. cost-per-DALY studies by world bank income level. The area of each pie chart is proportional to the number of studies catalogued in each registry.

**Figure 3.**
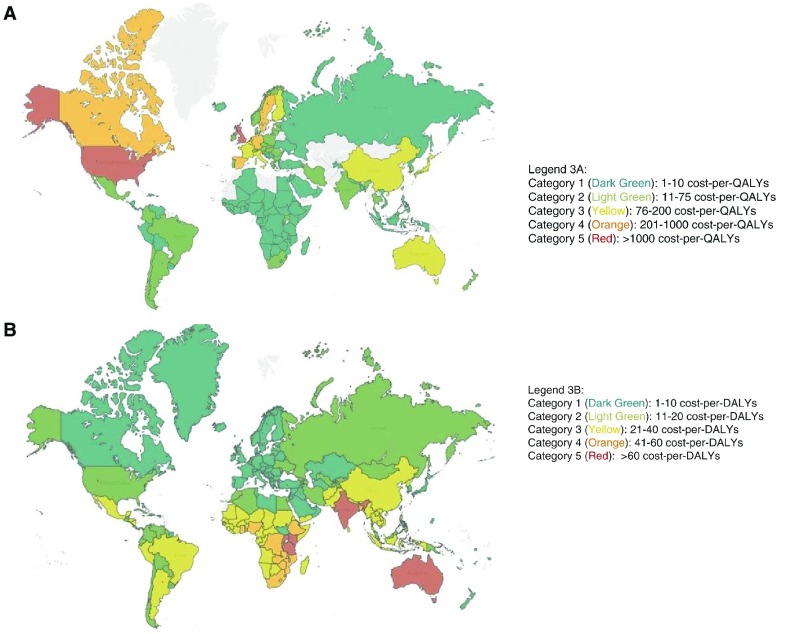
Geographic distribution of Cost-per-QALY and Cost-per-DALY studies. The maps present the number of cost-per-QALY studies ( Figure 3A) and cost-per-DALY studies ( Figure 3B) for each country. Gray indicates countries with no associated studies. If a study reported a cost-effectiveness estimate for two or more countries, we counted a CEA for each country (e.g. if a study reported an intervention’s cost-effectiveness ratio for both Canada and the United States, we incremented the study count in both countries). If a study reported a “global” cost-effectiveness ratio, we excluded it from all country counts. We also excluded from these counts studies that did not clearly specify an applicable country or region.

Cost-per-QALY studies have tended to focus on upper-middle income and high-income countries (97%); e.g. 2,321 studies focus on the United States, while 1,149 studies focus on the United Kingdom. Cost-per-DALY studies have focused to a much greater extent on low and lower-middle income countries (43%); e.g. 95 studies focus on India, 51 focus on China, and 90 studies focus on Uganda ( Table 1, Figure 2, Figure 3A and 3B).

Tertiary prevention (treatment) dominates the cost-per-QALY registry (62%), whereas the cost-per-DALY registry focuses far more on primary prevention (59%). Conditions most frequently addressed by studies in the cost-per-QALY literature include non-communicable diseases, such as cancer (18%) and cardiovascular diseases (17%), whereas most cost-per-DALY registry studies target infectious diseases.

Foundations are the single largest source of non-governmental support for cost-per-DALY studies (27%), while pharmaceutical and device companies are the single largest source of non-governmental support for cost-per-QALY studies (28%).

We classified countries into the following World Bank income categories (quantities expressed in 2016 US dollars): low-income (GNI per capita < $1,005), lower-middle income (GNI per capita of $1,006 – $3,955), upper-middle income (GNI per capita of $3,956 – $12,235), and high-income (GNI per capita > $12,235) ^18^. We used GBD Super Region definitions reported in the 2015 GBD study ^17^.

In Figure 3A, we excluded one study classified as “international.” We excluded 145 studies because the country of study was unclear.

In Figure 3B, we excluded 13 studies classified as “international.” We excluded 17 studies because the country of study was unclear.

### Literature coverage vs. disease burden

**Figure 4.**
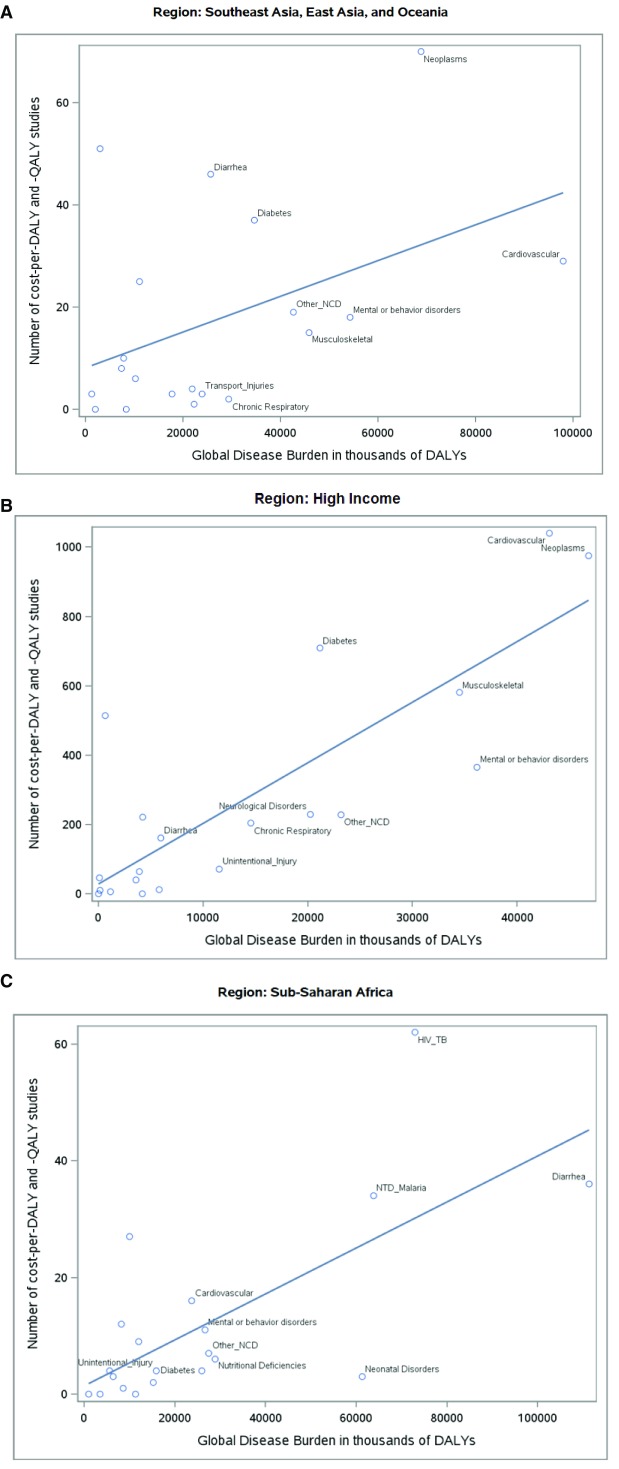
Number of CEAs vs. normalized disease burden for selected diseases and GBD Super Regions. ( **A**) Southeast Asia, East Asia, and Oceania. ( **B**) High Income Countries. ( **C**) Sub-Saharan Africa.

Neoplasms were the most studied diseases in Southeast Asia, East Asia, and Oceania ( Figure 4A), while mental and behavioral disorders were less studied relative to their burden. High-income countries had relatively few studies addressing mental and behavioral disorders, and injuries ( Figure 4B). Relative to burden, HIV/AIDS and tuberculosis were the most studied diseases in Sub-Saharan Africa, while this region reported fewer studies on nutritional deficiencies ( Figure 4C). 

Table 2 reports Studentized residuals from the ordinary least square regression for each region, along with the average and median of these residuals for each disease, across all seven GBD regions. Those results suggest that a number of conditions are uniformly “under-studied” because the residuals are negative in all seven regions (e.g., unintentional injuries, transport injuries, liver cirrhosis). Positive residuals across most regions indicate other conditions generally receive more attention than appears warranted by their burden (HIV and TB, neoplasms).

Each Figure 4 panel displays results for the top 10 diseases and includes a diagonal line that represents average studies published as a function of disease burden for each Super Region. The location of a plotted point to the “northwest” of this line indicates a disease that is relatively “over-studied” within that region, because the number of published studies exceeds, on average, the number published studies for other diseases imposing the same burden on the population. The location of a plotted point to the “southeast” indicates a disease that is relatively under-studied.

## Discussion

Our review reveals a notable increase in the publication of cost-per-QALY and cost-per-DALY studies since 2000, thus making ever more cost-effectiveness information available to aid decision makers in their efforts to prioritize resources. The literature spans a wide range of interventions, diseases, and geographic regions.

The data demonstrate key differences between the cost-per-QALY and cost-per-DALY literatures ( Table 1). For example, the cost-per-QALY literature tends to focus on high-income countries, while cost-per-DALY studies focus more on lower- and middle-income income nations. Differences extend to the types of interventions and diseases represented: cost-per-QALY studies tend to address diseases prevalent in wealthier countries (e.g., cardiovascular disease and cancer), while cost-per-DALY studies address diseases more prevalent in low-income countries (e.g., infectious diseases, such as tuberculosis and HIV). The two literatures also differ in terms of the interventions on which they focus. More cost-per-QALY studies evaluate pharmaceuticals, while cost-per-DALY studies focus more often on immunizations.

Several factors may explain why cost-per-QALY studies predominate in high-income countries, while cost-per-DALY studies are more popular in lower and middle-income countries. The differences could, for example, reflect the availability of health utility weights, needed to estimate QALYs, in high-income countries and the lack of such information in lower-income settings. Researchers conducting CEAs in countries with limited data capacity may find it easier and less expensive to use the cost-per-DALY metric. 

The differences could also reflect the preferences and traditions of organizations that fund CEA studies. Foundations funding global health research may prefer the DALY metric, given the historic use of DALYs to measure global disease burden. In contrast, health authorities in high-income countries (e.g., the National Institute for Health and Care Excellence (NICE) in the United Kingdom) have tended to recommend the use of QALYs in CEAs. The geographic differences between the cost-per-QALY and cost-per-DALY literature deserve further investigation, as our effort did not gather information on why authors used these measures.

Our data also indicate inconsistencies between literature coverage and disease burden. Some diseases and conditions (e.g., cardiovascular disease and mental health in Southeast Asia, South Asia and Oceania) are relatively “under-studied,” while other diseases and conditions (e.g., HIV and TB in all regions) are relatively “over-studied”. 

There is no clear explanation for these inconsistencies. As we have noted elsewhere, decisions to fund or conduct economic evaluations reflect not just the disease burden imposed by the targeted condition, but also the number of promising interventions or programs ^19, 20^. Because specialty drugs for diseases such as cancer represent important new interventions in high-income countries, and because pharmaceutical companies have the resources and incentive to characterize value for those interventions, much of the cost-per-QALY literature has recently focused on specialty drug therapies. These financial incentives are less pronounced in the lower- and middle-income countries that are much more the focus of the cost-per-DALY literature. In addition to disease burden, priorities in the cost-per-DALY literature may reflect the visibility and emotional salience of diseases, the influence of advocacy groups, the vagaries of reimbursement decisions ^19^, and institutional priorities of the organizations sponsoring the research.

In any case, the incongruities we observed between literature coverage and disease burden raise important questions about opportunities for the re-direction of future CEA research funding so that resources for such research can generate the highest return on investment.

Our work has the following limitations. First, the databases we used are restricted to English-language articles indexed in PubMed. This restriction may have depressed the number of cost-per-DALY studies we identified to a greater extent proportionally than it may have depressed the number of cost-per-QALY studies we identified because a smaller proportion of the cost-per-DALY literature focuses on English-speaking countries. Second, categorizing studies (e.g., whether an intervention targets primary or secondary prevention) depends on judgment, and other researchers may have classified articles differently. 

In the future it will be important to further explore trends in the CEA literature in terms of diseases and geographic regions covered, funding patterns among donor organizations, the country of origin or study authors, the prevalence and patterns of CEAs published in languages other than English, the variation in methods used in analyses, and whether published studies address society’s most pressing needs ^21^. It will also be useful to continue to investigate the methodological underpinnings of QALYs and DALYs and how much the choice of metric influences CEA results and the decisions based on them ^22, 23^.

## Data availability

We have made the data used in this analysis available through the Open Science Foundation (OSF): http://doi.org/10.17605/OSF.IO/3BEK5 ^24^.

License: CC0 1.0 Universal.

**Dataset 1. Cleaned QALY Database.**

Includes the cost-per-QALY data used in this paper.

**Dataset 2. Cleaned DALY Database.**

Includes the cost-per-QALY data used in this paper.

**Dataset 3. Regional and disease level stratification dataset.**

Includes disease burden and literature coverage data used in this paper.

## Supplementary material

**Supplementary File 1. Cost-per-QALY manual.** Documents the variables collected in the cost-per-QALY database.

**Supplementary File 2. Cost-per-DALY manual.** Documents the variables collected in the cost-per-DALY database.
