# Supplementary material for: Comparing the cost-per-QALYs gained and cost-per-DALYs averted literatures
Source: Gates Open Res. 2018 Mar 5;2:5. Originally published 2018 Jan 18. [Version 2] doi: 10.12688/gatesopenres.12786.2 (PMC5801595; doi:10.12688/gatesopenres.12786.2)
Supplement: Supplementary file 1 [file gatesopenres-2-13870-s0000.tgz › e34fabe1-5e24-409f-aab0-6f9c4fda054b.pdf]

CEVR
Center for the Evaluation of Value and Risk in Health

Tufts Medical Center
Institute for Clinical Research & Health Policy Studies

The Cost-Effectiveness Analysis (CEA) Registry

User Guide

2016

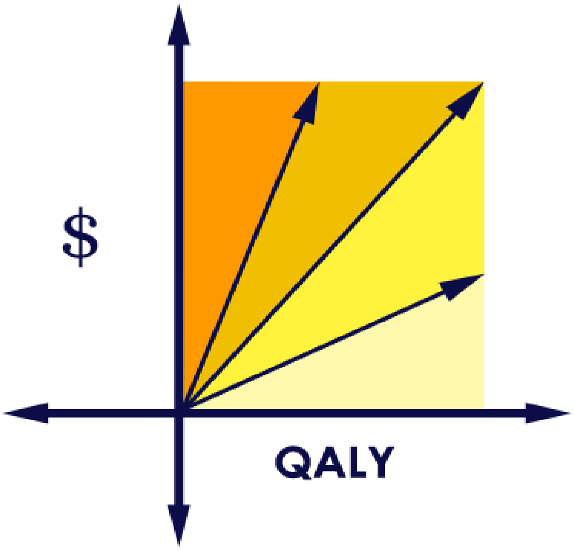

www.cearegistry.org

Center for the Evaluation of Value and Risk in Health
Institute for Clinical Research & Health Policy Studies, Tufts Medical Center
800 Washington Street, #063
Boston, MA 02111

CONTENTS

I. BACKGROUND 3

ABOUT THE CEA REGISTRY 4

DATABASE STRUCTURE 5

METHODOLOGY 6

SEARCH STRATEGY 8

II. DATA GUIDE 9

VARIABLE DICTIONARY 10

Article Information 10
Disease Classification 10
Methods Information 13
Ratio Information 17
Ratio calculation 17
Weights form 19

III. GLOSSARY 20

DISEASE CLASSIFICATION INFORMATION 21

METHOD INFORMATION 23

RATIO INFORMATION 30

UTILITY WEIGHTS INFORMATION 34

IV. PUBLICATIONS 37

I. BACKGROUND

About The CEA Registry

The Center for the Evaluation of Value and Risk in Health at the Institute for Clinical Research and Health Policy Studies, Tufts Medical Center, Boston, MA developed the Cost-Effectiveness Analysis (CEA) Registry. The Registry is a unique database containing detailed information on over 3,600 cost-utility analyses published up to 2012. Health-related CEAs estimate the resources used (costs) and the health benefits achieved (effects) for an intervention compared to an alternative treatment strategy. The Registry focuses on a subset of CEAs, called cost-utility analyses (CUAs) that quantify health benefits in terms of Quality Adjusted Life Years (QALYs), a metric that accounts for changes in both longevity and quality of life.

The objectives of the Registry are to help decision makers identify society's best opportunities for targeting resources to improve health, to assist policymakers in healthcare resource allocation decisions, and to move the field towards the use of standard methodologies.

The CEA Registry contains data on articles published from 1976 through 2015 and is updated frequently, including information on about 10,300 ratios. Its contents allow for benchmark and trend analysis, and identification of cost-effectiveness information for a wide range of interventions. The CEA Registry has been the source of data for over 40 peer-reviewed papers. It has been used or cited in analyses performed by the Environmental Protection Agency, the Food and Drug Administration, the Institute of Medicine, and the Medicare Payment Assessment Commission. The CEA Registry is also listed on the National Library of Medicine's website as an important health economics resource.

DATABASE STRUCTURE

Figure 1 illustrates the hierarchical structure of the CEA Registry database. A hierarchical structure is useful in cases where there is a one-to-many relationship between different elements of the data. The CEA Registry has four main sheets: the article sheet, ratio sheet, the utility weight sheet, and the variables sheet.

- Article sheet: contains one record for each article.
- Ratio sheet: contains one or more ratio records for each article.
- Utility Weight sheet: contains none or one or more utility weight records for each article.
- Variables Sheet: contains a listing and description of all included variables.

Figure 1 illustrates the Registry's contents for a single article that has reports information for *N* many ratios and *M* many utility weights.

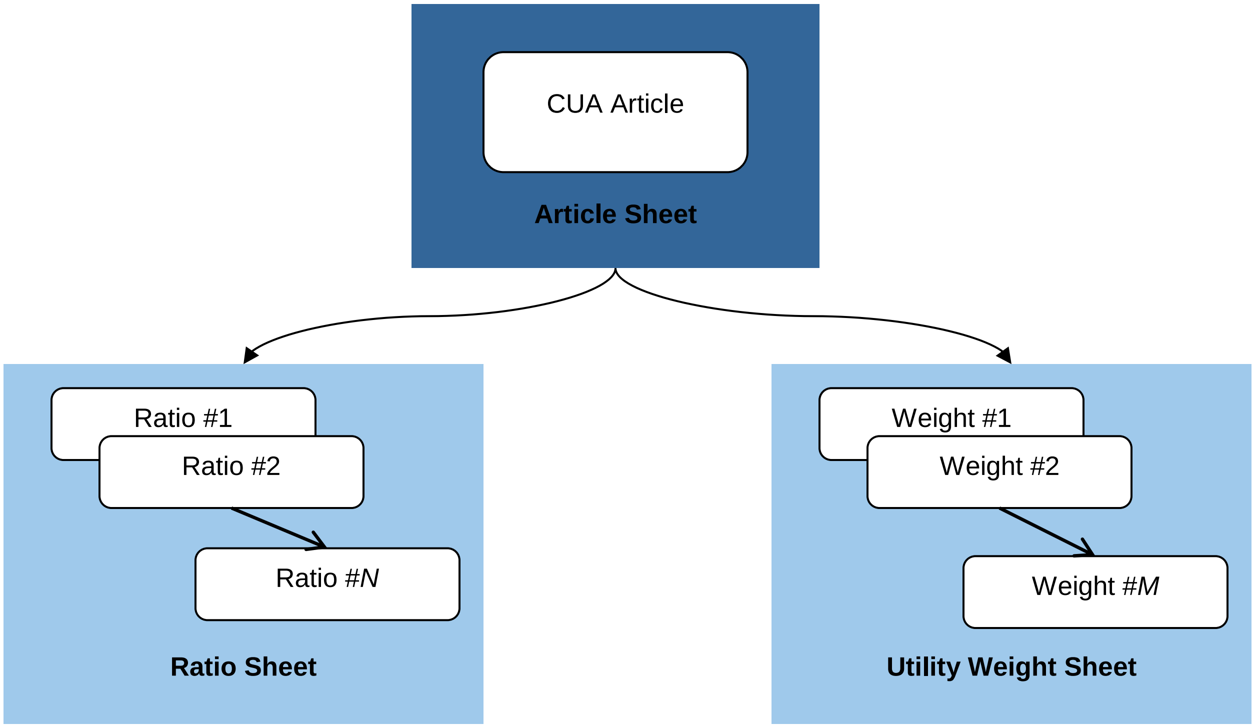

Figure 1 CEA Registry Hierarchical Structure

METHODOLOGY

The published articles summarized in the Registry undergo a formalized review protocol (Figure 2). The analyses address a wide variety of diseases and treatments and all measure health effects in terms of quality-adjusted life years (QALYs). The QALY is a standard measure that accounts for quality of life (morbidity) and longevity (mortality). The CEA Registry team searches MEDLINE for English-language articles using keywords "QALYs", "quality-adjusted", and "cost-utility analysis". Abstracts from these articles are screened to determine if the paper contains an original cost-utility estimate. We exclude review, editorial, or methodological articles, as well as cost-effectiveness analyses that do not use QALYs to quantify health benefits.

Each article meeting these criteria is assigned a disease classification by a clinician. Two readers with training in decision analysis and cost-effectiveness analysis independently review each article and record information using a standardized set of forms and instructions. The two readers convene for a consensus audit to resolve any potential discrepancies. On occasion, a third reader may be called upon to help settle disputed items.

Data on over 40 variables are collected for each article.

ARTICLE INFORMATION

We report: 1) the type of intervention evaluated; 2) the country of the analysis; 3) the funding source.

For methodology, we report 1) whether the article correctly calculated incremental cost-utility ratios; 2) the analytic time horizon and analytic perspective (e.g., societal or health care payer); 3) what discount rate, if any, was used; 3) the currency used; 4) whether the analysis accounted for additional costs associated with greater longevity achieved through treatment; 5) the type of sensitivity or uncertainty analysis used; 6) whether the article specified a threshold for identifying acceptably favorable cost-effectiveness ratios; and 7) a subjective assessment regarding the article's overall quality on an interval scale from 1 (low) to 7 (high) 8) a subjective assessment regarding the execution of the following three areas of the research: (i) the health economic methodology, (ii) the model's and/or papers consideration and communication of uncertainty, (iii) the transparency of the model used in the analysis.

RATIO INFORMATION

We describe the health intervention that is the subject of the analysis, and comparator intervention to which it is compared, and the population that is eligible for the intervention. When available, we also report the costs and health benefits (QALYs) associated with both the target and comparator interventions. We report the value of the ratio reported in the original article, as well as the value we calculate directly from the cost and health benefit information in the article. We also report the ratio quadrant (1= more costly, less effective; 2= more costly, more effective; 3= less costly, less effective; and 4= less costly, more effective) (as shown in figure #3, pg. #39).

UTILITY WEIGHT INFORMATION

We report the health condition and demographics of the considered population (e.g. sex, age, and comorbidities), utility weight value, and range of plausible values. We report, when used, secondary literature sources relied upon to provide utility weight values. In cases where the authors develop their own utility weight values, we describe the methodology employed (e.g. SF-36, TTO, VAS, etc.).

SEARCH STRATEGY

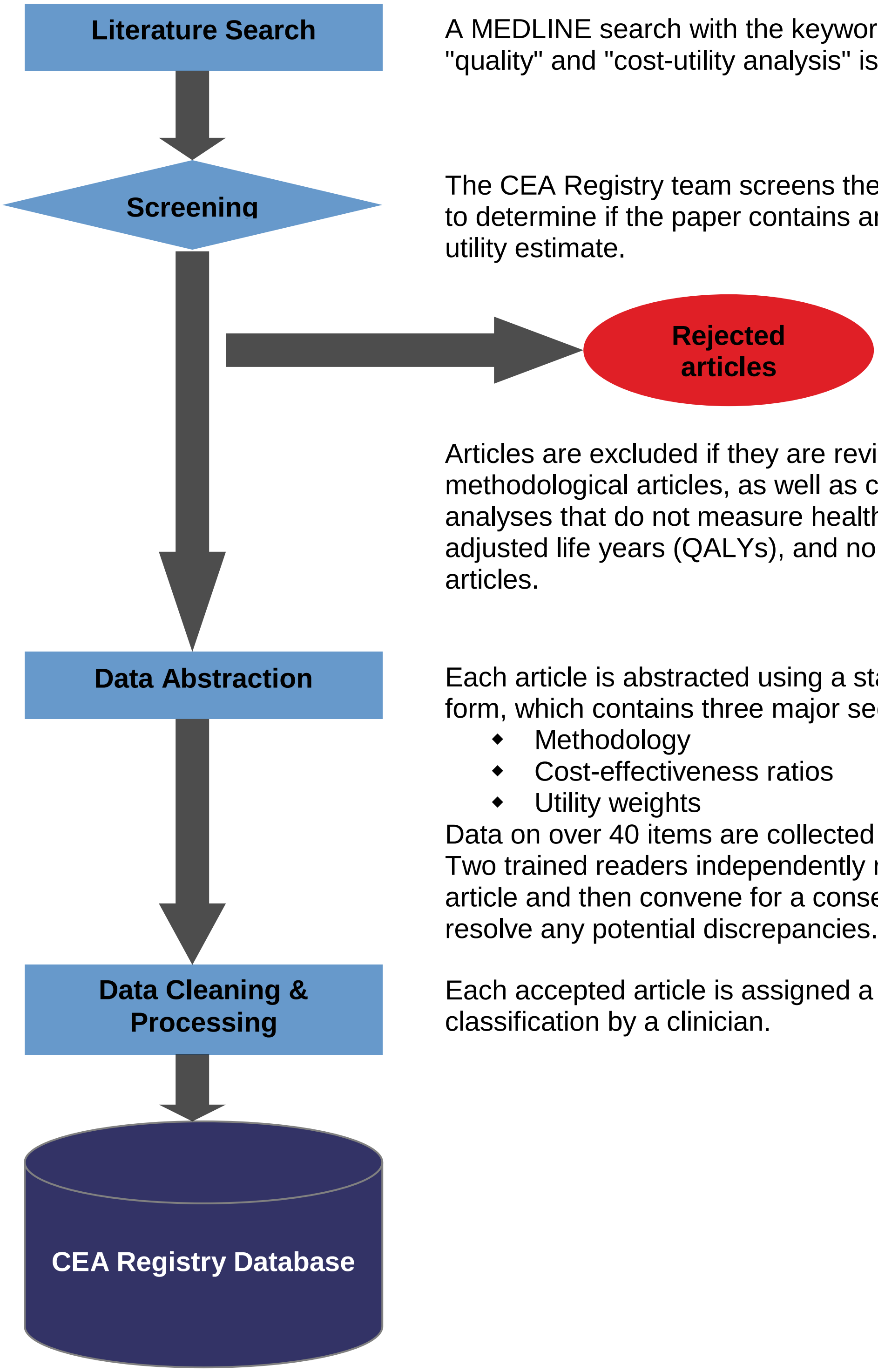

A MEDLINE search with the keywords, "QALYs", "quality" and "cost-utility analysis" is conducted.

The CEA Registry team screens the article abstracts to determine if the paper contains an original cost-utility estimate.

Articles are excluded if they are review, editorial, or methodological articles, as well as cost-effectiveness analyses that do not measure health effects in quality-adjusted life years (QALYs), and non-English language articles.

Each article is abstracted using a standard data auditing form, which contains three major sections:

- Methodology
- Cost-effectiveness ratios
- Utility weights

Data on over 40 items are collected for each article. Two trained readers independently read and audit each article and then convene for a consensus audit to resolve any potential discrepancies.

Each accepted article is assigned a disease classification by a clinician.

Figure 2: Search Strategy

II. DATA GUIDE

VARIABLE DICTIONARY

*Txt=Text, Num=Numeric

Items	Variables	Description	Data type*	Coding
**Article Information**				
	ArticleID	CEA Registry article's ID number	Txt	11 digits, <<xxxx-xx-xxxxx>>
	PubMedID	Medline ID number	Num	
	Title	Title of article	Txt	
	PrimAuthLastName	Primary author's last name	Txt	
	PrimAuthFirstName	Primary author's first name	Txt	
	JournalAbbr	Journal abbreviation	Txt	
	JournalVolume	Journal volume	Txt	
	JournalIssue	Journal Issue	Txt	
	JournalPages	Journal pages	Txt	
	PubDate	Published date	Txt	
**Disease Classification**				
**Study Theme**				
	Theme PublicHealth	Public health related topics	Num	0=False, 1=True
	Theme Men	Men's health related topics	Num	0=False, 1=True
	Theme Women	Women's health related topics	Num	0=False, 1=True
	Theme Elderly	Elderly's health related topics	Num	0=False, 1=True
	Theme Children	Children's health related topics	Num	0=False, 1=True
	Theme NoneNS	None of these topics	Num	0=False, 1=True
**Primary Disease**				
	PrimaryDiseaseID	Primary disease category ID number	Num	
	OtherDiseaseBlank	Other primary disease	Txt	
**Secondary Disease(s)**				
	DID1	Infectious	Num	0=False, 1=True
	DID-2	Malignant Neoplasms	Num	0=False, 1=True
	DID-3	Endocrine Disorders	Num	0=False, 1=True
	DID-4	Neuro-Psychiatric and Neurological Conditions	Num	0=False, 1=True
	DID-5	Sense Organ Diseases	Num	0=False, 1=True
	DID-6	Cardiovascular Diseases	Num	0=False, 1=True
	DID-7	Respiratory Diseases	Num	0=False, 1=True
	DID-8	Digestive Diseases	Num	0=False, 1=True
	DID-9	Genito-Urinary Diseases	Num	0=False, 1=True
	DID10	Musculoskeletal and Rheumatologic Diseases	Num	0=False, 1=True
	DID11	Maternal and Child Health (Perinatal)	Num	0=False, 1=True
	DID12	Congenital Anomalies	Num	0=False, 1=True
	DID13	Nutritional Deficiencies	Num	0=False, 1=True
	DID14	Anemias	Num	0=False, 1=True

Items	Variables	Description	Data type*	Coding
	DID15	Skin Diseases (Non-Cancer)	Num	0=False, 1=True
	DID16	Dental/Oral Conditions	Num	0=False, 1=True
	DID17	Injuries/Exposures	Num	0=False, 1=True
	DID18	Critical Care	Num	0=False, 1=True
	DID19	Allergy/Immunology	Num	0=False, 1=True
	DID-20	Hematology - Other	Num	0=False, 1=True
	DID-21	Program/Organizational Interventions	Num	0=False, 1=True
	DID-22	Patient-Centeredness	Num	0=False, 1=True
	DID-23	Other	Num	0=False, 1=True
	DID-24	Other Secondary	Num	0=False, 1=True
	DID110	Tuberculosis	Num	0=False, 1=True
	DID111	STDs excluding HIV	Num	0=False, 1=True
	DID112	HIV/AIDS	Num	0=False, 1=True
	DID113	Respiratory Infections	Num	0=False, 1=True
	DID114	Otitis Media	Num	0=False, 1=True
	DID115	Other Infectious Diseases	Num	0=False, 1=True
	DID120	Colorectal Cancer	Num	0=False, 1=True
	DID121	Lung Cancer	Num	0=False, 1=True
	DID122	Breast Cancer	Num	0=False, 1=True
	DID123	Cervical Cancer	Num	0=False, 1=True
	DID124	Uterine Cancer	Num	0=False, 1=True
	DID125	Ovary Cancer	Num	0=False, 1=True
	DID126	Prostate Cancer	Num	0=False, 1=True
	DID127	Hematologic Cancers (Lymphomas, Leukemia)	Num	0=False, 1=True
	DID128	Other Neoplasms	Num	0=False, 1=True
	DID130	Diabetes Mellitus	Num	0=False, 1=True
	DID131	Other Endocrine	Num	0=False, 1=True
	DID140	Depression and Bipolar Affective Disorder	Num	0=False, 1=True
	DID141	Schizophrenia	Num	0=False, 1=True
	DID142	Seizure Disorders (Epilepsy)	Num	0=False, 1=True
	DID143	Alzheimer's and Other Dementias	Num	0=False, 1=True
	DID144	Parkinson’s Disease	Num	0=False, 1=True
	DID145	Multiple Sclerosis	Num	0=False, 1=True
	DID146	Substance Abuse Disorders	Num	0=False, 1=True
	DID147	Other Neuro-Psychiatric and Neurological Disorders	Num	0=False, 1=True
	DID150	Vision	Num	0=False, 1=True
	DID151	Hearing	Num	0=False, 1=True
	DID160	Ischemic Heart Disease	Num	0=False, 1=True
	DID161	Non-Ischemic Heart Disease - Other	Num	0=False, 1=True
	DID162	Cerebrovascular Disease	Num	0=False, 1=True
	DID163	Hypertension	Num	0=False, 1=True
	DID164	Lipids	Num	0=False, 1=True
	DID165	Vascular, Non-Cardiac, Non-Cerebral	Num	0=False, 1=True

Items	Variables	Description	Data type*	Coding
	DID170	COPD	Num	0=False, 1=True
	DID171	Asthma	Num	0=False, 1=True
	DID172	Other Respiratory	Num	0=False, 1=True
	DID180	Peptic Ulcer Disease	Num	0=False, 1=True
	DID181	Cirrhosis of the Liver	Num	0=False, 1=True
	DID182	Other Non-Infectious GI Diseases	Num	0=False, 1=True
	DID190	Kidney Disease	Num	0=False, 1=True
	DID191	Non-Cancer Prostate Disease	Num	0=False, 1=True
	DID192	Other Genito-Urinary	Num	0=False, 1=True
	DID-200	Rheumatoid Arthritis	Num	0=False, 1=True
	DID-201	Osteoarthritis	Num	0=False, 1=True
	DID-202	Other Musculoskeletal	Num	0=False, 1=True
	DID-203	Other Rheumatologic	Num	0=False, 1=True
	DID-255	None of the Above/Not Applicable	Num	0=False, 1=True

Methods Information				
**Author Email**				
	CorrespondingEmail	Primary author's email	Txt	
**Study Type**				
		Cost per QALY study	Num	0=False, 1=True
		Cost per life year gained study	Num	0=False, 1=True
**Intervention Types**				
	Intervention CareDelivery	Development of facility or distribution of personnel	Num	0=False, 1=True
	Intervention Diagnostic	A method used to determine if and what type of disease is present	Num	0=False, 1=True
	Intervention HealthEducationOrBehavior	An intervention designed to educate individuals on behaviors that promote, maintain or restore health	Num	0=False, 1=True
	Intervention Immunization	Receipt of vaccination	Num	0=False, 1=True
	Intervention MedicalDevice	May or may not require a surgical or implantation procedure	Num	0=False, 1=True
	Intervention MedicalProcedure	Non-surgical, non-diagnostic procedures	Num	0=False, 1=True
	Intervention Pharmaceutical	Any drug or biotech product used for medical treatment or prevention	Num	0=False, 1=True
	Intervention Screening	Refers to measures that detect disease before it is symptomatic	Num	0=False, 1=True
	Intervention Surgical	Invasive; cutting involved	Num	0=False, 1=True
	Intervention Other	Any intervention not described above	Num	0=False, 1=True
	Intervention OtherText	If other, text of intervention	Txt	
	Intervention NoneNA	None/Non-applicable	Num	0=False, 1=True
**Prevention Stage**				
	PreventionStage	Divided into 3 stages, primary, secondary and tertiary	Num	1=Primary, 2=Secondary, 3=Tertiary
**Country of Study**				
	Country	1st country name	Txt	
	Country2	2nd country name	Txt	
	Country3	3rd country name	Txt	
	Country4	4th country name	Txt	
	Country5	5th country name	Txt	
	Country6	6th country name	Txt	
**Affiliation of Author(s)**				
	AuthorAffiliation Academic	Universities, institutes/centers or any other academic organizations	Num	0=False, 1=True
	AuthorAffiliation HealthCare	Health care organizations	Num	0=False, 1=True
	AuthorAffiliation Government	Governmental organizations	Num	0=False, 1=True
	AuthorAffiliation PharmOrDevice	Industrial pharmaceutical companies, biotech companies and medical device companies.	Num	0=False, 1=True
	AuthorAffiliation Consultant	Contract researcher/consultant	Num	0=False, 1=True
	AuthorAffiliation Other	Other than the choices above	Num	0=False, 1=True
	AuthorAffiliation OtherText	If other, name of author's affiliation	Txt	
	AuthorAffiliation NoneNS	No affiliation information provided in the article	Num	0=False, 1=True
**Study sponsorship/funding**				
	Sponsorship Government	Governmental organizations	Num	0=False, 1=True

	Sponsorship Foundation	A type of nonprofit organization	Num	0=False, 1=True
	Sponsorship PharmOrDevice	Industrial pharmaceutical companies, biotech companies and medical device companies.	Num	0=False, 1=True
	Sponsorship PharmOrDeviceCoName	Name of funding pharmaceutical or medical device company	Txt	
	Sponsorship HealthCare	Health care organizations	Num	0=False, 1=True
	Sponsorship ProfMembOrg	Professional membership organization, Membership organizations of professional persons formed for the advancement of the interests of their profession	Num	0=False, 1=True
	Sponsorship None	Paper explicitly states no funding	Num	0=False, 1=True
	Sponsorship NotDetermined	No information provided	Num	0=False, 1=True
	Sponsorship Other	Other than the choices above	Num	0=False, 1=True
	Sponsorship OtherText	If other, name of the sponsorship	Txt	
**Time Horizon**				
	TimeHorizonStatedID	Stated time horizon clearly	Num	1=Yes, 0=No
	TimeHorizonMultipleID	Multiple time horizons	Num	1=Yes, 0=No
	TimeHorizonUnitsID	The unit of time horizon (base case)	Num	0=Lifetime, 1=Weeks, 2=Months, 3=Years
	TimeHorizonMagnitude	Number of time unit	Num	Positive value
	TimeHorizonMultiple Lifetime	Multiple horizon, lifetime	Num	0=False, 1=True
	TimeHorizonMultiple Other	Multiple horizon, other	Num	0=False, 1=True
	TimeHorizonMultiple OtherText	If other, state time horizon	Txt	
**Perspective**				
	PerspectiveCorrect	Was perspective accurately identified?	Num	1=Yes, 0=No
	AuthorPerspectiveID	Perspective stated by author(s) in the article	Num	1=Societal, 2=Health care payer, 3=Limited societal, 4=Health care sector, 254=Not stated/could not determine, 250=Other
	AuthorOtherPerspectiveText	If other, the perspective stated by author(s)	Txt	
	ReaderPerspectiveID	Perspective judged by reader	Num	1=Societal, 2=Health care payer, 3=Limited societal, 4=Health care sector, 254=Not stated/could not determine, 250=Other
	ReaderOtherPerspectiveText	If other, the perspective judged by reader	Txt	
**Costs Included**				
	Costs Healthcare	Costs associated directly with the treatment	Num	0=False, 1=True
	Costs DirectMedical	Subcategory for direct medical costs	Num	0=False, 1=True
	Costs OutofPocket	Subcategory for out of pocket costs	Num	0=False, 1=True
	Costs Nonhealthcare	Costs not incurred by the health care sector	Num	0=False, 1=True
	Costs PatientTime	Subcategory for costs related to patient time	Num	0=False, 1=True
	Costs CaregiverTime	Subcategory for costs related to caregiver time	Num	0=False, 1=True
	Costs Transportation	Subcategory for costs related to transportation to a healthcare facility	Num	0=False, 1=True
	Costs ProductivityGains	Subcategory for costs related to productivity gain/loss	Num	0=False, 1=True
	Costs IncomeLoss	Subcategory for costs related to amount loss in income	Num	0=False, 1=True
	Costs NonhealthcareOther	Other non-health care costs	Num	0=False, 1=True
	Costs NonhealthcareOtherText	State descriptions of non-health care costs	Txt	

	Costs OtherSectors	Costs associated with other sectors (spillover effect)	Num	0=False, 1=True
	Costs PublicHealth	Subcategory for costs related to public health resources	Num	0=False, 1=True
	Costs Legal	Subcategory for costs related to justice system	Num	0=False, 1=True
	Costs Education	Subcategory for costs related to education	Num	0=False, 1=True
	Costs Housing	Subcategory for costs related to housing	Num	0=False, 1=True
	Costs Environment	Subcategory for costs related to environmental impact	Num	0=False, 1=True
	Costs SectorsOther	Other sectors	Num	0=False, 1=True
	Costs SectorsOtherText	State descriptions of costs included for other sectors	Txt	
	Costs NotDetermined	Not enough information provided	Num	0=False, 1=True
**Discounting Rate**				
	CostsDiscountedID	Were costs discounted?	Num	0=No, 1=Yes, 2=Not applicable, 3=Could not determine
	CostsDiscountRate	If yes, the discounting rate of costs	Num	
	QALYsDiscountedID	Were QALYs discounted?	Num	0=No, 1=Yes, 2=Not applicable, 3=Could not determine
	QALYsDiscountRate	If yes, the discounting rate of QALYs	Num	
**Study Currency**				
	CurrencyCountry	Currency of CE ratios	Txt	
	YearOfCurrency	The year of currency	Num	
	CurrencyYearStatedID	If the currency and year are not given, the readers assume the currency year is 2 years prior to article publication	Num	0=Estimated by reader at 2 years prior to article published, 1=Stated by author
**Incremental Analysis were**				
	IncrementalAnalysis NotReported	Incremental analyses were not reported.	Num	0=False, 1=True
	IncrementalAnalysis Correct	The incremental analysis was conducted and calculated correctly	Num	0=False, 1=True
	IncrementalAnalysis Incorrect	The incremental analysis was conducted but calculated incorrectly	Num	0=False, 1=True
	IncrementalAnalysis Recalculated	If incremental analysis was calculated incorrectly and there were sufficient data of cost and effectiveness; readers recalculated the analysis	Num	0=False, 1=True
	IncrementalAnalysis Incalculable	Sufficient data were not provided in the study to repeat the ICER calculation	Num	0=False, 1=True
**Cost-effectiveness threshold**				
	Cutoff 20K	US$20,000	Num	0=False, 1=True
	Cutoff 50K	US$50,000	Num	0=False, 1=True
	Cutoff 100K	US$100,000	Num	0=False, 1=True
	Cutoff Laupacis	Refers to a landmark article in the field (Laupacis A et al. Canadian Medical Association Journal 1992)	Num	0=False, 1=True
	Cutoff Range	A range in US$ is provided as the threshold	Num	0=False, 1=True
	Cutoff RangeLower	Lower value of the range	Num	Any numeric value
	Cutoff RangeUpper	Upper value of the range	Num	Any numeric value
	Cutoff Other	A US$ amount that is not US$20K, 50K or 100K	Num	0=False, 1=True

	Cutoff OtherText	Other value of threshold in US dollars	Txt	
	Cutoff OtherNonUs	Any range or value that is given in a currency other than US$	Num	0=False, 1=True
	Cutoff OtherNonUsText	Other non-US currency value	Txt	
	Cutoff NoneNS	Not stated the threshold	Num	0=False, 1=True
	AcceptabilityCurvesID	Did the analyses include acceptability curves?		
**Overall quality of analysis**				
	Rating	Overall quality of analysis (judged by reader)	Num	1, 1.5, 2, 2.5, 3, 3.5, 4, 4.5, 5, 5.5, 6, 6.5, 7
**Comments**				
	GeneralComments	General comments	Txt	

**Ratio Information**				
**Target Population**				
	Target Population	The population that was evaluated in the study (for this specific ratio)	Txt	
	TargetPopulation HealthID	Health status of target population	Num	1=General, 2=Specific disease
	TargetPopulation HealthText	Specific disease statement	Txt	
	TargetPopulation Gender	Gender	Num	1=Female, 2=Male, 3=Both, 4=Not Specified
	TargetPopulation Age_018	Pediatric, age 018	Num	0=False, 1=True
	TargetPopulation Age_1940	Age 18-50	Num	0=False, 1=True
	TargetPopulation Age_4164	Age 50-64	Num	0=False, 1=True
	TargetPopulation Age_65	Age over 65	Num	0=False, 1=True
	TargetPopulation Age_adult	Adult, not specified age	Num	0=False, 1=True
	TargetPopulation Age_unknown	Not specified	Num	0=False, 1=True
	TargetPopulation Age_specifyage	Specify age/age range	Txt	
	TargetPopulation Country	Country/Region	Txt	
	TargetPopulation Other	Other characteristics of the population (e.g. co-morbidities)	Txt	
**Intervention**				
	Intervention Paragraph	The specific drug, device or other health care intervention	Txt	
**Comparator**				
	Comparator Paragraph **(Deleted on articles published after 2012)**	An alternative that the intervention is compared to	Txt	
	ComparatorID	Type of comparator	Num	1=Placebo, 2=Standard care, 3=None, 4=Other
	Comparator StandardCareText	Description of standard/usual care, if comparator is a standard care	Txt	
	Comparator OtherText	Other description of comparator	Txt	
**Cost/QALY from article**				
	$/QALY from Article	Incremental cost-effectiveness ratio from article (ΔCosts/ΔQALYs)	Num	Positive value
**Ratio calculation**				
**Per Person**				
	ComparatorDirectCostsPerPerson	Direct medical costs of comparator	Num	
	InterventionDirectCostsPerPerson	Direct medical costs of intervention	Num	
	DirectCostsPerPerson	(Direct medical costs of intervention)-(Direct medical costs of comparator)	Num	
	ComparatorNonHealthCostsPerPerson	Non-health care costs of comparator	Num	
	InterventionNonHealthCostsPerPerson	Non-health care costs of intervention	Num	
	NonHealthCostsPerPerson	(Non-health care costs of intervention)-(Non-health care costs of comparator)	Num	
	ComparatorTotalCostsPerPerson	Total costs of comparator	Num	
	InterventionTotalCostsPerPerson	Total costs of intervention	Num	
	TotalCostsPerPerson	(Total costs of intervention)-(Total costs of comparator)	Num	
	ComparatorQALYsPerPerson	QALYs of comparator	Num	
	InterventionQALYsPerPerson	QALYs of intervention	Num	

	QALYsPerPerson	(QALYs of intervention)-(QALYs of comparator)	Num	
**Cost/QALY from article quadrant**				
	$/QALY Quadrant	This refers to the net impact in costs and QALYs of the intervention, as represented in the cost-effectiveness ratio.	Num	1=Quadrant I, 2=Quadrant II, 3=Quadrant III, 4=Quadrant IV, 5=Could not determine
**Extended Dominance**				
	ExtendedDominance	Is the ratio/intervention eliminated due to extended dominance	Num	0=No 1=Yes
**Comments**				
	RatioComments	General comments on ratios	Txt	
**Cost/QALY from reader**				
	$/QALY from Reader	Re-calculated by reader, based on the costs and QALYs value reported in the article	Num	

Weights form				
**Not reported weight**				
	NoWeight	Paper does not report any weight values	Num	0=False, 1=True
**Health State**				
	HealthState	The utility weight for the specific health state/condition	Txt	
**Base Case Weight**				
	Weight	The utility weight value for the base case analysis	Num	
**Data source of the weight**				
	WeightDataFromPrimarySource	Data is derived from author's collection of utility weights generated specifically for this study	Num	0=False, 1=True
	WeightDataFromSecondarySource	Data is derived from another source other than this study	Num	0=False, 1=True
	WeightDataFromNoneNS	Not stated or could not determine	Num	0=False, 1=True
**Reference (if datasource = "Secondary")**				
	PreviousStudyCite	Record the reference number from article for studies not present in PubMed	Txt	
**Comments**				
	WeightComments	General comments on weights	Txt	

III. GLOSSARY

DISEASE CLASSIFICATION INFORMATION

STUDY THEME

The primary study topic, which we classify into six categories: (i) public health, (ii) men, (iii) women, (iv) children, (v) elderly and (vi) none of these.

DISEASE

Primary Disease: Each paper can be coded as only one primary disease. There are 23 major disease categories including (i) Infectious, (ii) Malignant Neoplams, (iii) Endocrine Disorders, (iv) Neuro-Psychiatric and Neurological Conditions, (v) Sense Organ Diseases, (vi) Cardiovascular Diseases, (vii) Respiratory Diseases, (viii) Digestive Diseases, (ix) Genito-Urinary Diseases, (x) Musculoskeletal and Rheumatologic Diseases, (xi) Maternal and Child Health (Perinatal), (xii) Congenital Anomalies, (xiii) Nutritional Deficiencies, (xiv) Anemias, (xv) Skin Diseases (Non-Cancer), (xvi) Dental/Oral Conditions, (xvii) Injuries/Exposures, (xviii) Critical Care, (xix) Allergy/Immunology, (xx) Hematology – Other, (xxi) Program/Organizational Interventions, (xxii) Patient-Centeredness, (xxiii) Other and (xxiv) None of the Above/Not Applicable.

ID number (DID- #)	Disease statement	Major disease category
1	Infectious	1
2	Malignant Neoplams	2
3	Endocrine Disorders	3
4	Neuro-Psychiatric and Neurological Conditions	4
5	Sense Organ Diseases	5
6	Cardiovascular Diseases	6
7	Respiratory Diseases	7
8	Digestive Diseases	8
9	Genito-Urinary Diseases	9
10	Musculoskeletal and Rheumatologic Diseases	10
11	Maternal and Child Health (Perinatal)	11
12	Congenital Anomalies	12
13	Nutritional Deficiencies	13
14	Anemia’s	14
15	Skin Diseases (Non-Cancer)	15
16	Dental/Oral Conditions	16
17	Injuries/Exposures	17
18	Critical Care	18
19	Allergy/Immunology	19
20	Hematology - Other	20
21	Program/Organizational Interventions	21
22	Patient-Centeredness	22
23	Other	23
24	Other Secondary	24

Secondary Disease: Each paper can be coded as can be multiple choices for secondary disease. There are 71 disease statements in total. (See the table on subsequent page #26)

ID number (DID- #)	Disease statement	Major disease category
111	STDs excluding HIV	1
112	HIV/AIDS	1
113	Respiratory Infections	1
114	Otitis Media	1
115	Other Infectious Diseases	1
120	Colorectal Cancer	2
121	Lung Cancer	2
122	Breast Cancer	2
123	Cervical Cancer	2
124	Uterine Cancer	2
125	Ovary Cancer	2
126	Prostate Cancer	2
127	Hematologic Cancers (Lymphomas, Leukemia)	2
128	Other Neoplasm’s	2
130	Diabetes Mellitus	3
131	Other Endocrine	3
140	Depression and Bipolar Affective Disorder	4
141	Schizophrenia	4
142	Seizure Disorders (Epilepsy)	4
143	Alzheimer's and Other Dementias	4
144	Parkinson’s Disease	4
145	Multiple Sclerosis	4
146	Substance Abuse Disorders	4
147	Other Neuro-Psychiatric and Neurological Disorders	4
150	Vision	5
151	Hearing	5
160	Ischemic Heart Disease	6
161	Non-Ischemic Heart Disease - Other	6
162	Cerebro-vascular Disease	6
163	Hypertension	6
164	Lipids	6
165	Vascular, Non-Cardiac, Non-Cerebral	6
170	COPD	7
171	Asthma	7
172	Other Respiratory	7
180	Peptic Ulcer Disease	8
181	Cirrhosis of the Liver	8
182	Other Non-Infectious GI Diseases	8
190	Kidney Disease	9
191	Non-Cancer Prostate Disease	9
192	Other Genito-Urinary	9
200	Rheumatoid Arthritis	10
201	Osteoarthritis	10
202	Other Musculoskeletal	10
203	Other Rheumatologic	10
255	None of the Above/Not Applicable	255

Method Information

The CEA Registry will now include limited information for cost per life year gained studies.

Select the check boxes for the type of study. For studies reporting cost per QALY and cost per LY make sure to check both boxes.

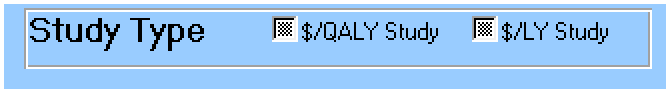

1. Intervention types

This reflects the primary intervention that was evaluated.

Care Delivery: Provision of care; development of facility or distribution of personnel (e.g. a policy that changes the nurse-to-patient ratio, patient self-management program)

Health Education or Behavior: An intervention designed to educate individuals on behaviors that promote, maintain or restore health (e.g. smoking cessation and prevention program)

Pharmaceutical: Any drug or biotech product used for medical treatment or prevention (e.g. Lovastatin, Herceptin)

Surgical: Invasive; surgery/cutting involved (e.g. transplantation, appendectomy although bone marrow transplantation would be a medical procedure)

Immunization: Receipt of vaccination (e.g. flu vaccine, HPV vaccine)

Diagnostic: A method used to determine if and what type of disease is present (e.g. imaging, biopsy, PET scan, x-rays, in-vitro testing)

Medical Procedure: Non-surgical, non-diagnostic procedures (e.g. angiogram, blood donation, mole removal, casting)

Medical Device: May or may not require a surgical or implantation procedure (e.g. pacemaker, insulin pump, leg brace and crutches)

Screening: Refers to measures that detect disease (or test for risk factors) before it is symptomatic (e.g. breast cancer screening – mammogram)

Other: Any intervention not described above (e.g. injury prevention, food safety, or environmental health)

2. Prevention Stage

Prevention stage definitions (*Ref: Concise Medical Dictionary. Oxford University Press, 2007. Oxford Reference Online. Oxford University Pres*).

Primary: [Interventions involved with the] avoidance of the onset of disease by behavior modification or treatment, such as immunization, promotion of safety equipment use (e.g. seat belts), health education (e.g. anti-smoking campaigns), promotion of improved nutrition and prenatal care.

Secondary: [Interventions involved with the] avoidance or alleviation of disease by early detection and appropriate management. Secondary prevention includes population screening to identify disease in asymptomatic people to enable timely treatment.

Tertiary: [Interventions involved with] treatment to reduce complications and progression of established disease, e.g. remedial exercises for contractures, care of pressure points and bladder function in paraplegia, cardiac rehabilitation following myocardial infarction, stroke rehabilitation, and screening of people with diabetes for diabetic retinopathy.

3. **Country of Study:** The geographical perspective of the economic evaluation. I.e. the country to which the study's results are applied. The perspective of the study relates to practice patterns and unit costs for medical care/interventions/other societal costs that are included in the economic evaluation. For example, if country is UK, then the CE ratio represents the expected costs and QALYs; if intervention were adopted in UK, irrespective of what country individual data (e.g. clinical trial data) are derived from.

4. **Primary Affiliation of Author(s):** Authors' primary association or membership during time of publication

   University/Academic Organization: Universities, institutes/centers or any other academic organizations; teaching hospitals and medical schools are included

   Government Organization: Institutes, centers or organizations that are part of the government (e.g. NIH, CDC, VA appointments etc.)

   Contract Res./Consultant: Contract researchers or consulting firms (e.g. IMS Health, UBC, i3 Innovus)

   Health Care Organization: Health care providers (e.g. insurance companies, hospital, HMOs)

   Pharm./Med Device Industry: Industrial pharmaceutical companies, biotech companies and medical device companies

   Not Stated/Unknown: No affiliation information provided in the article

   Other: Other than the choices above

5. **Study Sponsorship/Funding**

All funding sources that supported the study.

Government: See Q#4. Government Org.

Health Care Org: See Q#4. Health Care Org.

Foundation: A nonprofit, non-governmental organization (e.g. Robert Wood Johnson Foundation, PhRMA Foundation)

Professional Membership Org.: Professional membership organization, membership organizations of professional persons formed for the advancement of the interests of their profession (e.g. Academy Health, ISPOR)

Pharm./Med Device Co.: See Q#4. Pharm./Med Device Industry

Others: Other than the choices mentioned above

Not stated/Could be not determined: No information provided

None: Paper explicitly states no funding

REPORTING OF METHODS

6. Time horizon/s analyzed

The time horizon is the length of time by which resource use and health effects are measured, not the duration of data collection (see *Gold MR. et al.* *Cost-Effectiveness in Health and Medicine*).

A. Stated: Whether or not the paper clearly reported a time-horizon

B. What was the base case time horizon analyzed?

Lifetime: Whether or not study used lifetime analysis as base case

Other: Magnitude- Duration of units in prior question

Units: Days/Weeks/Years. This is the unites of the main/base-case that the analysis used. In the previous example of 5 years and lifetime, either example may take precedence. The exact determination of the main/base case is the responsibility of the study author to disclose. If no explicit disclosure is made, the main/base case horizon is the same horizon used for the main/base case results.

C. Multiple: If the paper conducted the same analysis from multiple time horizons (e.g. both 5 years analysis and lifetime analysis).

D. Lifetime: If the alternate case is a lifetime analysis.

Other time horizons/text: True is the alternate case is NOT lifetime; other/text reports duration.

7. **Perspective**

The viewpoint from which costs and QALYs were calculated. The perspective is reported by author and reviewer.

<u>Note</u>- If the author states 'societal' and only included costs pertaining to 'limited societal', in that case you will still consider that the perspective was identified correctly.

As Judged by Author: The perspective used in the study as stated by the author.

Note- Limited Societal is a term coined by the CEA Registry. If an author states the perspective to be societal then you need to review the costs included in the study and if they meet the inclusion criteria for limited societal then select 'Limited Societal' instead of 'Societal'. The difference between limited societal and societal is explained below.

As Judged by Reviewer: The perspective used in the study as judged by the reviewer. For example, a study might state 'societal perspective' but fail to include non-medical costs and other sector costs. In this case the perspective as judged by the author would be "limited societal" but as judged by the reviewer would be 'health care payer'.

Health Care Payer: Includes only monetary costs incurred by a (typically 3rd party) health care payer (e.g. Medicare/Medicaid, British National Health service, an HMO or private health care plan).

Health Care Sector: *Health Care Sector* perspective is similar to *Health Care Payer*. However, the *Health Care Sector* perspective accounts for all monetary costs of health care, regardless who bears the cost (includes out-of-pocket costs).

Please note a key distinction between the *Health Care Sector* and *Health Care Payer* perspectives is that the *Health Care Sector* perspective includes out-of-pocket costs and *Health Care Payer* does not.

Limited Societal: *Limited Societal* perspective analysis accounts for costs not unique or specific to the health care sector. The perspective is designated "limited societal" if at least one such cost is included (e.g. unpaid caregiver time, productivity impacts, patient time, patient out of pocket costs). It does not include spillover costs to other sectors besides health care, such as education.

Societal: The *Societal* perspective is broader than limited societal perspective. It represents the overall public interest by including social opportunity costs where the use of resources results in the loss of opportunity to use those funds (or resources) for other purposes. This goes beyond the *Limited Societal* perspective; in that the analysis accounts for cost impacts on at least one of the other sectors, such as environmental, educational, and judicial.

Note- If an author states '**Societal**' but does not state any spillover costs for other sector (education, housing, judicial) then perspective as judged by author and reader should be '**Limited Societal'**

Not Stated/Could Not Be Determined: Authors did not provide sufficient information to determine type of costs or benefits evaluated

Other: Authors report a perspective other than those listed above

8. <u>COSTS INCLUDED</u>

Health care costs- This category includes costs associated directly with the treatment. This includes direct medical costs and any other medical costs incurred by the patient (out of-pocket medical costs). E.g., vaccine costs, drug cost, physician visit cost, follow-up visit cost, hospitalization cost, outpatient cost.

Non-health care costs- This category includes costs not incurred by the health care sector (e.g., unpaid caregiver time, productivity impacts, patient time, income loss).

Patient time- patient time lost due to illness or travel

Caregiver time- costs associated with time and care provided by caregiver

Transportation- costs associated with travel to health care facility

Productivity gains- costs associated with on-job productivity losses or presenteeism. Presenteeism is illnesses and medical problems that undermine job performance in the workforce.

Income loss- Income lost due to lost days of work

Other- Other costs unrelated to treatment

Other Costs/Sectors- This includes-

Public health- Costs related to public health resources, e.g., shelters, public services, wellness programs, and food provisions.

Legal/criminal justice- Costs related to arrests, convictions, days in jail/prison.

Education- Costs related to paying for school and college.

Housing- Costs related to public and private housing, property value.

Environment- Costs related to environmental impact of the intervention.

Could not be determined- If the study does not provide sufficient information to determine the type of costs included.

DISCOUNTING

9. Were costs/QALYs discounted?

Money available or spent now is more valuable than money available or spent in the future because of opportunity costs. Discounting quantifies this time preference and places all economic costs in terms of the present value of money. When health effects can be valued in monetary terms over a period of time, then both health effects and associated costs can be discounted.

If the time horizon is less than one year, then no discount rate should be applied.

The interest rate used to compute present value of costs incurred and QALY accrued in order to compare costs and QALY. The present value ($QALY_{PV}$) of an incremental gain in quality adjusted life years ($\Delta QALY$) occurring n years in the future is calculated as

$$QALY_{PV} = \frac{\Delta QALY}{(1+i)^n} ,$$

where i is the annual discount rate. For interventions that affect QALY gains over more than a particular point in time, the present value can be calculated by summing over all affected points in time. In particular, for changes in QALY every year 't' over 'n' years in the future,

$$QALY_{PV} = \sum_{t=0}^{n} \frac{\Delta QALY_t}{(1+i)^t} ,$$

where t indicates the number of years in the future. The present value of a change in costs ($\Delta \cos t$) occurring each 't' years over 'n' years in the future can be calculated in an analogous manner,

$$Cost_{PV} = \sum_{t=0}^{n} \frac{\Delta \cos t_t}{(1+i)^t} .$$

COST MEASUREMENT

10. Currency of the CE ratio numbers

The currency the authors adopted or converted to in the study. If the currency and year are not given, we assume the currency used in the analysis is denominated in currency of the country in which the analysis represents and valued 2 years prior to article publication. We obtain inflation values from the US Bureau of Labor Statistics and currency conversations from six month averages of over the counter currency prices.

REPORTING OF RESULTS

11. "Cost-effectiveness" threshold

The incremental cost-effectiveness ratio (ICER) that the study authors used to assess whether the intervention is cost-effective.

20K US$; 50K US$; 100K US$: Represents $20,000; $50,000 and $100,000 in US dollars.

Laupacis: Refers to a landmark article in the field (see *Laupacis A, Feeny D, Detsky AS, Tugwell PX. How attractive does a new technology have to be to warrant adoption and utilization? Tentative guidelines for using clinical and economic evaluations revisited. Canadian Medical Association Journal 1992;146:473-81*)

Range: A range in US$ is provided as the threshold, reported with **RangeLower** and **RangeUpper**

Other: A US$ amount that is not **20K US$, 50K US$ or 100K US$;** Reported as **OtherText**

Other Non-US: Any range or value that is given in a currency other than US$ (e.g. £20K, £30K, €50K)

None: No cutoff specified.

DISCUSSION SECTION

12. Overall quality of the analysis

A subjective rating score by reviewers from 1 (lowest quality) to 7 (highest quality). Scores should reflect the following considerations (in rough order of importance):
1) Whether incremental cost-effectiveness ratios are correctly computed,
2) Comprehensive characterization of uncertainty (i.e., either probabilistic or non-probabilistic evaluation of how changes in plausibly important assumptions influence the results,
3) Correct treatment and explicit specification of health economic assumptions (discount rate, currency, analysis time horizon,
4) Appropriate and explicit estimation of utility weights (the importance of this item depends on the extent to which the intervention influences morbidity *vs.* mortality.

An average score of both the reviewers is entered for the **Consensus copy**.

13. Comments

Any general, notable comments regarding the methods of analysis employed, or information not captured elsewhere in this form.

Ratio Information

A ratio is specified by three characteristics: (i) the target population (item #20), (ii) the intervention (item #21), and (iii) the comparator (item #22). In general, that means no two ratios reported for the same paper should have identical entries for all three of these characteristics. If two values are reported for the same ratio (i.e. the same three elements), the two ratios differ because of an alternative assumption (e.g. the use of a societal perspective, rather than a health care system perspective).

Studies reporting cost per life year gained

- If a study reports only cost per life year gained, do not report disaggregate costs or life year values in Q5. Only complete the target population, intervention, and comparator fields (Q1-3).
- If a study reports both then only report cost per QALY values. Be sure to indicate that both were reported on methods form. On the ratios form only report the information for cost per QALY ratio/s.

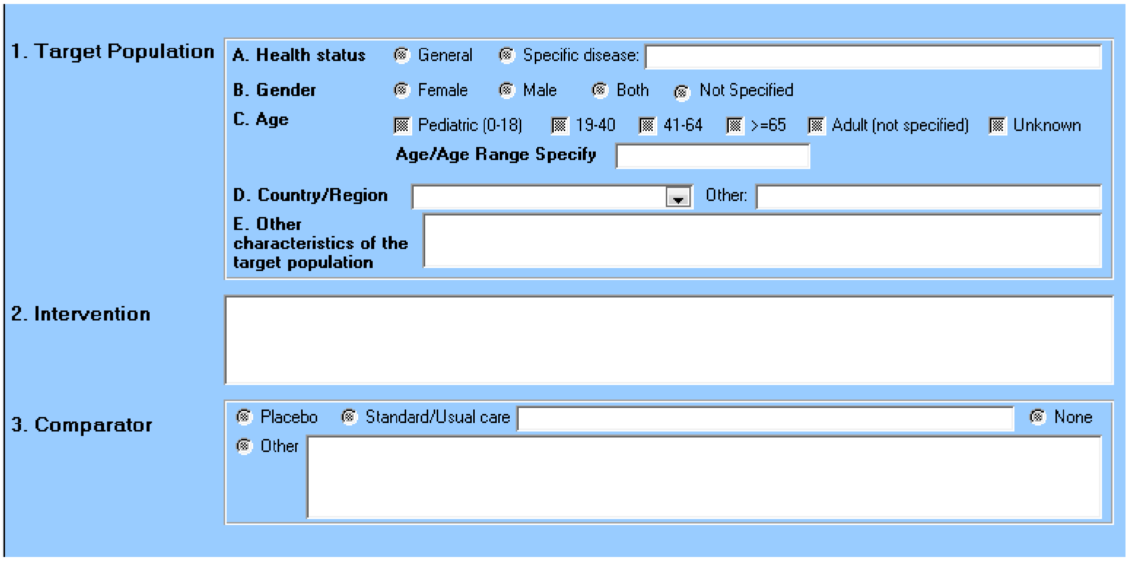
1. Target Population
A. Health status ○ General ○ Specific disease:
B. Gender ○ Female ○ Male ○ Both ○ Not Specified
C. Age ☐ Pediatric (0-18) ☐ 19-40 ☐ 41-64 ☐ >=65 ☐ Adult (not specified) ☐ Unknown
Age/Age Range Specify
D. Country/Region Other:
E. Other characteristics of the target population
2. Intervention
3. Comparator
○ Placebo ○ Standard/Usual care ○ None
○ Other

1. **Target Population**

   A. Health status of the target population, coded either as a general health or a specific comorbidity. General health can be stated as healthy (text box), if the study looks at healthy population.

   B. Gender: If the intervention is for a specific gender- male/female/both sex. Not specified: If male or female gender is not specified in the study.

   C. Age 0-18: if the targeted population included those less than or equal to 18 y.o.
   Age 19-40: if the targeted population included those between 19 and 40 y.o.
   Age 40-64: if the targeted population included those between 40 and 64 y.o.
   Age 65: if the target population included those greater than 65 y.o.
   Age adult: if the target population included non-specific adult (>18 y.o.) population
   Age unknown: If age of the target population is not stated in the sudy
   Age/Age Range, specify: specify the ages/age range used of the target population

   D. Country: Country of the target population, entered even if this population is the same country as recorded within the article information.

   E. Other characteristics: The recipients of the primary intervention and comparator. Include other characteristics (e.g. co-morbidities) not included in text box A (Specific disease field in health status).

2. **Intervention**

   The primary technology, procedure, or health services evaluated in the study. **Do not use abbreviations to describe the intervention.**

3. **Comparator**

   The alternative to which the intervention is compared. Common comparators are "usual care"/ "standard of care" or placebo or none (doing nothing). **Do not use abbreviations to describe the intervention.**

   Comparator_OtherText- Include other active comparators as described in the study

Readers are encouraged to not make new ratio forms for different perspectives, different dosage strategies, different time-horizons, or different discount rates. These alternate scenarios should be noted in Q15 under Uncertainty Analyses.

For multiple perspectives, always report the broader or all comprising perspective. For e.g. limited societal instead of healthcare payer.

4. $/QALY from article

Only report cost per DALY value stated in the study. Do not report 0 or negative values.

The incremental cost-effectiveness ratio (ICER) reported in the original article: ${}^{D Costs}/_{D QALYs}$.

This is a numeric field that must be non-negative.

5. Table of cost and QALY values

Note: Make sure to convert to per patient values for results reporting entire population values.

Per patient values	**Per Person**		
	Intervention	Comparator	Difference
Direct Medical Costs			
Non-Health Care Costs			
Total Costs			
QALYs Averted			
$/QALYs Gained			Auto-Calculated

Cost/QALY from reader:
Automatically Recalculated value based on the incremental costs and QALYs from within the manuscript and identified by the reader.

For calculating or reporting ICERs from sequential analysis refer to the Appendix.

6. Ratio Quadrant- $/QALY Gained Quadrant

The quadrant describes where the ratio is located in the cost-effectiveness plane (see figure, below).
A ratio lies in <u>QUADRANT I</u> (the northwest quadrant) if the intervention increases costs but does not improve health. Ratios in QUADRANT I are sometimes referred to as "dominated".
A ratio lies in <u>QUADRANT II</u> (the northeast quadrant) if the intervention increases costs and improves health.
A ratio lies in <u>QUADRANT III</u> (the southwest quadrant) if the intervention saves money and does not improve health.
Finally, a ratio lies in <u>QUADRANT IV</u> (the southeast quadrant) if it both improves health and saves money. Ratios in QUADRANT IV are sometimes referred to as "dominant".

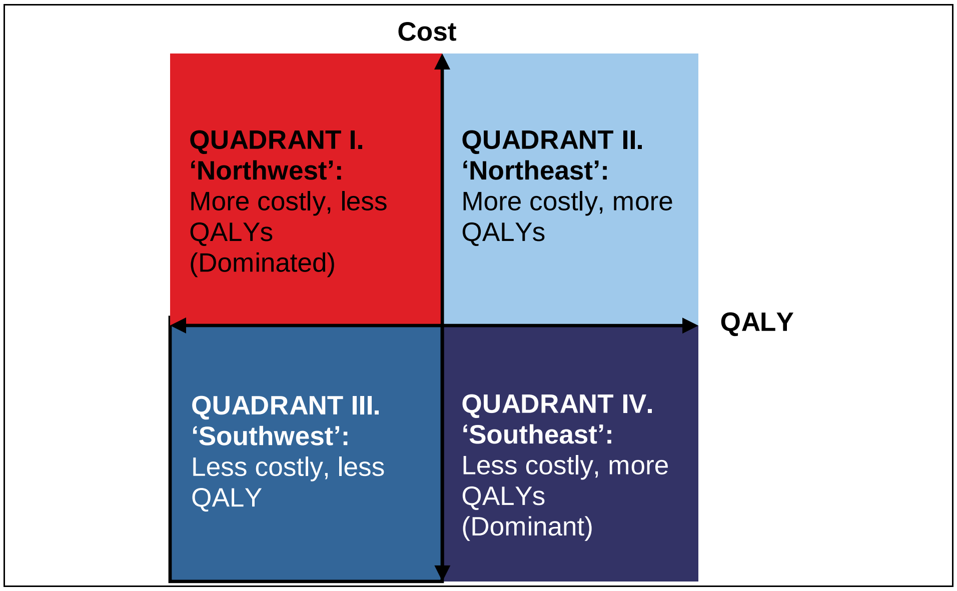

Figure 3: Four Quadrants of Cost-Effectiveness Plane

7. Extended Dominance

State whether the reported ratios/interventions is eliminated by extended dominance.

Table 2. Costs, Quality-Adjusted Life Expectancy, and Cost-effectiveness of Treatment for HCV*

Treatment Strategy†	Costs, $	Undiscounted Life Expectancy, mo	Discounted Life Expectancy, mo	C/E Ratio, $/YLS‡	QALM	C/E Ratio, $/QALY‡
**Coinfected Patients With CD4 Cell Counts of 350 Cells/μL and Moderate Chronic HCV, Genotype 1**						
No treatment	139 000	141.79	116.68	. . .	83.78	. . .
Interferon alfa, 48 wk	141 600	143.34	117.75	Dominated‖	85.32	Dominated‖
Interferon alfa and ribavirin, 24 wk	143 700	144.95	118.82	Dominated‖	87.12	Dominated‖
Interferon alfa and ribavirin, 48 wk	144 900	144.63	120.51	18 500	89.93	11 600
Pegylated interferon alfa, 48 wk	145 000	147.52	118.60	Dominated§	86.71	Dominated§
Pegylated interferon alfa and ribavirin, 48 wk	150 400	149.03	121.52	65 100	91.57	40 000

‖This strategy is weakly dominated (ie, eliminated by extended dominance) because it is less effective and is associated with a less attractive C/E ratio than an available alternative strategy.

In the example above create ratio forms for 5 interventions. For additional information refer appendix 1. While reporting comparators make sure to exclude the interventions that were dominated or showed extended dominance. For the ratios that report <u>extended dominance</u>, make sure you select 'Yes' for Q7.

8. Comments:

Any comments the reader found noteworthy during the review of the ratio(s).

UTILITY WEIGHTS INFORMATION

WEIGHT

All the information entered is specific to each weight. If a study reports multiple weights; the reference information, measurement scale, elicitation method and so on may vary for each weight. In that case make each weight form specific to every weight reported.

1. **Health state considered:**

   The utility weight for the specific health state/condition. Be concise and specific regarding the disease the utility is associated with.

2. **Base case utility:**

   The utility weight value for the base case analysis. This is always **reported as a utility** and not as a disutility* (see Q#5-Decrement, below).

   *Note: Reporting disutilities:
   In instances in which a disutility is provided, we assume a baseline of "1", and subtract the absolute value of the disutility rather than putting in a negative disutility value. E.g. if the disutility for blindness is 0.2, then compute the utility as 1- 0.2=0.8. **Do not assume the base case weight to be 1, if not reported in the study.** Please make sure to report in the "Comments" section (Q#45) that the weight was reported as a disutility and your calculation, what the disutility was, and that the disutility was subtracted from 1 to get the weight value.

   Note- Do not report utility values of 1 (perfect health) and 0 (death)

3. **Data Source**

   Primary data: Data is derived from author's collection of utility weights generated specifically for this study.
   Secondary data: Data is derived from another source other than this study.

4. **If secondary data are used where are they from?**

   Record the reference number(s) or the parenthetical citation separated by commas.

5. **Comments Notes:**

   Any comments the reader found noteworthy during the review of the utility weight(s).

APPENDIX 1. SEQUENTIAL ANALYSIS

Consider the following data for six interventions (from Table 2 in Kuehne et al., 2002)

	Intervention	Cost	QALM	ΔC	ΔQALM	CE
1	No Treatment	139000	83.78			
2	Interferon α, 48 wk	141600	85.32	2600	1.54	$20,260
3	Interferon α and ribavirin, 24 wk	143700	87.12	4700	3.34	$16,886
4	Interferon α and ribavirin, 48 wk	144900	89.93	5900	6.15	$11,512
5	Pegylated interferon α, 48 wk	145000	86.71	100	-3.22	-$373
6	Pegylated interferon α and ribavirin, 48 wk	150400	91.57	5500	1.64	$40,244

Note: "QALM" is quality adjusted life month.

Each intervention's cost and QALYs can be plotted, with cost on the horizontal axis and effect on the vertical axis. The incremental cost-effectiveness of one intervention compared to another is the inverse of the slope connecting the two points for those interventions. Hence, steep slopes are favorable because their inverse is small – *i.e.*, the cost-effectiveness is lower.

For the results in the table above, interventions (2) and (3) are said to be "weakly dominated" or "eliminated by extended dominance" because they can be bypassed in favor of intervention (4). Intervention (4) costs more than either (2) or (3), but its incremental cost-effectiveness relative to (1) is superior to the incremental cost-effectiveness of either (2) or (3). Intervention (5) is "strongly dominated" because it is associated with higher costs and less health than (4). The last intervention is (6), which is more expensive than (4), but confers greater health benefits.

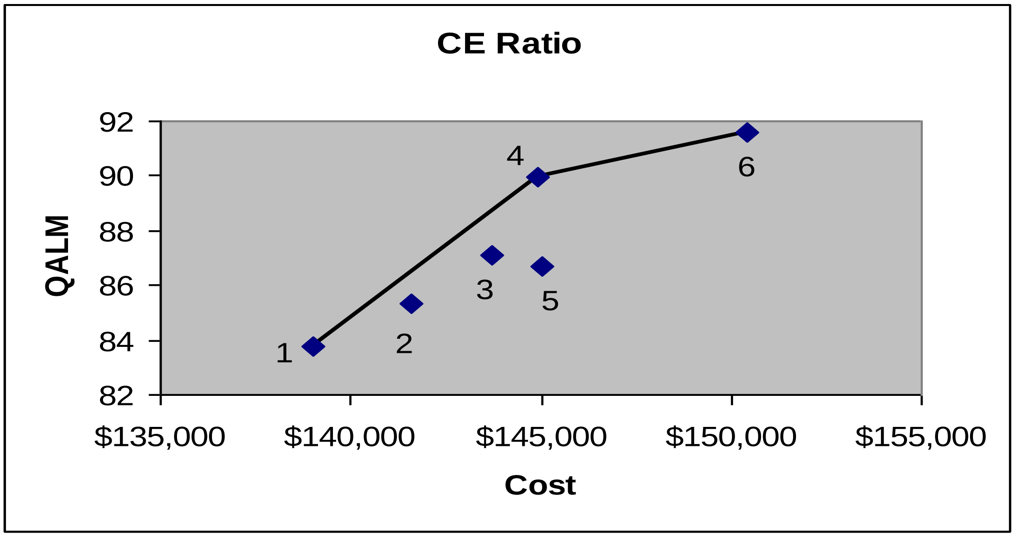

Papers conducting sequential analyses often do not report the incremental cost-effectiveness of strategies that are either weekly or strongly dominated. In this example, intervention (2) would have been reported if it were not for the case that it was weakly dominated by a strategy that is compared to (1) "later" in the analysis.

These rules can be thought of graphically. After plotting all of the data points corresponding to the interventions, an efficient frontier can be identified. This frontier is the set of straight lines furthest to the "north west". Any interventions that are to the southeast of this frontier are omitted from further consideration.

The table below is from the original Kuehne *et al.* article. Note that cost-effectiveness ratios are reported for all interventions (5 ratios), even the ones reporting extended dominance. Those ratios will be correctly indicated in Q7

Table 2. Costs, Quality-Adjusted Life Expectancy, and Cost-effectiveness of Treatment for HCV*

Treatment Strategy†	Costs, $	Undiscounted Life Expectancy, mo	Discounted Life Expectancy, mo	C/E Ratio, $/YLS‡	QALM	C/E Ratio, $/QALY‡
**Coinfected Patients With CD4 Cell Counts of 350 Cells/µL and Moderate Chronic HCV, Genotype 1**						
No treatment	139 000	141.79	116.68	. . .	83.78	. . .
Interferon alfa, 48 wk	141 600	143.34	117.75	Dominated‖	85.32	Dominated‖
Interferon alfa and ribavirin, 24 wk	143 700	144.95	118.82	Dominated‖	87.12	Dominated‖
Interferon alfa and ribavirin, 48 wk	144 900	144.63	120.51	18 500	89.93	11 600
Pegylated interferon alfa, 48 wk	145 000	147.52	118.60	Dominated§	86.71	Dominated§
Pegylated interferon alfa and ribavirin, 48 wk	150 400	149.03	121.52	65 100	91.57	40 000

IV. PUBLICATIONS

2013

Richards D. Only limited evidence available for the effectiveness and cost effectiveness of dental auxiliaries. Evid Based Dent. 2013;14(2):38-9.

Tetteh E, Morris S. Systematic review of drug administration costs and implications for biopharmaceutical manufacturing. Appl Health Econ Health Policy. 2013 Oct;11(5):445-56.

Tang DH, Harrington AR, Lee JK, Lin M, Armstrong EP. A systematic review of economic studies on biological agents used to treat Crohn's disease. Inflamm Bowel Dis. 2013 Nov;19(12):2673-94.

Olchanski N, Cohen JT, Neumann PJ. A role for research: an observation on preventive services for women. Am J Prev Med. 2013 Jan;44(1 Suppl 1):S12-5.

2012

Chokshi DA and Farley TA. The Cost-Effectiveness of Environmental Approaches to Disease Prevention. NEJM. 2012(367)2950297.

Otero HJ, Fang CH, Sekar M, Ward RJ, Neumann PJ. Accuracy, Risk and the Intrinsic Value of Diagnostic Imaging: A Review of the Cost-utility Literature. Acad Radiol. 2012 Feb 18.

Wilson AW, Neumann PJ. The cost-effectiveness of biopharmaceuticals: A look at the evidence.

Simoens S. What is the value for money of medicines? A registry study. J Clin Pharm Ther. 2012 Apr;37(2):182-6.

Finnell SM, Carroll AE, Downs SM. Application of classic utilities to published pediatric cost-utility studies. Acad Pediatr. 2012 May;12(3):219-28.

Miller G, Cohen JT, Roehrig C. Cost-effectiveness of cardiovascular disease spending. J Am Coll Cardiol. 2012 Nov 13;60(20):2123-4.

Heijnsdijk EAM, Wever EM, Auvinen A, et al. Quality-of-life effects of prostate-specific antigen screening. N Engl J Med 2012; 367:595-605.

2011

Fang C, Otero HJ, Greenberg D, Neumann PJ. Cost-utility analyses of diagnostic laboratory tests: a systematic review. Value Health. 2011 Dec;14(8):1010-8.

Kamae MS, Kamae I, Cohen JT, Neumann PJ. Regression analysis on the variation in efficiency frontiers for prevention stage of HIV/AIDS. Journal of Medical Economics 2011. Feb 18;14(2)187-93.

Greenberg, D and Neumann, PJ. Does adjusting for health-related quality of life matter in economic evaluations of cancer-related interventions? Expert Rev. Pharmacoeconomics Outcomes Res. 11(1),

113-119.

Braithwaite RS and Mentor SM. Identifying Favorable-Value Cardiovascular Health Services. American Journal of Managed Care. 2011;17(6):431-438.

2010

Otero HJ, Rybicki FJ, Greenberg D, Mitsouras D, Mendoza JA, Neumann PJ. Cost-Effective Diagnostic Cardiovascular Imaging: When Does It Provide Good Value for the Money? International Journal of Cardiovascular Imaging 2010. Aug;26(6):605-12.

Chambers JD, Neumann PJ, Buxton MJ. Does Medicare Have an Implicit Cost-Effectiveness Threshold? Medical Decision Making 2010 Jul-Aug;30(4):E14-27.

Meckley LM, Greenberg D, Cohen JT, Neumann PJ. The adoption of cost-effectiveness acceptability curves in cost-utility analyses. Medical Decision Making 2010 May-Jun;30(3):314-9.

Neumann PJ, Auerbach HR, Cohen JT, Greenberg D. “Low-value” services in value-based insurance design. American Journal of Managed Care 2010;16(4):280-286.

Greenberg D, Rosen AB, Wacht O, Palmer J, Neumann PJ. A Bibliometric Review of Cost-Effectiveness Analyses in the Economic and Medical Literature: 1976-2006. Med Decis Makin. 2010 Mar 12.

Greenberg D, Earle CC, Fang CH, Eldar-Lissai A, Neumann PJ. When is cancer care cost-effective? A systematic overview of cost-utility studies in oncology. Journal of the National Cancer Institute 2010; 102(2):82-88.

2009

Meckley LM, Greenberg D, Cohen JT, Neumann PJ. The adoption of cost-effectiveness acceptability curves in cost-utility analyses. Medical Decision Making. Published online.

Rosen AB, Spaulding A, Greenberg D, Palmer JA, Neumann PJ. Patient adherence: A blind spot in cost-effectiveness analysis? American Journal of Managed Care. Am J Manag Care. 2009 Sep;15(9):626-32.

Neumann PJ. Costing and perspective in published cost-effectiveness analyses. Medical Care. 2009 July;47(7s1):s28-s32.

Neumann PJ, Fang CH, Cohen JT, 30 years of pharmaceutical cost-utility analyses: growth, diversity, and methodological improvement. Pharmacoeconomics. In press.

2008

Cohen JT, Neumann PJ, Weinstein MC. Dose prevention care save money? Health economics and the presidential candidates. *N Engl J Med*. 2008 Feb(7);358:661-663.

Neumann PJ, Jacobson PD, Palmer JA. Measuring the value of public health systems: The disconnect between health economists and public health practioners. American Journal of Public Health. 2008 Dec;98(12):2173-2180.

Otero HJ, Rybicki FJ, Greenberg D, Neumann PJ. 20 years of cost-effectiveness analysis in medical imaging: Are we improving?. Radiology. 2008 Dec;249(3):917-925.

2007

Brauer C, Neumann PJ, Rosen AB. Trends in cost effectiveness analysis in orthopedic surgery. *Clin Ortho Rel Res*. 2007 Apr;457:42-8.

Ladapo JA, Neumann PJ, Keren R, Prosser LA. Valuing children's health: a comparison of cost-utility analyses for adult and paediatric health interventions in the US. Review Article. *Pharmacoeconomics*. 2007; 25(10):817-828.

2006

Bell CM, Urbach DR, Ray JG, Bayoumi A, Rosen AB, Greenberg D, Neumann PJ. Is everything in health care cost-effective? Bias in published cost-effectiveness studies. *BMJ* 2006;332:699-703.

Brauer C, Greenberg D, Rosen AB, Neumann PJ. Trends in the use of health utilities in published cost-utility analyses. *Value Health*. 2006;9(4):213-18.

Hahn RW, Kosec K, Neumann PJ, Wallsten S. What affects the quality of economic analysis for life-saving investments? AEI-Brookings. *Risk Anal*. 2006;26(3):641-55.

Neumann PJ, Lin PJ, Greenberg D, Berger M, Teutsch S, Mansley E, Rosen AB, Weinstein MC. Do drug formulary policies reflect evidence of value? *Am J Manag Care.* 2006 12(1):30-6.

Talmor D, Shapiro N, Greenberg D, Stone PW, Neumann PJ. When is critical care medicine cost-effective? A systematic review of the cost-effectiveness literature. *Crit Care Med.* 2006;34(11):2738-47.

2005

Brauer CA, Olchanski NV, Rosen AB, Neumann PJ. Cost-utility analyses in orthopedic surgery. *J Bone Joint Surg Am*. 2005;87(6):1253-9.

Neumann PJ. Using cost-effectiveness analysis to improve health care: opportunities and barriers. Oxford University Press. 2005.

Neumann PJ, Greenberg D, Olchanski NV, Stone PW, Rosen AB. Growth and quality of the cost-utility literature, 1976-2001. *Value Health*. 2005;8(1):3-9.

Neumann PJ, Rosen AB, Greenberg D, Olchanski NV, Pande R, Chapman RH, Stone PW, Ondategui-Parra S, Nadai J, Siegel JE, Weinstein MC. Can we better prioritize resources for cost-utility research? *Med Decis Making*. 2005;25(4):429-36.

Neumann PJ, Rosen AB, Weinstein MC. Medicare and cost-effectiveness analysis. *N Engl J Med*. 2005;353:1516-22.

Rosen AB, Greenberg D, Stone PW, Olchanski NV, Neumann PJ. Quality of abstracts of papers reporting original cost-effectiveness analyses. *Med Decis Making*. 2005;25(4):424-28.

Stone PW, Schackman B, Neukermans CP, Olchanski N, Greenberg D, Rosen A, Neumann PJ. A synthesis of cost-utility analysis literature in infectious disease. *Lancet Infect Dis*. 2005; 383-91.

2004

Chapman RH, Berger M, Weinstein MC, Weeks JC, Goldie S, Neumann PJ. When does quality-adjusting life-years matter in cost-effectiveness analysis? *Health Econ*. 2004;13(5):429-36.

Greenberg D, Rosen AB, Olchanski NV, Stone PW, Nadai J, Neumann PJ. Delays in publication of cost utility analyses conducted alongside clinical trials: registry analysis. *BMJ*. 2004; 328:1536-1537.

Pirraglia PA, Rosen AB, Hermann RC, Olchanski NV, Neumann PJ. Cost utility analysis studies of depression management: a systematic review. *Am J Psychiatry*. 2004; 161(12):2155-62.

Schackman BR, Gold HT, Stone PW, Neumann PJ. How often do Sensitivity analyses for economic parameters change cost-utility analysis conclusions? *PharmacoEconomics* 2004;22(5):293-300.

2003

Winkelmayer WC, Cohen D, Berger M, Neumann PJ. Comparing cost-utility analyses in cardiovascular medicine. *Cardiovascular Health Care Economics*, Weintraub WS, editor. Totowa, NJ: Humana Press, 2003: 329-356.

2002

Neumann PJ, Levine BS. Do HEDIS measures reflect cost-effective practices? *Am J Pre Med*. 2002; 23(4):276-289.

2001

Bell CM, Chapman RH, Stone PW, Sandberg EA, Neumann PJ. An off-the-shelf help list: a comprehensive catalog of utility scores from published cost-utility analyses. *Med Decis Making*. 2001; 21:288-94.

2000

Chapman RH, Stone PW, Sandberg EA, Bell C, Neumann PJ. A comprehensive league table of cost-utility ratios and a sub-table of "panel-worthy" studies. *Med Decis Making.* 2000; 20:451-67.

Earle CC, Chapman RH, Baker CS, Bell CM, Stone PW, Sandberg EA, and Neumann PJ. Systematic overview of cost-utility assessments in oncology. *J Clin Oncol*. 2000; 18:3302-17.

Neumann PJ, Sandberg EA, Bell CM, Stone PW, Chapman RH. Are pharmaceuticals cost-effective? A review of the evidence. *Health Aff*. 2000; 19(2):92-109.

Neumann PJ, Stone PW, Chapman RH, Sandberg EA, Bell CM. The quality of reporting in published cost-utility analyses, 1976-1997. *Ann Intern Med.* 2000; 132(12):964-72.

Stone PW, Teutsch S, Chapman RH, Bell C, Neumann PJ. Cost-utility analyses of clinical prevention services 1976-1997. *Am J Pre Med.* 2000; 19:15-23.

Stone PW, Chapman RC, Sandberg EA, Liljas B, Neumann PJ. Measuring costs in cost-utility analyses: variations in the literature. *Int J Technol Assess Health Care.* 2000; 16(1):111-124.
