# Supplementary material for: Comparing the cost-per-QALYs gained and cost-per-DALYs averted literatures
Source: Gates Open Res. 2018 Mar 5;2:5. Originally published 2018 Jan 18. [Version 2] doi: 10.12688/gatesopenres.12786.2 (PMC5801595; doi:10.12688/gatesopenres.12786.2)
Supplement: Supplementary file 2 [file gatesopenres-2-13870-s0001.tgz › cea81abc-6a8f-4677-af16-b6e320b629de.pdf]

The Cost-Per Disability-Adjusted Life Year Analysis (Cost/DALY) Registry

User Guide

2014

Center for the Evaluation of Value and Risk in Health
Institute for Clinical Research & Health Policy Studies, Tufts Medical Center
800 Washington Street, #063
Boston, MA 02111

CONTENTS

I. BACKGROUND 3

ABOUT THE COST PER DALY REGISTRY 4

DATABASE STRUCTURE 5

SEARCH STRATEGY 8

II. DATA GUIDE 9

VARIABLE DICTIONARY 10

Methods Information 10
Ratio Information 15
Ratio calculation 15
Disutility/Disability Weights form 19

III. GLOSSARY 21

METHOD INFORMATION 22

RATIO INFORMATION 35

DISABILITY WEIGHTS INFORMATION 42

I. BACKGROUND

ABOUT THE COST PER DALY REGISTRY

The Center for the Evaluation of Value and Risk in Health at the Institute for Clinical Research and Health Policy Studies, Tufts Medical Center, Boston, MA is developing the Cost per DALY Registry. Development of this database, which will house information on Cost-per Disability Adjusted Life Year (DALY) studies, has been funded by the Bill and Melinda Gates Foundation.

Health-related CEAs estimate the resources used (costs) and the health benefits gained (effects) by an intervention compared to an alternative treatment strategy. The disability-adjusted life year, or DALY, is a summary measure of public health widely used to quantify burden of disease. People may lose healthy life years as a result of living with illness or by dying before they reach a reference life expectancy. The DALY is an aggregate measure that accounts for these losses. DALYs have become the global standard for measuring burden of disease, and DALYs have been the key measure in the four Global Burden of Disease (GBD) studies, each of which presented a comprehensive assessment of the worldwide health impact of disease, injury and risk factors.

The objectives of the Cost-per-DALY database are to help decision makers identify society’s best opportunities for targeting resources to improve health, to assist policymakers in healthcare resource allocation decisions, and to move the field towards the use of standard methodologies.

DATABASE STRUCTURE

Figure 1 illustrates the hierarchical structure of the cost per DALY Registry. A hierarchical structure is useful in cases where there is a one-to-many relationship between different data elements. The cost per DALY Registry has four main data sheets: the article sheet, ratio sheet, the disability weight sheet, and the variables sheet.

- Article sheet: contains one record for each article.
- Ratio sheet: contains one or more ratio records for each article.
- Disability/Disutility weights sheet: contains none or one or more disability weight records for each article.
- Variables Sheet: contains a listing and description of all included variables.

Figure 1 illustrates the Registry's contents for a single article that reports information for *N* ratios and *M* utility weights.

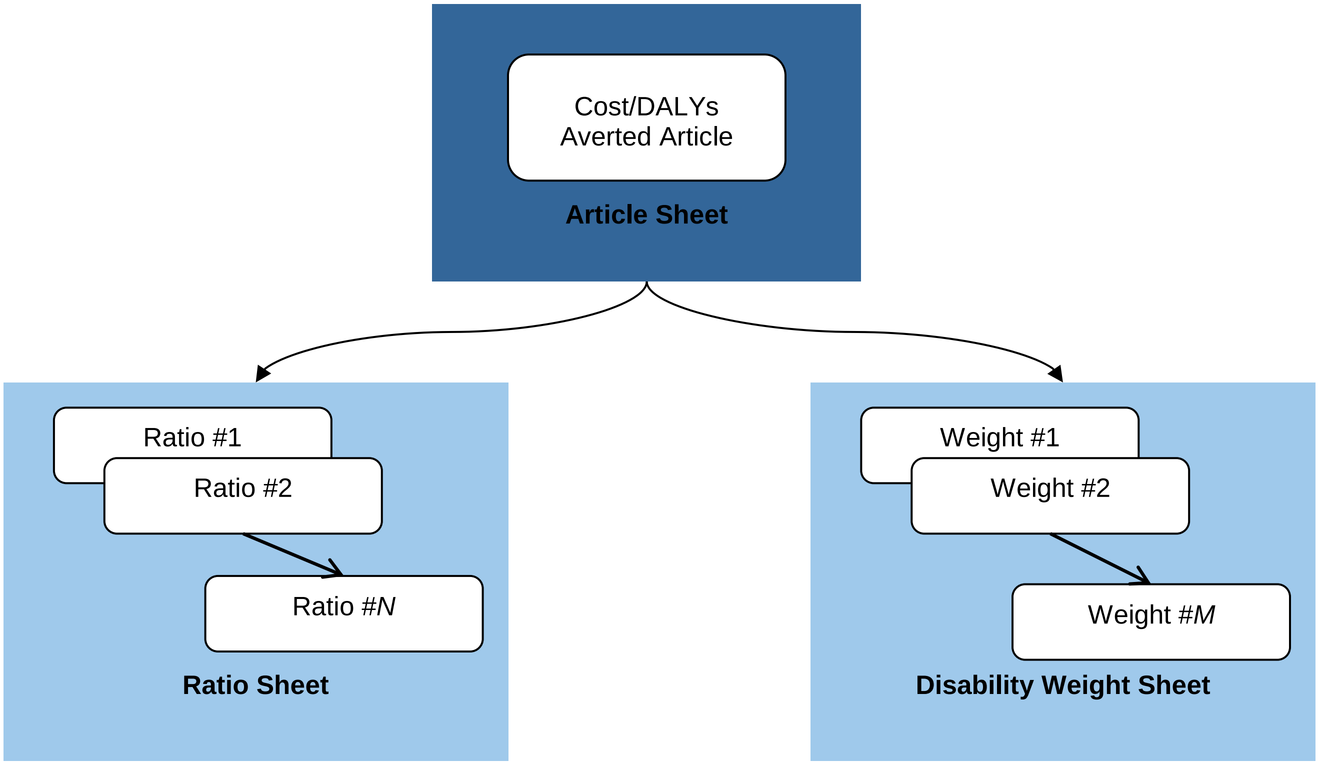

Figure 1 Cost per DALY Registry: Hierarchical Structure

METHODOLOGY

The published articles summarized in the Registry undergo a formalized review protocol (Figure 2). The analyses address a wide variety of diseases and treatments and all measure health effects in terms of disability-adjusted life years (DALYs). The disability-adjusted life year (DALY) is a measure of overall disease burden, expressed as the number of years lost due to ill health, disability or early death. The Registry team searches MEDLINE for English-language articles using keywords “DALYs”, and “disability-adjusted”. Abstracts from these articles are screened to determine if the paper contains an original cost-utility estimate. We exclude review, editorial, or methodological articles, as well as cost-effectiveness analyses that do not use DALYs to quantify health benefits.

Each article meeting these criteria is assigned a disease classification by a clinician. Two readers with training in decision analysis and cost-effectiveness analysis independently review each article and record information using a standardized set of forms and instructions. The two readers convene for a consensus audit to resolve any potential discrepancies. On occasion, a third reader may be called upon to help settle disputed items.

Data on over 40 variables are collected for each article.

ARTICLE INFORMATION

We report: 1) the type of intervention evaluated; 2) the country of the analysis; and 3) the funding source.

For methodology, we record 1) whether the article correctly calculated incremental cost-effectiveness ratios; 2) the analytic time horizon and analytic perspective (e.g., societal or health sector); 3) what discount rate, if any, was used; 3) the currency used; 4) types of costs included; 5) the type of sensitivity or uncertainty analysis used; 6) whether the article specified a threshold for identifying acceptably favorable cost-effectiveness ratios; 7) a subjective assessment regarding the article’s overall quality on an interval scale from 1 (low) to 7 (high); and 8) a subjective assessment regarding the the following three methodological components of the analysis: (i) the health economic methodology, (ii) the model’s and/or papers consideration and communication of uncertainty, (iii) the transparency of the model used in the analysis.

RATIO INFORMATION

We describe the health intervention that is the subject of the analysis, and comparator intervention to which it is compared, and the population that is eligible for the intervention. When available, we also report the costs and health benefits (DALYs averted) associated with both the target and comparator interventions. We report the value of the ratio reported in the original article, as well as the value we calculate directly from the cost and DALY information in the article. We also report the ratio quadrant (Northwest= more costly, less effective; Northeast= more costly, more effective; Southwest= less costly, less effective; and South east= less costly, more effective). (see figure #3, pg. #37).

DISABILITY WEIGHT INFORMATION

We report the health condition and demographics of the pertinent population (e.g. sex, age, and comorbidities), the disability weight or disutility value, and any reported range of plausible values. We report, when used, secondary literature sources relied upon to provide weight values.

SEARCH STRATEGY

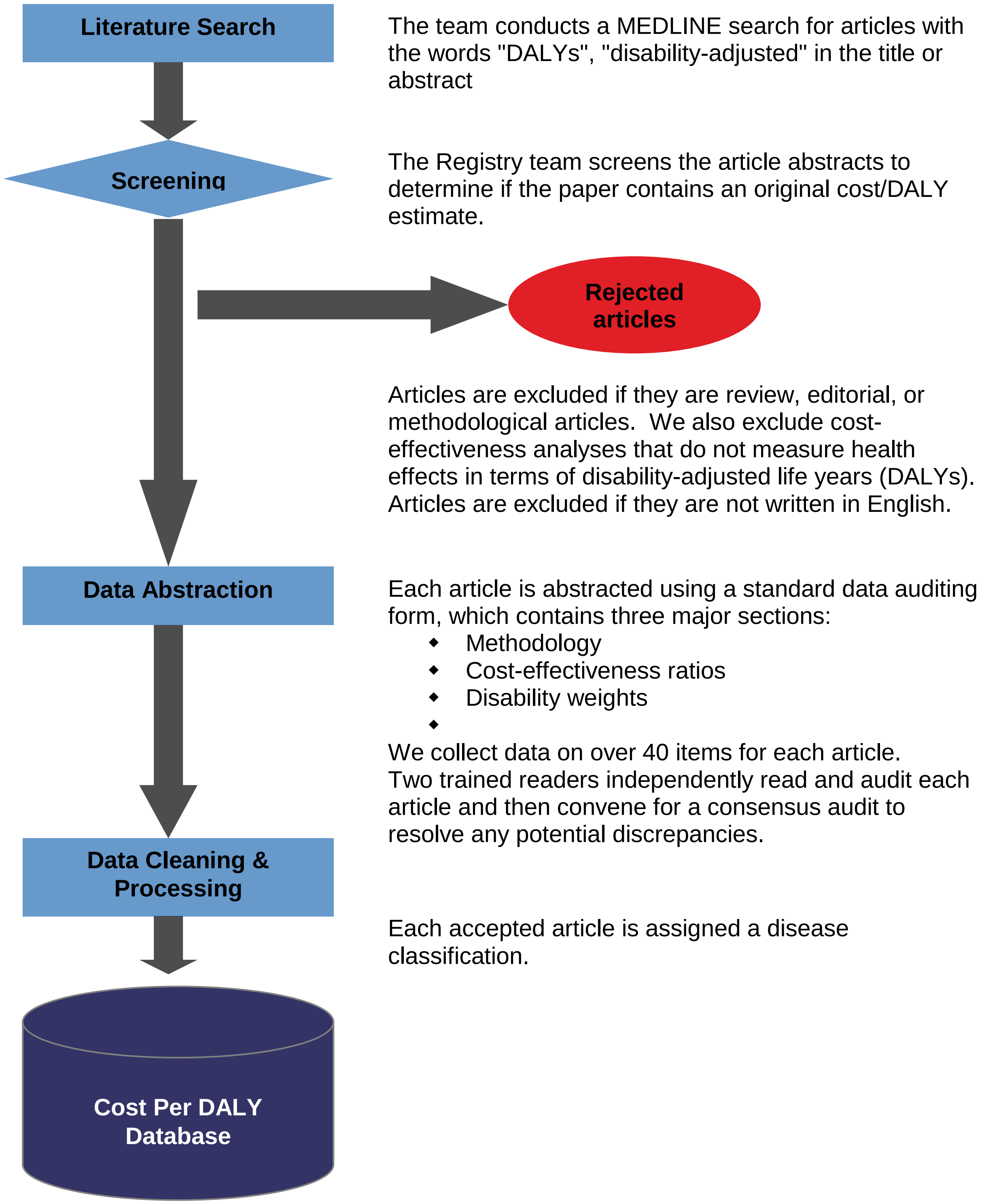

Figure 2: Search Strategy

II. DATA GUIDE

VARIABLE DICTIONARY

*Txt=Text, Num=Numeric

Methods Information				
**Author Email**				
	CorrespondingEmail	Primary author's email	Txt	
**Intervention Types**				
	Intervention CareDelivery	Development of facility or distribution of personnel	Num	0=False, -1=True
	Intervention Diagnostic	A method used to determine if and what type of disease is present	Num	0=False, -1=True
	Intervention HealthEducationOrBehavior	An intervention designed to educate individuals on behaviors that promote, maintain or restore health	Num	0=False, -1=True
	Intervention Immunization	Receipt of vaccination	Num	0=False, -1=True
	Intervention MedicalDevice	May or may not require a surgical or implantation procedure	Num	0=False, -1=True
	Intervention MedicalProcedure	Non-surgical, non-diagnostic procedures	Num	0=False, -1=True
	Intervention Pharmaceutical	Any drug or biotech product used for medical treatment or prevention	Num	0=False, -1=True
	Intervention Screening	Refers to measures that detect disease before it is symptomatic	Num	0=False, -1=True
	Intervention Surgical	Invasive; cutting involved	Num	0=False, -1=True
	Intervention Nutrition	Addressing malnutrition or poor diet through dietary changes, policy or education	Num	0=False, -1=True
	Intervention Maternal/NeonatalCare	Care received at antenatal visits, at delivery and for neonates	Num	0=False, -1=True
	Intervention Environmentalremediation	Removal of contamination from water bodies, soil or other areas	Num	0=False, -1=True
	Intervention Environmentalaugmentation	Provisions to inhibit or limit damage from environment	Num	0=False, -1=True
	Intervention Legislation	Laws and policy changes	Num	0=False, -1=True
	Intervention Other	Any intervention not described above	Num	0=False, -1=True
	Intervention OtherText	If other, text of intervention	Txt	
	Intervention NoneNA	None/Non-applicable	Num	0=False, -1=True
**Prevention Stage**				
	PreventionStage	Divided into 3 stages, primary, secondary and tertiary	Num	1=Primary, 2=Secondary, 3=Tertiary
**Scope of Study**				
	Scope Local	Targeted at a single and limited population	Num	0=False, -1=True
	Scope Province	Targeted at population within a particular province/s	Num	0=False, -1=True
	Scope National/Regional	Targeted at population within a particular country or countries	Num	0=False, -1=True
	Scope NotDetermined	Does not provide sufficient information	Num	0=False, -1=True
	StateLocality	If not national, state region/locality	Txt	
**Country of Study**				
	Country	1st country name	Txt	
	Country2	2nd country name	Txt	
	Country3	3rd country name	Txt	
	Country4	4th country name	Txt	

**Affiliation of Author(s)**				
	AuthorAffiliation Academic	Universities, institutes/centers or any other academic organizations	Num	0=False, -1=True
	AuthorAffiliation HealthCare	Health care organizations	Num	0=False, -1=True
	AuthorAffiliation Government	Governmental organizations	Num	0=False, -1=True
	AuthorAffiliation PharmOrDevice	Industrial pharmaceutical companies, biotech companies and medical device companies.	Num	0=False, -1=True
	AuthorAffiliation Consultant	Contract researcher/consultant	Num	0=False, -1=True
	AuthorAffiliation Other	Other than the choices above	Num	0=False, -1=True
	AuthorAffiliation OtherText	If other, name of author's affiliation	Txt	
	AuthorAffiliation NoneNS	No affiliation information provided in the article	Num	0=False, -1=True
**Study sponsorship/funding**				
	Sponsorship Government	Governmental organizations	Num	0=False, -1=True
	Sponsorship Foundation	A type of nonprofit organization	Num	0=False, -1=True
	Sponsorship GatesFoundation	Study funded by Bill and Melinda Gates Foundation	Num	0=False, -1=True
	Sponsorship PharmOrDevice	Industrial pharmaceutical companies, biotech companies and medical device companies.	Num	0=False, -1=True
	Sponsorship PharmOrDeviceCoName	Name of funding pharmaceutical or medical device company	Txt	
	Sponsorship HealthCare	Health care organizations	Num	0=False, -1=True
	Sponsorship ProfMembOrg	Professional membership organization, Membership organizations of professional persons formed for the advancement of the interests of their profession	Num	0=False, -1=True
	Sponsorship None	Paper explicitly states no funding	Num	0=False, -1=True
	Sponsorship NotDetermined	No information provided	Num	0=False, -1=True
	Sponsorship Other	Other than the choices above	Num	0=False, -1=True
	Sponsorship OtherText	If other, name of the sponsorship	Txt	
**Did the paper present**				
	PaperGaveInterventionID	The relevant intervention	Num	1=Yes, 0=No
	PaperGaveComparatorID	The comparator	Num	1=Yes, 0=No
	PaperGavePopulationID	The target population	Num	1=Yes, 0=No
**Time Horizon**				
	TimeHorizonStatedID	Stated time horizon clearly	Num	1=Yes, 0=No
	TimeHorizonMultipleID	Multiple time horizons	Num	1=Yes, 0=No
	TimeHorizonUnitsID	The unit of time horizon (base case)	Num	0=Lifetime, 1=Weeks, 2=Months, 3=Years
	TimeHorizonMagnitude	Number of time unit	Num	Positive value
	TimeHorizonMultiple Lifetime	Multiple horizon, lifetime	Num	0=False, -1=True
	TimeHorizonMultiple Other	Multiple horizon, other	Num	0=False, -1=True
	TimeHorizonMultiple OtherText	If other, state time horizon	Txt	
	TimeHorizonCostDalysSame	Is the time horizon of costs and DALYs consistent?	Num	1=Yes, 0=No
**Perspective**				
	PerspectiveCorrect	Was perspective accurately identified?	Num	1=Yes, 0=No
	AuthorPerspectiveID	Perspective stated by author(s) in the article	Num	1=Societal, 2=Health care payer, 3=Limited societal, 4=Health care sector, 254=Not stated/could not

				determine, 250=Other
	AuthorOtherPerspectiveText	If other, the perspective stated by author(s)	Txt	
	ReaderPerspectiveID	Perspective judged by reader	Num	1=Societal, 2=Health care payer, 3=Limited societal, 4=Health care sector, 254=Not stated/could not determine, 250=Other
	ReaderOtherPerspectiveText	If other, the perspective judged by reader	Txt	
**Costs Included**				
	Costs Healthcare	Costs associated directly with the treatment	Num	0=False, -1=True
	Costs DirectMedical	Subcategory for direct medical costs	Num	0=False, -1=True
	Costs OutofPocket	Subcategory for out of pocket costs	Num	0=False, -1=True
	Costs Unrelatedtohealth	Costs not incurred by the health care sector	Num	0=False, -1=True
	Costs PatientTime	Subcategory for costs related to patient time	Num	0=False, -1=True
	Costs CaregiverTime	Subcategory for costs related to caregiver time	Num	0=False, -1=True
	Costs Transportation	Subcategory for costs related to transportation to a healthcare facility	Num	0=False, -1=True
	Costs ProductivityGains	Subcategory for costs related to productivity gain/loss	Num	0=False, -1=True
	Costs IncomeLoss	Subcategory for costs related to amount loss in income	Num	0=False, -1=True
	Costs NonhealthcareOther	Other non-health care costs	Num	0=False, -1=True
	Costs NonhealthcareOtherText	State descriptions of non-health care costs	Txt	
	Costs OtherSectors	Costs associated with other sectors (spillover effect)	Num	0=False, -1=True
	Costs PublicHealth	Subcategory for costs related to public health resources	Num	0=False, -1=True
	Costs Legal	Subcategory for costs related to justice system	Num	0=False, -1=True
	Costs Education	Subcategory for costs related to education	Num	0=False, -1=True
	Costs Housing	Subcategory for costs related to housing	Num	0=False, -1=True
	Costs Environment	Subcategory for costs related to environmental impact	Num	0=False, -1=True
	Costs SectorsOther	Other sectors	Num	0=False, -1=True
	Costs SectorsOtherText	State descriptions of costs included for other sectors	Txt	
	Costs Implementation	Costs associated directly with the implementation of the intervention	Num	0=False, -1=True
	Costs Salary	Subcategory for costs related to salaries	Num	0=False, -1=True
	Costs Infrastructure	Subcategory for costs related to infrastructure	Num	0=False, -1=True
	Costs Administrative	Subcategory for adminstrative costs	Num	0=False, -1=True
	Costs ImplementationOther	Other implementation costs	Num	0=False, -1=True
	Costs ImplementationOtherText	State descriptions of implementation costs	Txt	
	Costs NotDetermined	Not enough information provided	Num	0=False, -1=True
**Discounting Rate**				
	CostsDiscountedID	Were costs discounted?	Num	0=No, 1=Yes, 2=Not applicable, 3=Could not determine
	CostsDiscountRate	If yes, the discounting rate of costs	Num	
	DALYsDiscountedID	Were DALYs discounted?	Num	0=No, 1=Yes, 2=Not applicable, 3=Could not determine

	DALYsDiscountRate	If yes, the discounting rate of DALYs	Num	
**Age-Weighting**				
	DALYAgeweighting	Disutility weights age-weighted for DALY computation	Num	1=Yes<br>2= No<br>3= Could not be determined
**Ethical Issue**				
	EthicalIssue	Did the authors consider ethical issues or distributional effects?		0=No, 1=Yes
	EthicalIssueText	If yes, state the reference to the issue from the article	Txt	
**Study Currency**				
	CurrencyCountry	Currency of CE ratios	Txt	
	YearOfCurrency	The year of currency	Num	
	CurrencyYearStatedID	If the currency and year are not given, the readers assume the currency year is 2 years prior to article publication	Num	0=Estimated by reader at 2 years prior to article published,<br>1=Stated by author
	CurrencyConversion	If currency was converted to US dollars, state the method used for conversion	Num	1=Foreign exchange rate,<br>2=Purchasing power parity<br>3=Not Applicable<br>4=Not Stated
**Efficacy/effectiveness of Intervention**				
	Efficacy_clinicaltrial	Data source for intervention's efficacy is from clinical trial/s	Num	0=False, -1=True
	Efficacy_literaturereview	Data source for intervention's efficacy is from literature review	Num	0=False, -1=True
	Efficacy_metaanalysis	Data source for intervention's efficacy is from meta-analysis of clinical trials	Num	0=False, -1=True
	Efficacy_casecohort	Data source for intervention's efficacy is from case-cohort studies	Num	0=False, -1=True
	Efficacy_experts	Data source for intervention's efficacy is from expert opinions	Num	0=False, -1=True
	Efficacy_notstated	Data source for intervention's efficacy is not mentioned	Num	0=False, -1=True
	Efficacy_other	Data source for intervention's efficacy is from assumptions	Num	0=False, -1=True
	Efficacy_othertext	If other, state data sources	Txt	
**Were the economic data collected alongside a trial?**				
	AlongsideTrialID	Were the economic data collected alongside a trial?	Num	0=No, 1=Yes, 3=Not Applicable
**Future Costs**				
	FutureCosts HealthCareRelated	Future costs related to the disease targeted by the intervention	Num	0=False, -1=True
	FutureCosts HealthCareUnRelated	Future costs related to other conditions or if the economic evaluation states that all medical care was considered	Num	0=False, -1=True
	FutureCosts NonHealthCare	Future costs outside of health care, other costs for added life years	Num	0=False, -1=True
	FutureCosts Productivity	Future costs associated with increased productivity and resource consumption	Num	0=False, -1=True
	FutureCosts None	No estimation of future costs	Num	0=False, -1=True
	FutureCosts NotApplicable	Time horizon is only 1, 2 years or less.	Num	0=False, -1=True
**Incremental Analysis were**				
	IncrementalAnalysis NotReported	Incremental analyses were not reported.	Num	0=False, -1=True
	IncrementalAnalysis Correct	The incremental analysis was conducted and calculated correctly	Num	0=False, -1=True
	IncrementalAnalysis Incorrect	The incremental analysis was conducted but calculated incorrectly	Num	0=False, -1=True

	IncrementalAnalysis Recalculated	If incremental analysis was calculated incorrectly and there were sufficient data of cost and effectiveness; readers recalculated the analysis	Num	0=False, -1=True
	IncrementalAnalysis Incalculable	Sufficient data were not provided in the study to repeat the ICER calculation	Num	0=False, -1=True
**Cost-effectiveness threshold**				
	Cutoff 50K	US$50,000	Num	0=False, -1=True
	Cutoff 100K	US$100,000	Num	0=False, -1=True
	Cutoff 3XGDP	WHO guideline of 3 times GDP of country per capita per DALY averted	Num	0=False, -1=True
	Cuttoff GDP	GDP of country per capita per DALY averted	Num	0=False, -1=True
	Cutoff Range	A range in US$ or other currency as reported	Num	0=False, -1=True
	Cutoff RangeLower	Lower value of the range	Num	Any numeric value
	Cutoff RangeUpper	Upper value of the range	Num	Any numeric value
	Cutoff Other	Other threshold values	Num	0=False, -1=True
	Cutoff OtherText	Enter text for other value of threshold	Txt	
	Cutoff NoneNS	Not stated the threshold	Num	0=False, -1=True
**Sensitivity Analysis**				
	SensitivityAnalysis Bounded	Best and worst case scenario; C/E ratios are specified for a lower bound and/or an upper bound in a simulation	Num	0=False, -1=True
	SensitivityAnalysis Probabilistic	Parameters are varied over a distribution	Num	0=False, -1=True
	SensitivityAnalysis Univariate	Also termed a one-way sensitivity analysis; either term means that one parameter of the model is changed at a time.	Num	0=False, -1=True
	SensitivityAnalysis Multivariate	A general term that encompasses any type of sensitivity analysis where multiple parameters are changed simultaneously	Num	0=False, -1=True
	SensitivityAnalysis NoneNS	Authors did not mention sensitivity analysis or did not conduct a sensitivity analysis	Num	0=False, -1=True
	SensitivityAnalysis PerformedUnspecified	Authors mentioned the sensitivity analysis was conducted but did not state the type of analysis	Num	0=False, -1=True
**Acceptability curves**				
	AcceptabilityCurvesID	Did the analyses include acceptability curves?	Num	0=No, 1=Yes
**Overall quality of analysis**				
	Rating	Overall quality of analysis (judged by reader)	Num	1, 1.5, 2, 2.5, 3, 3.5, 4, 4.5, 5, 5.5, 6, 6.5, 7
	Rating HealthEco	Health economic methodology	Num	0=No problem, 1=Major problem, 2=Can't evaluate
	Rating Uncertainty	Uncertainty consideration	Num	0=No problem, 1=Major problem, 2=Can't evaluate
	Rating Transparency	Transparency of the model	Num	0=No problem, 1=Major problem, 2=Can't evaluate
**Comments**				
	GeneralComments	General comments	Txt	

**Ratio Information**				
**Target Population**				
	TargetPopulation HealthID	Health status of target population	Num	1=General, 2=Specific disease
	TargetPopulation HealthText	Specific disease statement	Txt	
	TargetPopulation Gender	Gender	Num	1=Female, 2=Male, 3=Both
	TargetPopulation Age_05	Children, age 0-5	Num	0=False, -1=True
	TargetPopulation Age_611	Children, age 6-11	Num	0=False, -1=True
	TargetPopulation Age_1218	Adolescents, age 12-18	Num	0=False, -1=True
	TargetPopulation Age_1940	Age 19-50	Num	0=False, -1=True
	TargetPopulation Age_4164	Age 41-64	Num	0=False, -1=True
	TargetPopulation Age_65	Age over 65	Num	0=False, -1=True
	TargetPopulation Age_adult	Adult, not specified age	Num	0=False, -1=True
	TargetPopulation Age_unknown	Not specified	Num	0=False, -1=True
	TargetPopulation Age_specifyage	Specify age/age range	Txt	
	TargetPopulation Country	Country/Region	Txt	
	TargetPopulation Other	Other characteristics of the population (e.g. co-morbidities)	Txt	
**Intervention**				
	Intervention Paragraph	The specific drug, device or other health care intervention	Txt	
**Comparator**				
	ComparatorID	Type of comparator	Num	1=Placebo, 2=Standard care, 3=None, 4=Other
	Comparator StandardCareText	Description of standard/usual care, if comparator is a standard care	Txt	
	Comparator OtherText	Other description of comparator	Txt	
**Cost/DALY Averted from article**				
	$/DALYAverted from Article	Incremental cost-effectiveness ratio from article (ΔCosts/ΔDALYs)	Num	Positive value
**Ratio Perspective**				
	Ratio PerspectiveID	Perspective of this specific ratio	Num	1=Societal, 2=Health care payer, 3=Limited societal, 4=Health care sector, 254=Not stated/could not determine, 250=Other
	Ratio PerspectiveOther	If other, state the perspective	Txt	
**Sequential analysis**				
	Sequential Analysis	Is this ratio reported as part of a sequential analysis?	Num	-1=Yes, 0=No
**Ratio calculation**				
**Population size**				
	PopulationID	Was real population size reported?	Num	0=Not applicable, 1= Stated
	PopulationSize	The number of people in a given population that have the same health condition as the study population	Num	
	Population sizevalue	The population size as reported in the study or estimated from other sources	Num	1=From study, 2=Estimated, 3=Could be determined
	PopulationSizeEstimationText	If population size is estimated from a different source; enter relevant information here	Txt	
**Per Person**				
	ComparatorDirectCostsPerPerson	Direct medical costs of comparator	Num	

	InterventionDirectCostsPerPerson	Direct medical costs of intervention	Num	
	DirectCostsPerPerson	(Direct medical costs of intervention)-(Direct medical costs of comparator)	Num	
	ComparatorNonHealthCostsPerPerson	Non-health care costs of comparator	Num	
	InterventionNonHealthCostsPerPerson	Non-health care costs of intervention	Num	
	NonHealthCostsPerPerson	(Non-health care costs of intervention)-(Non-health care costs of comparator)	Num	
	ComparatorTotalCostsPerPerson	Total costs of comparator	Num	
	InterventionTotalCostsPerPerson	Total costs of intervention	Num	
	TotalCostsPerPerson	(Total costs of intervention)-(Total costs of comparator)	Num	
	ComparatorDALYsPerPerson	DALYs averted for comparator	Num	
	InterventionDALYsPerPerson	DALYs averted for intervention	Num	
	DALYsAverted PerPerson	(DALYs of intervention)-(DALYs of comparator)	Num	
**Entire population**				
	ComparatorDirectCosts	Direct medical costs of comparator	Num	
	InterventionDirectCosts	Direct medical costs of intervention	Num	
	DirectCosts	(Direct medical costs of intervention)-(Direct medical costs of comparator)	Num	
	ComparatorNonHealthCosts	Non-health care costs of comparator	Num	
	InterventionNonHealthCosts	Non-health care costs of intervention	Num	
	NonHealthCosts	(Non-health care costs of intervention)-(Non-health care costs of comparator)	Num	
	ComparatorTotalCosts	Total costs of comparator	Num	
	InterventionTotalCosts	Total costs of intervention	Num	
	TotalCosts	(Total costs of intervention)-(Total costs of comparator)	Num	
	ComparatorDALYs	DALYs averted for comparator	Num	
	InterventionDALYs	DALYs averted for intervention	Num	
	DALYsAverted	(DALYs averted for intervention)-(DALYs averted for comparator)	Num	
**Cost/DALY Averted from article quadrant**				
	$/DALYAverted Quadrant	This refers to the net impact in costs and DALYs averted for the intervention, as represented in the cost-effectiveness ratio.	Num	1=Quadrant I, 2=Quadrant II, 3=Quadrant III, 4=Quadrant IV, 5=Could not determine
**Other CE Ratios reported**				
	$/LY from Article	Incremental cost-effectiveness ratio from article (ΔCosts/ΔLYs)	Num	Positive value
	$/LY Comments	Comments for the ratio $/LY	Txt	
	Cost/QALY	If cost/QALY ratio is reported	Num	1=Yes, 0=No
	Cost/QALYText	Enter cost/QALY ratio	Txt	
	Other$/CE	Other incremental cost-effectiveness ratio reported	Num	0=False, -1=True
	OtherCEText	If yes, text of other CE ratios	Txt	
	OtherCE RatioComment	Comments for other CE ratios	Txt	
	NoOtherCEs	No any other incremental cost-effectiveness ratio measured in other units of effectiveness	Num	0=False, -1=True
**Budget Impact**				
	ReportBudgetImpactID	Did the study report a budget impact on a particular payer?	Num	1=Yes, 0=No

	ReportBudgetImpactText	If yes, the amount of budget impact	Txt	
	BudgetImpactTimeline	State the time line provided for budget impact	Txt	
**Uncertainty Analyses**				
**Graphic**				
	Uncertainty Graphic_ACU	Cost-effectiveness acceptability curve presented	Num	0=False, -1=True
	Uncertainty Graphic_Scatter	Cost-effectiveness scatter plot presented	Num	0=False, -1=True
	Uncertainty Graphic_Tornado	Tornado diagram presented	Num	0=False, -1=True
	Uncertainty Graphic_None	None graph presented	Num	0=False, -1=True
**Probabilistic**				
	Probabilistic SA_ACU	Probabilistic sensitivity analysis was presented in cost-effectiveness acceptability curve	Num	0=False, -1=True
	Probabilistic SA_CI	Probabilistic sensitivity analysis was presented as confidence interval(s)	Num	0=False, -1=True
	Probabilistic SA_None	No probabilistic sensitivity analysis was presented	Num	0=False, -1=True
**Acceptability curve**				
	ACU I_%	Percent of simulations/patients meeting a threshold identified by ACU I_ICER	Txt	
	ACU I_ICER	Threshold value corresponding to ACU I_%.	Txt	
	ACU II_%	Percent of simulations/patients meeting a threshold identified by ACU II_ICER	Txt	
	ACU II_ICER	Threshold value corresponding to ACU II_%.	Txt	
	ACU III_%	Percent of simulations/patients meeting a threshold identified by ACU III_ICER	Txt	
	ACU III_ICER	Threshold value corresponding to ACU III_%.	Txt	
**Confidence interval**				
	Confidence Interval	Confidence interval reported	Num	1=Yes, 0=No
	CI_ICER	If reported confidence interval of the ratio	Num	0=False, -1=True
	CI_ICERText	State the confidence interval of the ratio	Txt	
	CI_Costs/DALYs	If reported costs and DALYs averted confidence interval separately	Num	0=False, -1=True
	CI_Costs/ DALYs CostsText	State the confidence interval of the costs	Txt	
	CI_Costs/ DALYs DALYs Text	State the confidence interval of DALYs averted	Txt	
**Others**				
	SA Input I	Input variables causing most uncertainty	Txt	
	SA Input II	Input variables causing most uncertainty	Txt	
	SA Input III	Input variables causing most uncertainty	Txt	
	SA Input IV	Input variables causing most uncertainty	Txt	
	SA Input V	Input variables causing most uncertainty	Txt	

	SA ModificationType I	Indicating change in input variable	Txt	
	SA ModificationType II	Indicating change in input variable	Txt	
	SA ModificationType III	Indicating change in input variable	Txt	
	SA ModificationType IV	Indicating change in input variable	Txt	
	SA ModificationType V	Indicating change in input variable	Txt	
	SA Output I	ICER point estimate/range	Txt	
	SA Output II	ICER point estimate/range	Txt	
	SA Output III	ICER point estimate/range	Txt	
	SA Output IV	ICER point estimate/range	Txt	
	SA Output V	ICER point estimate/range	Txt	
	RatioUncertainty	The result from sensitivity analyses to evaluate the impact of uncertainties on the ratio results.	Txt	
**Comments**				
	RatioComments	General comments on ratios	Txt	
**Cost/DALY Averted from reader**				
	$/DALYAverted from Reader	Re-calculated by reader, based on the costs and DALYs value reported in the article	Num	

Disutility/Disability Weights form				
**Not reported weight**				
	NoWeight	Paper does not report any weight values	Num	0=False, -1=True
**Health State**				
	HealthState	The utility weight for the specific health state/condition	Txt	
**Base Case Weight**				
	Weight	The disutility weight value for the base case analysis	Num	
**Weight range for sensitivity analysis**				
	WeightRangeLower	Lower value of weight range	Num	
	WeightRangeUpper	Upper value of weight range	Num	
	WeightRangeOther	If not reported in numbers, state the range here	Txt	
**Characteristics of Population**				
	Characteristics Gender	Gender	Num	1=Female, 2=Male, 3=Both
	Characteristics Age_05	Children, age 0-5	Num	0=False, -1=True
	Characteristics Age_611	Children, age 6-11	Num	0=False, -1=True
	Characteristics Age_1218	Adolescents, age 12-18	Num	0=False, -1=True
	Characteristics Age_1940	Age 19-50	Num	0=False, -1=True
	Characteristics Age_4164	Age 41-64	Num	0=False, -1=True
	Characteristics Age_65	Age over 65	Num	0=False, -1=True
	Characteristics Age_adult	Adult, not specified age	Num	0=False, -1=True
	Characteristics Age_unknown	Not specified	Num	0=False, -1=True
	Characteristics Age_specifyage	Specify age/age range	Txt	
**Data source of the weight**				
	WeightDataFromPrimarySource	Data is derived from author's collection of disutility weights generated specifically for this study	Num	0=False, -1=True
	WeightDataFromSecondarySource	Data is derived from another source other than this study	Num	0=False, -1=True
	WeightDataFromNoneNS	Not stated or could not determine	Num	0=False, -1=True
**Reference (if datasource = "Secondary")**				
	Disability GBD	If the study used disability weights from WHO's Global Burden of Disease study	Num	0=False, -1=True
	Disability GBD1990	WHO's Global Burden of Disease 1990 study	Num	0=False, -1=True
	Disability GBD1990Ref	If yes, cite the reference number of the article	Txt	
	Disability GBD2001	WHO's Global Burden of Disease 2001-2002 study	Num	0=False, -1=True
	Disability GBD2001Ref	If yes, cite the reference number of the article		
	Disability GBD2004	WHO's Global Burden of Disease 2004 study	Num	0=False, -1=True
	Disability GBD2004Ref	If yes, cite the reference number of the article	Txt	
	Disability GBD2010	WHO's Global Burden of Disease 2010 study	Num	0=False, -1=True
	Disability GBD2010Ref	If yes, cite the reference number of the article	Txt	
	Disability GBDOther	Other WHO's Global Burden of Disease study	Num	0=False, -1=True
	Disability GBDOtherRef	State the name of WHO's Global Burden of Disease study	Txt	
	Disability Other	Other study used to report disability	Num	0=False, -1=True

		weights		
	Disability OtherRef	If yes, cite the reference number of the article	Txt	
	Disability NotStated	Not enough information provided	Num	0=False, -1=True
	PreviousStudy Lifeexpectancy	Study used to report mortality or life expectancy	Txt	
	PreviousStudyCite	Record the reference number from article	Txt	
**Sample Population type**				
	PrefScoreSource Community	Community: community means a representative sample of non- patients	Num	0=False, -1=True
	PrefScoreSource Clinician	Clinician: clinician judgment was the basis for the utility weights, or if clinicians were surveyed	Num	0=False, -1=True
	PrefScoreSource Patient	Patient: patients in the health state in question were queried	Num	0=False, -1=True
	PrefScoreSource Author	Author/Experts: when the authors/ experts made an assumption about the disutility weight for the disease state.	Num	0=False, -1=True
	PrefScoreSource RelationsProxies	Relatives/Proxies: If the patient's parent, significant other or child was queried	Num	0=False, -1=True
	PrefScoreSource NotStated	Unknown/Not stated: no information provided	Num	0=False, -1=True
	PrefScoreSource Other	Other: other than choices above	Num	0=False, -1=True
	PrefScoreSource OtherText	If other, the type of sample population	Txt	
**Sample Size**				
	WeightSampleType SampleSize	This refers to the weight study sample size, not the population in the Ratio table.	Num	
**Comments**				
	WeightComments	General comments on weights	Txt	

III. GLOSSARY

METHOD INFORMATION

1. Intervention types

This variable describes the primary intervention that was evaluated. Generally, one intervention is compared against other alternatives; i.e.: the intervention is the thing that is being changed with an aim to improve health outcomes

*<u>**Note: A single study can analyze an intervention or interventions that pertain to multiple intervention categories (categories are not mutually exclusive).</u>*

Care Delivery: Provision of clinical care or health care services; development of health care facilities or distribution of personnel (e.g. a policy that changes the nurse-to-patient ratio, patient self-management program)

Health Education or Behavior: Interventions designed to educate or inform individuals about behaviors that promote, maintain or restore health (e.g. smoking cessation and prevention program)

Pharmaceutical: Any drug, biotech product or agent that produces an effect through chemical, metabolic, or immunological action within or on the body (e.g. Lovastatin, Herceptin)

Surgical: As defined by the Oxford dictionary, surgery is “the treatment of injuries or disorders of the body by incision or manipulation, especially with instruments”. Generally, surgical interventions are characterized by incision or cutting intended to investigate and/or treat a pathological condition, disease, or injury, or to help improve bodily function or appearance (e.g. transplantation, appendectomy)

Immunization: Vaccination intended to prevent infectious disease (e.g. flu vaccine, HPV vaccine)

Diagnostic: An intervention or technology used to determine if and what type of disease is present (e.g. imaging, biopsy, PET scan, x-rays, in-vitro testing)

Medical Procedure: An activity directed at or performed on an individual with the object of improving health, or treating disease or injury. Medical procedures are generally non-surgical, non-diagnostic in nature (e.g. casting or splinting of broken bones)

Medical Device: A medical device is an instrument, apparatus, implant, in vitro reagent, or similar or related article that is used to diagnose, prevent, or treat disease or other conditions. Medical devices can range from simple tongue depressors and bedpans to complex programmable pacemakers and laser surgical devices. Please note that medical devices may or may not require a surgical or implantation procedure (e.g. pacemaker, insulin pump, leg brace and crutches)

Screening: Refers to measures that detect disease (or test for risk factors) before the onset of symptoms (e.g. breast cancer screening – mammogram)

Nutrition: Interventions to address malnutrition or poor diet by promoting dietary changes (using education or other policy changes). Examples include programs to increase consumption of fruits and vegetables, nutrition education programs, and iron fortification/supplementation programs.

Maternal/Neonatal Care: Interventions to address maternal care received at antenatal visits, at delivery, and for neonates. Examples include family planning, skilled attendance at birth, screening for pre-eclampsia, care during child birth, and screening for congenital diseases.

Environmental remediation: Interventions aimed at *removal of contamination* from water bodies, soil or other areas to improve human health. Examples include measures to address pollution or to protect water supplies.

Environmental augmentation: Interventions to inhibit or limit environmental threats to health. An example is provision of bed nets to reduce the risk of arthropod-vector infections.

Legislation/Regulation: Taxes and regulatory measures that influence access to certain foods and other consumed substances (e.g., cigarettes). Examples include alcohol taxation, taxation of soft drinks, and controls on the use of tobacco.

Other: Any intervention not described above (e.g. injury prevention, food safety, or environmental health)

2. **Prevention Stage:** Prevention stage definitions (*Ref: Concise Medical Dictionary. Oxford University Press, 2007. Oxford Reference Online. Oxford University Pres*).

   Primary: Interventions involved with the avoidance of the onset of disease by behavior modification or treatment, such as immunization, promotion of safety equipment use (e.g. seat belts), health education (e.g. anti-smoking campaigns), promotion of improved nutrition and prenatal care.

   Secondary: Interventions involved with the avoidance or alleviation of disease by early detection and appropriate management. Secondary prevention includes population screening to identify disease in asymptomatic people to enable timely treatment.

   Tertiary: Interventions involved with treatment to reduce complications and progression of established disease, e.g. remedial exercises for contractures, care of pressure points and bladder function in paraplegia, cardiac rehabilitation following myocardial infarction, stroke rehabilitation, and screening of people with diabetes for diabetic retinopathy.

3. **Country of Study:** The geographical perspective of the economic evaluation; i.e. the country to which the study's results are applied. For example, if country is Ghana, then the CE ratio represents the expected costs and benefits, if intervention were adopted in Ghana, irrespective of what country individual data were derived from. If the intervention is being applied across more than four countries, then the *Country of Study* should be flagged as 'international'

4. **Intervention Scope:** Intervention scope is defined as the population aggregation level at which the intervention is targeted. The intervention can target the local, regional/provincial, or national level.

This variable is essential for understanding the resources used at a given implementation scale and can help to inform estimation of costs if the intervention is scaled up.

Local: Interventions that target a relatively small or limited population; e.g., the population of a school, village, town, or city. Unless explicitly described at a larger scale, then the default intervention scope is 'Local'.

Provincial: Interventions addressing the population of a province or sub-national region. An example is hepatitis B immunization in the Amhara region of Ethiopia.

National/Regional: Interventions that target a population within a particular country or multiple countries. An example is a neonatal hearing screening program in China. Please note that 'Regional' denotes a multinational/international scope.

Could not be determined: indicates that the study does not provide sufficient information to determine the scope of the intervention.

5. **Primary Affiliation of Author(s):** Authors' primary association or membership during time of publication

   University/Academic Organization: Universities, institutes/centers or any other academic organizations; teaching hospitals and medical schools are included

   Government Organization: Institutes, centers or organizations that are part of a governmental or international body. Please note that this category encompasses all groups, consortia, and specialized agencies of the United Nations, including the World Health Organization (WHO), United Nations Children's Fund (UNICEF), and the United Nations Development Group (UNDG).

   Contract Res./Consultant: Contract researchers or consulting firms (e.g. IMS Health, UBC, i3 Innovus)

   Health Care Organization: Health care providers (e.g. insurance companies, hospital, HMOs)

   Pharm./Med Device Industry: Industrial pharmaceutical companies, biotech companies, or medical device companies (e.g. Johnson and Johnson, Pfizer, Medtronic)

   None: No author affiliation information provided in the article

   Other: Other than the choices above

6. **Study Sponsorship/Funding**

   Describe all funding sources that supported the study. Please be sure to scan for non-specific indications and acknowledgement of 'support'.

   Government: See Q#5. Government Org.

Health Care Org: See Q#5. Health Care Org.

Foundation: A nonprofit, non-governmental organization (e.g. Robert Wood Johnson Foundation, PhRMA Foundation)

Gates Foundation: The study was sponsored/funded by the Bill & Melinda Gates Foundation

Professional Membership Org.: Professional membership organization, membership organizations of professional persons formed for the advancement of the interests of their profession (e.g. Academy Health, ISPOR)

Pharm./Med Device Co.: Industrial pharmaceutical companies, biotech companies, or medical device companies (e.g. Johnson and Johnson, Pfizer, Medtronic)

Others: Other than the choices mentioned above

Not stated/Could be not determined: No information provided. Please note that if the source of study sponsorship or funding is not described, then the reviewer should default to the authors' primary affiliation, while also indicating that the funding source is not explicitly stated.

None: Paper explicitly states "no funding"

REPORTING OF METHOD

7. Did the paper clearly present…

A. The relevant intervention, B. The comparator, C. Target population

These are binary variables intended to describe whether or not each item was clearly described in the paper. The item is clearly presented if two independent readers interpret the information the same way.

8. Time horizon/s analyzed

The time horizon of an analysis reflects the length of time over which health and outcomes associated with the intervention are tracked, not the duration of data collection (see *Gold MR. et al. Cost-Effectiveness in Health and Medicine*).

Please note that given the nature of how DALYs are calculated, the default should be a 'lifetime' horizon, unless otherwise stated or indicated in the paper. If the authors indicate that there are different time horizons for costs and outcomes, please describe the longest horizon and make a note of the discrepancy in the 'Other time horizons' field described below.

A. Stated: Whether or not the paper clearly reported a time-horizon

B. What was the base case time horizon analyzed?

Lifetime: Whether or not study used a lifetime horizon in the base case analysis

Other: If a non-lifetime horizon was reported in the base case, please describe its duration

Units: Days/Weeks/Years. This is the units that were used in the main/base-case analysis. The exact determination of the main/base case is the responsibility of the study author to disclose. If no explicit disclosure is made, the main/base case horizon is the same horizon used for the main/base case results.

C. Multiple: If the paper conducted cost-effectiveness analyses from multiple time horizons (e.g. both 5 years analysis and lifetime analysis).

D. Lifetime: If the alternate case is a lifetime analysis.

Other time horizons/text: True if the alternate case is NOT lifetime. If this box is checked, please be sure to report the duration (numerical value).

9. **Perspective**

Perspective is the viewpoint from which costs and benefits are calculated. The point of view of the analysis determines which costs and health effects are considered. For example, patients, providers, health payers, and society may view costs differently: patients may consider only out of pocket or co-payment costs; providers may consider the health benefit for an individual patient but weigh it against the overall public health implications; health payers may consider how the cost of an intervention compares to alternatives; and society may also consider the broader costs and trade-offs associated with the intervention.

The perspective should be reported as determined by both author and reviewer. If multiple perspectives are presented, please default to the base case or reference case. Also, please be careful to note if the authors explicitly state the analysis perspective.

As Judged by Author: The perspective used in the study as stated by the author.

As Judged by Reviewer: The perspective used in the study as judged by the reviewer. For example, a study might state ‘societal perspective’ but fail to include non-health care costs. In this case the perspective as judged by the reviewer would be ‘health care sector’.

<ins>Note</ins>- If the author states ‘societal’ and only included costs pertaining to ‘limited societal’, in that case you will still consider that the perspective was identified correctly.

Health Care Payer: Includes only monetary costs incurred by a (typically 3rd party) health care payer (e.g. Medicare/Medicaid, British National Health Service, an HMO or private health care plan).

Health Care Sector: *Health Care Sector* perspective is similar to *Health Care Payer*. However, the *Health Care Sector* perspective accounts for all monetary costs of health care, regardless who bears the cost (includes out-of-pocket costs).

> Please note that when considering DALY studies conducted in low and middle income countries, a key distinction between the *Health Care Sector* and *Health Care Payer* perspectives is that the *Health Care Sector* perspective includes out-of-pocket costs and *Health Care Payer* does not.

Limited Societal: *Limited Societal* perspective analysis includes non-healthcare related costs like patient time and productivity that are a direct result of the intervention. The perspective is designated "limited societal" if at least one such cost is included (e.g. unpaid caregiver time, productivity impacts, patient time, patient out of pocket costs).

Societal: The *Societal* perspective is broader than limited societal perspective. It represents the overall public interest by including social opportunity costs where the use of limited resources (such as personnel, hospital beds, donor organs, or budgets) results in the loss of opportunity to use those funds (or resources) for other purposes. This goes beyond the *Limited Societal* perspective in that the analysis accounts for impacts on other sectors, such as environmental, educational, and judicial.

Not Stated/Could Not Be Determined: Authors did not provide sufficient information to determine type of costs or benefits evaluated

Other: Authors report a perspective other than societal or health care payer

10. Costs Included

Health care costs- This category includes costs associated directly with the treatment. This includes direct medical costs and any other long-term medical costs incurred by the patient (out of-pocket medical costs). E.g., vaccine costs, drug cost, physician visit cost, follow-up visit cost, hospitalization cost, outpatient cost.

Costs unrelated to treatment-- This category includes costs not incurred by or specific to the health care sector (e.g., unpaid caregiver time, productivity impacts, patient time, income loss).

Patient time- patient time lost due to illness or travel

Caregiver time- costs associated with time and care provided by caregiver

Transportation- costs associated with travel to health care facility

Productivity gains- costs associated with on-job productivity losses or presenteeism. Presenteeism is illnesses and medical problems that undermine job performance in the workforce.

Income loss- Income lost due to lost days of work

Other- Other costs unrelated to treatment

Other Costs/Sectors- Other costs include, but are not limited to:-

Public health- Costs related to public health resources, e.g., homeless shelters, public services, wellness programs, and food provisions.

Legal/criminal justice- Costs related to arrests, convictions, time spent in jail/prison.

Education- Costs related to paying for school and college.

Housing- Costs related to public and private housing, property value.

Environment- Costs related to environmental impact of the intervention.

Implementation costs- Implementation costs include costs associated with setting up of the intervention. For e.g., capital costs for offices, personnel salaries, materials and supplies, utilities, training and transport.

Personnel salaries- includes salaries associated with staff involved in the intervention

Infrastructure- costs associated with setup of the intervention

Administrative costs- Expenses incurred in controlling, directing and managing an organization/intervention

Other- Other costs related to the implementation of the intervention

Could not be determined- If the study does not provide sufficient information to determine the type of costs included.

DISCOUNTING

11. Discounting

The aim of these two binary variables is to determine whether or not appropriate discount rates were applied for both costs and DALYs.

Money available or spent now is more valuable than money available or spent in the future because of opportunity costs. Discounting quantifies this time preference and places all economic costs in terms of the present value of money. When health effects can be valued in monetary terms over a period of time, then both health effects and associated costs can be discounted.

If the time horizon is less than one year, then no discount rate should be applied.

Please note: The Global Burden of Disease (GBD) 1990, 2001-2, and 2004 all discount costs and benefits at 3 percent annually, while GBD 2010 does not recommend discounting.

a. Were costs discounted?

State the discount rate reported in the study for costs.

b. Were DALYs discounted?

State the discount rate reported in the study for costsDALYs.

12. Age weighting

Age-weighting increases the value of life years from ages 9 to 24 relative to the value of years of life at other ages.

Yes- Non-uniform age-weighting is typically used in cost per DALY literature. In non-uniform age-weighting the relative value of a year of life rises rapidly from zero at birth to a peak in the early twenties, after which it steadily declines. Years during young adulthood are valued more than the years lived at the beginning and end of the normal life span.

Please note that GBD 1990 and 2004 recommended non-uniform age-weighting. GBD 2001-2 recommended uniform age-weighting.

No- No age-weighting used for DALY computations. Please note that GBD 2010 does not recommend age- weighting.

Could not be determined- Study does not provide sufficient information to determine if it used age-weighting.

13. Ethical issues or distributional effects

Cost effectiveness analysis can provide important information about the economic efficiency of interventions and can help health care decision makers choose between competing alternatives. Ethical issues such as equity, fairness, and distributional justice can be important to discussion of health care resource allocation. Please indicate if the authors discussed, described, or mentioned ethical issues or distributional effects related to the implementation of the intervention being studied.

****If yes, please copy-paste the relevant text in the provided field.**

COST MEASUREMENT

14. Currency of the CE ratio numbers

A. Currency- The currency used to quantify or describe monetary costs.

Please note that it is not uncommon for costs to be reported in the 'international dollar', a hypothetical unit of currency that has the same purchasing power parity that the U.S. dollar had in the United States at a given point in time. Figures expressed in international dollars cannot be

converted to another country's currency using current market exchange rates; instead they must be converted using the Purchasing Power Parity exchange rate used in the study.

B. Year

If the currency and year are not given, assume the currency used in the analysis is denominated in currency of the country in which the analysis is presented and valued at 2 years prior to article publication.

C. Currency Conversion- If the authors have converted the currency to US dollars, state the conversion method used: Foreign exchange rate or Purchasing power parity (PPP).

15. Data source for intervention's efficacy/effectiveness

State the data source(s) used for determining the efficacy/effectiveness of the intervention.

Trial/s
Literature review
Meta-analysis of clinical trials
Case-cohort studies
Expert opinion
Not Stated
Other

16. Data collected alongside a trial?

There are two primary methods for economic data to be collected alongside a trial:

- Implicit/Imputed Measurement: Resource utilization (e.g. number of hospital visits, physician encounters, number of prescriptions, etc.) is recorded in the trial, and unit costs (which may come from a national health plan or government sponsored insurance, etc.) assigned to each type of resource utilization.
- Direct Measurement: Overall (medical) costs are collected from each arm of the trial.

17. Future costs and savings associated with changed lifespan

Good health (averted DALYs) is one of the most important things to individuals and also brings many other benefits, including enhanced access to education and the job market, an increase in productivity and wealth, reduced health care costs, good social relations, and of course, a longer life. Please indicate if the authors have described any future costs or savings related to added life years resulting from the intervention.

Health Care Related to Indicated Condition: Future costs related to the disease targeted by the intervention (e.g. continued cancer screening costs due to prevention of cancer, annual costs of medication or monitoring after intervention periods, follow-up visits)

Health Care Unrelated to Indicated Condition: Future health care costs related to other diseases or conditions (e.g. treatment costs for Alzheimer's disease incurred because person did not die from cancer), or if the economic evaluation states that all medical care was considered.

Non-Healthcare Cost: Future costs outside of health care, other than costs for added life years. E.g., caregiver costs, medical transportation costs.

Productivity and Consumption: Discrete costs and gains associated with an individual's role as a producer and consumer of goods and services

Not Applicable: Time horizon does not allow for estimation of future costs/gains. Please note that 'Not Applicable' should only be indicated if the intervention was not expected to cause a change in life expectancy.

None: The authors made no estimation of change in life expectancy, or no estimation of future costs/savings.

REPORTING OF RESULTS

18. Incremental analyses

The incremental cost-effectiveness ratio (ICER) is a commonly used equation in cost-effectiveness analysis. An ICER is the ratio of the change in costs to incremental benefits of a therapeutic intervention or treatment:

$$\text{ICER} = \frac{Costs_{Intervention} - Costs_{Comparator}}{DALY_{Intervention} - DALY_{Comparator}}$$

Not Reported: Incremental analyses were not reported
Correct: The incremental analysis was conducted and calculated correctly
Incorrect: The incremental analysis was conducted but calculated incorrectly
Recalculated: If incremental analysis was calculated incorrectly and there were sufficient data of cost and effectiveness; readers recalculated the analysis
Not enough data to recalculate: Sufficient data were not provided in the study to repeat the ICER calculation

19. What explicit values did the author give for the cost-effectiveness threshold?

Please report the incremental cost-effectiveness ratio (ICER) cut-off that the study authors used to assess whether the intervention was cost-effective.

Please note that the World Health Organization (WHO) recommends the use of the per capita gross domestic product (GDP) as a benchmark for assessing an intervention's cost-effectiveness. In particular, the WHO identifies both the per capita GDP, and three times the per capita GDP per DALY averted as useful benchmarks.

US$ 50K: Represents $50,000 in US dollars.

US$ 100K: Represents $100,000 in US dollars.

WHO Guideline- 3 times the per capita GDP per DALY averted

WHO Guideline- Per capita GDP per DALY averted

Range: Cost-effectiveness threshold range, reported with **RangeLower** and **RangeUpper**

Other: Other cost-effectiveness thresholds**;** Reported as **OtherText**

None: No cost-effectiveness threshold specified.

<u>SENSITIVITY ANALYSIS</u>

20. Sensitivity analyses: Sensitivity analyses evaluate the impact of uncertainties on the cost-effectiveness ratio

There can be considerable uncertainty regarding the parameters used to measure costs and health effects. To help identify the most influential or important parameters and to assess the degree to which uncertainty in these parameters could affect the overall results, cost-effectiveness analyses usually perform multiple sensitivity analyses in which one or more parameters are varied across reasonable ranges.

Sensitivity analyses typically assess the effect of varying individual parameters related to cost-effectiveness one at a time. A more contemporary approach called Monte Carlo simulation permits all parameters to be varied simultaneously. These sophisticated analyses yield a cost-effectiveness acceptability curve, which accounts for uncertainty in all model estimates.

Univariate: Also referred to as a “one-way” sensitivity analysis, univariate analyses change one parameter of the model is at a time in order to examine the impact that the change has on the model’s results. For example, it might be shown that by changing the clinical effectiveness of an intervention by 10%, the cost-effectiveness ratio falls by 20%.

A tornado diagram is a graphical display of several one-way sensitivity analyses at a time

Multivariate: A general term that encompasses any type of sensitivity analysis that evaluates how the cost-effectiveness ratio is affected when more than one parameter is changed simultaneously

Bounded: Refers to analyses that present a range of cost-effectiveness estimates (e.g., cost-effectiveness estimates corresponding to optimistic and pessimistic assumptions for a parameter or set of parameters) or specify any probability or relative likelihood information.

Probabilistic: Refers to analyses that provide <u>probability information</u> for a range of plausible cost-effectiveness values. For example, a probabilistic analysis may describe this range as a probability distribution. Alternatively it might quantify how much more likely one set of cost-effectiveness estimates are compared to another. This latter approach may be taken within Bayesian methods. The methods sections for papers that conduct probabilistic sensitivity analyses may refer to “Monte Carlo simulation”, “Bayesian Probabilistic Analyses”, or “bootstrapping”.

None/Not stated: No information provided; cannot determine if a sensitivity analysis was conducted

Performed but type not specified: Authors mentioned that sensitivity analysis was conducted but did not state the type of analysis

21. Did the analyses include acceptability curves?

The cost-effectiveness acceptability curve (CEAC) is one method of presenting probabilistic sensitivity analysis results. The CEAC plots the probability that an intervention is favorably cost-effective (i.e. has a cost-effectiveness ratio below the societal willingness to pay threshold) on the vertical axis, and societal willingness to pay for one additional DALY averted on the horizontal axis. For a particular willingness to pay threshold, the probability that an intervention is favorably cost-effective can be between zero and one because uncertainty analysis provides a distribution of cost-effectiveness ratios for the intervention. Part of that distribution may be below the indicated threshold, while the rest is above the threshold.

DISCUSSION SECTION

22. Overall quality of the analysis

Cost-effectiveness analysis is complex, and studies making claims regarding cost-effectiveness should be scrutinized in detail. Many features and aspects of a study can impact its quality, but some of these factors are unique or should only be considered in specific settings.

Quality assessment is included here as a subjective rating score from 1 (lowest quality) to 7 (highest quality). Scores should reflect the following considerations (in rough order of importance):
1) Whether cost/DALY ratios are correctly computed
2) Comprehensive characterization of uncertainty (i.e., either probabilistic or non-probabilistic evaluation of how changes in plausibly important assumptions influence the results)
3) Correct treatment and explicit specification of health economic assumptions (discount rate, currency, analysis time horizon, age-weighting)
4) Appropriate and explicit estimation of disability weights (the importance of this item depends on the extent to which the intervention influences morbidity *vs.* mortality)

An average score of both the reviewers should be entered for the **Consensus copy**.

Factors affecting quality:

Health economic methodology: Any significant errors taken within the health economic methodology. This includes but is not limited to, discounting, perspective, reporting of ratios, data sources, and modeling methodology, as interpreted and considered by the reader/reviewer.

Uncertainty: The reader-perceived existence of any significant errors within the uncertainty analysis, as interpreted and considered by the reader/reviewer.

Transparency: The reader-perceived transparency of the model as conveyed from the manuscript's text and figures, as interpreted and considered by the reader/reviewer.

No/Minor problem- When any minor or no problem exists in the below explained categories

Major problem - When a major problem (s) that could change results or an omission that, through inclusion, would significantly improve the models/study exists   Instances of Major problem should be noted and commented upon within Question #23, Comments.

23. Comments

Any general notes or comments regarding the methods of analysis employed, or information not captured elsewhere in this form.

Ratio Information

A ratio is specified by three characteristics: (i) the target population (Q #1), (ii) the intervention (Q #2), and (iii) the comparator (Q #3). That means no two ratios reported for the same paper should have identical entries for all three of these characteristics. If two values are reported for the same ratio (i.e. the same three elements), the two ratios differ because of an alternative assumption (e.g. the use of a societal perspective, rather than a health care system perspective).

1. **Target Population**

   A. Health status of the target population, coded either as a general health or a specific comorbidity. General health can be stated as healthy (text box), if the study looks at healthy population.

   B. Gender: If the intervention is for a specific gender- male/female/both sex. Not specified: If male or female gender is not specified in the study.

   C. Age: Please note that multiple response are accepted for this variable

   Children, 0-5 years: if the target population includes children between the ages of 0-5 years
   Children, 6-11 years: if the target population includes children between the ages of 6-11 years
   12-18 years: if the target population includes adolescents between the ages of 12-18 years
   19-40: if the target population included those between 19 and 40 years old
   41-64: if the target population included those between 40 and 64 years old
   ≥65: if the target population included those greater than 65 years old
   Adult (not specified): if the target population included non-specific adult (>18 years old) population
   Unknown/Not Specified: Age range not indicated
   Age/Age Range, specify: specify the ages/age range used of the target population.

   Please note that 'pediatric' not otherwise specified should include all children less than or equal to 18 years old

   D. Country/Region: Country of the target population, entered even if this population is the same country as recorded within the article information.

   E. Other characteristics of the target population: The recipients of the primary intervention and comparator. Include other characteristics (e.g. co-morbidities) not included in text box A (Specific disease field in health status).

2. **Intervention** The primary technology, procedure, or health services evaluated in the study.

   Please note that it is essential to provide sufficient detail that the intervention and comparators are clearly described.

3. **Comparator** The alternative to which the intervention is compared. Common comparators are "usual care", "standard of care", placebo, or none (doing nothing).

Comparator_OtherText- Include other active comparators as described in the study

****Readers are encouraged to not make new ratio forms for different dosage strategies, different time-horizons, different perspectives or different discount rates. These alternate scenarios should be noted in Q12 under Uncertainty Analyses.**

4. **$/DALY Averted from article**

The incremental cost-effectiveness ratio (ICER) reported in the original article: $\Delta Costs / \Delta DALYs\ Averted$. This is a numeric field that must be non-negative.

5. **Perspective:**

The perspective of *this particular ratio.* This selection clarifies the identification of ratios for those analyses conducting both societal and health care system perspective analysis.

Health Care Payer: Includes only monetary costs incurred by a (typically 3rd party) health care payer (e.g. National health insurance, Medicare/Medicaid, an HMO or private health care plan).

Health Care Sector: *Health Care Sector* perspective is similar to *Health Care Payer*. However, the *Health Care Sector* perspective accounts for all monetary costs associated with health care, regardless who bears the cost; including costs borne by patients (out-of-pocket costs).

Please note that when considering DALY studies conducted in low and middle income countries, a key distinction between the *Health Care Sector* and *Health Care Payer* perspectives is that the *Health Care Sector* perspective includes out-of-pocket costs and *Health Care Payer* does not.

Limited Societal: *Limited Societal* perspective analysis includes non-healthcare related costs like patient time and productivity that are a direct result of the intervention. The perspective is designated "limited societal" if at least one such cost is included (e.g. unpaid caregiver time, productivity impacts, patient time, patient out of pocket costs).

Societal: The *Societal* perspective is broader than limited societal perspective. It represents the overall public interest by including social opportunity costs where the use of limited resources (such as personnel, hospital beds, donor organs, or budgets) results in the loss of opportunity to use those funds (or resources) for other purposes. This goes beyond the *Limited Societal* perspective in that the analysis accounts for impacts on other sectors, such as environmental, educational, and judicial.

Not Stated/Could Not Be Determined: The authors did not provide sufficient information to determine type of costs or benefits evaluated

Other: Authors report a perspective other than societal or health care payer

6. Was this ratio reported as part of a sequential analysis?

Report if the ICER is part of a sequential analysis (when interventions are arranged sequentially in an ascending order of their increasing DALY averted values and the incremental cost-effectiveness of one intervention is compared to another intervention with lower DALYs sequentially). If it not reported in text but the required information is provided in a table format, then reviewers are asked to create new Ratio forms for the additional ratios.

Ratio Calculation

7. Sample Size

A. Real population size: The number of people **that are eligible for the evaluated intervention**. Note that this is **NOT** the number of subjects (either real or modeled) that were studied or evaluated within the trial. Some studies carry out budget impact analysis or include the entire population of the country/state or city in the analysis, in such cases include the reported population size here. Enter the population value if stated in the stated or estimated by the reviewer. If estimated then enter a brief explanation and source of information. If the value is not stated in the article and cannot be estimated select 'Could not be Determined'.

B. Table of cost and DALYs averted values

Note: Make sure to convert to per patient values for results reporting entire population values.

Per patient values	**Per Person**		
	Intervention	Comparator	Difference
Direct Medical Costs			
Non-Health Care Costs			
Total Costs			
DALYs Averted			
$/DALYs Averted			Auto-Calculated
Entire population	**Entire population**		
	Intervention	Comparator	Difference
Direct Medical Costs			
Non-Health Care Costs			
Total Costs			
DALYs Averted			
$/DALYs Averted			Auto-Calculated

Direct Medical Costs: Health care resource costs related to the intervention. These costs include impacts directly attributable to the intervention and those related to current and future medical consequences to the intervention (e.g. hospitalization, long-term care, other health care services including medications, outpatient procedures and laboratory costs).

Non-Health Care Costs: Non-health care costs resources related to the intervention (e.g. travel time to doctor, caregiver time and workplace productivity impacts, transportation costs, patient productivity costs, implementation costs).

Cost/DALY averted from reader:
Automatically Recalculated value based on the incremental costs and DALYs averted from within the manuscript and identified by the reader.

8. Ratio Quadrant- $/DALY Averted Quadrant

The quadrant describes where the ratio is located in the cost-effectiveness plane (see figure, below).

A ratio lies in QUADRANT I (the northwest quadrant) if the intervention increases costs but does not improve health (fewer DALYs averted). Ratios in QUADRANT I are sometimes referred to as "dominated".
A ratio lies in QUADRANT II (the northeast quadrant) if the intervention increases costs and improves health (more DALYs averted).
A ratio lies in QUADRANT III (the southwest quadrant) if the intervention saves money and does not improve health (fewer DALYs averted).
Finally, a ratio lies in QUADRANT IV (the southeast quadrant) if it both improves health and saves money (more DALYs averted). Ratios in QUADRANT IV are sometimes referred to as "dominant".

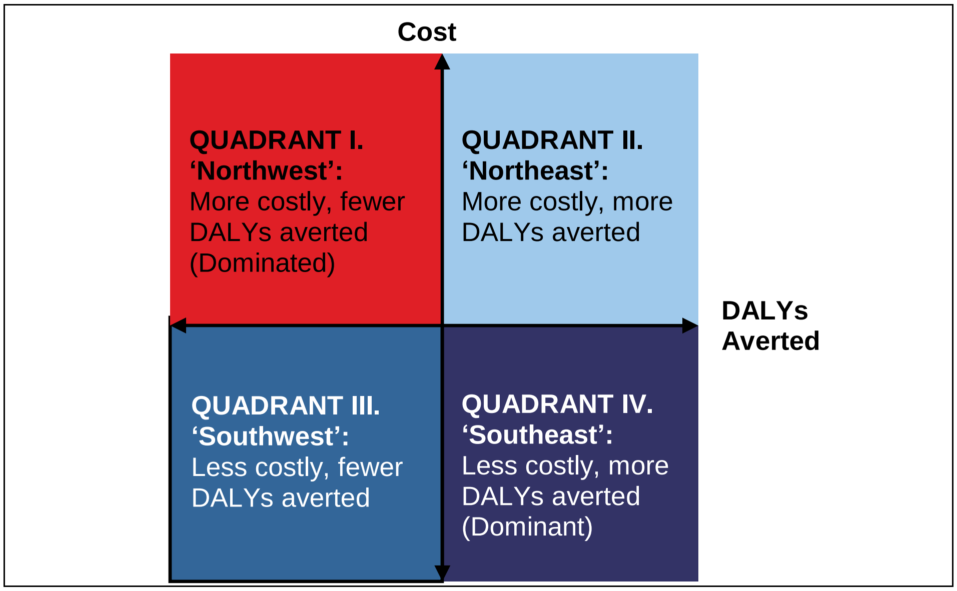

Figure 3: Four Quadrants of Cost-Effectiveness Plane

9. Other CE Ratios Reported

Report other incremental cost-effectiveness ratio measured in other units of effectiveness (e.g. cost per case saved (cost/stroke avoided) or cost per clinical bad outcome avoided.

Cost/QALY from Article: If the study states cost/QALY ratios, please make a note of it.

10. Did the study report a budget impact on a particular payer?

The budget impact is an estimated aggregate cost for the actual (rather than a hypothetical) population. The budget impact can be calculated only if the size of the population eligible for the intervention is known.

BudgetImpactTimeline: If budget impact is stated, then state the timeline provided in the study.

11. Graphic Ratio Uncertainty:

Cost-effectiveness acceptability curve- presented within manuscript (corresponding to this particular ratio).
Scatter plot- presented within manuscript (corresponding to this particular ratio).
Tornado Diagram- a means of presenting multiple univariate sensitivity analyses (corresponding to this particular ratio).
None- The manuscript did not present any graphical representation of the uncertainty surrounding this particular ratio

12. Probabilistic Uncertainty:

Cost-effectiveness acceptability curve: Paper presented probabilistic sensitivity analysis as a cost-effectiveness acceptability curve. Note that the graph need not be presented for this to be true (e.g. 90% of simulations < $50K threshold would qualify).
Confidence interval(s): Paper presented probabilistic sensitivity analysis as a function of a particular confidence interval.
None: The manuscript did not report any probabilistic sensitivity analysis within the graphs or the text

A. Cost-effectiveness acceptability curve:

ACU I_%: presents the percent of simulations/patients meeting a particular threshold identified by ACU I_ICER (iii) below.
ACU I_ICER: Threshold value corresponding to ACU I_%.
ACU II_%: presents the percent of simulations/patients meeting a particular threshold identified by ACU II_ICER (iv) below.
ACU II_ICER: Threshold value corresponding to ACU II_%.
ACU III_%: presents the percent of simulations/patients meeting a particular threshold identified by ACU III_ICER (iv) below.

ACU III_ICER: Threshold value corresponding to ACU III_%.

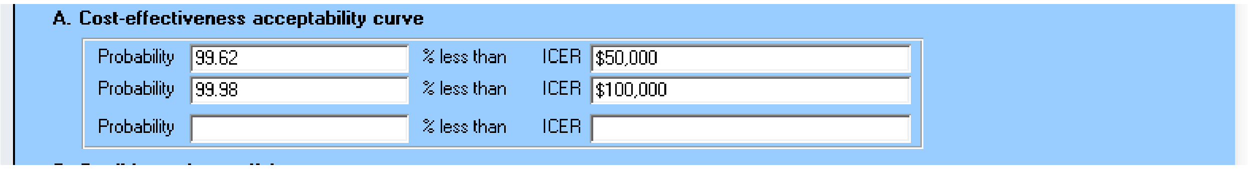

B. Confidence Interval:

CI_ICER: If the manuscript presents a confidence interval of the ratio
CI_ICERText: Range of ICER corresponding to the confidence intervals. Enter lower CI and upper CI separated by "-".
CI_Costs/DALYs Averted: True/False, whether the costs and DALYs confidence intervals are separately reported
CI_Costs/DALYs CostsText: If CI_Cost/DALYs is 'Yes', then the reported confidence interval of the costs.
CI_Costs/DALYs DALYsText: If CI_Cost/DALYs is 'Yes', then the reported confidence interval of the DALYs gained.

13. Input variables causing most uncertainty:

Uncertainty reflects the range of plausible values for the ratio and arises because the true value of one or more parameters or assumptions is unknown. Characterizing a ratio's uncertainty involves specifying this range and noting the primary responsible source of the uncertainty.

Input variables: State the input variables causing most uncertainty in the cost/DALY ratio

Modification: For each influential variable, what type of distribution, range or percent change, increase/decrease used for the input range (e.g. triangle, beta distribution, change in discount rate, costs etc.)

ICER point estimate: ICER point estimate/range corresponding to the input variable and the modification type.

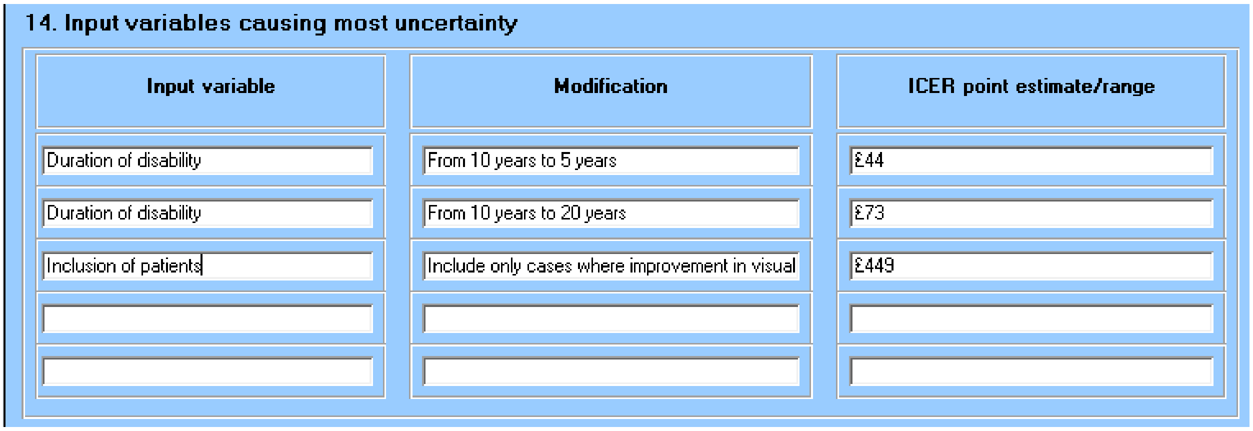

14. Other ratio uncertainty:

Include other important sensitivity analyses that could not be entered in Q13.

15. Comments:

Any comments the reader found noteworthy during the review of the ratio(s).

DISABILITY WEIGHTS INFORMATION

DISABILITY WEIGHT

Disability-adjusted life years (DALYs) are disease-specific and do not pertain to specific to health states. A single disease can encompass multiple health states over time. DALYs have two components that are summed: time lived with disability (YLD) and time lost due to premature mortality (YLL). Because DALYs are disease specific, they do not account for adverse events or comorbid conditions.

DALY = YLL + YLD ; where YLL is the years of life lost due to premature mortality, YLD (years lived with disability) = $I \times DW \times LD$

I is the number of incident cases, DW is the disability weight and LD is the average duration of disability.

As such, the values of Disability Weights are key for determining DALYs. A Disability Weight is a metric for the decline of health associated with a certain heath state, varying between zero (perfect health) and one (death). In regards to the conceptual framework, Disability Weights assume the existence of distinct constructs of health and disability.

Please note that all the information entered on this form is specific and unique to each individual weight. If a study reports multiple weights, then the reference information, patient characteristics, and sample population may vary for each weight. In case of multiple disability weights, make sure to report each weight on a separate form.

1. **Health state considered:**

   The disutility or disability weight for the specific health state/condition. Please be concise and specific regarding the disease the weight is associated with.

2. **Base case disutility or disability:**

   The weight value reported in the base case analysis.

3. **Weight Range for sensitivity analyses:**

   RangeLower: Lower value of weight range
   RangeUpper: Upper value of weight range
   RangeOther: If not reported in numbers, state the range
   WeightRangeOther: If not reported in numbers, state the range here.

4. **Characteristics:**

   A. Gender: Gender of the applicable weight.
   If it is not explicitly mentioned whether the disability weight applies to Male/Female patients then based on the description provided in the study you can select Both.
   Example: for “an adult population with diabetes”, you can use your judgment and select ‘Both’

B. Age
Note: multiple response are accepted for this variable

Children, 0-5 years: if the target population includes children between the ages of 0-5 years
Children, 6-11 years: if the target population includes children between the ages of 6-11 years
12-18 years: if the target population includes adolescents between the ages of 12-18 years
19-40: if the target population included those between 19 and 40 years old
41-64: if the target population included those between 40 and 64 years old
≥65: if the target population included those greater than 65 years old
Adult (not specified): if the target population included non-specific adult (>18 years old) population
Unknown/Not Specified: Age range not indicated
Age/Age Range, specify: specify the ages/age range used of the target population.

Note: 'pediatric' not otherwise specified should include all children less than or equal to 18 years old

5. **Data Source**

Primary data: Data is derived from author's collection of utility weights generated specifically for this study.
Secondary data: Data is derived from another source other than this study.

6. **If secondary data are used where are they from?**

A. The calculation of YLL depends on the assumed "baseline" life expectancy. Life expectancy values in DALY studies can be based on data from various countries or WHO's life tables for WHO member states. YLD depends on the weights used for various diseases. The data source variable will identify the sources of information used for this information.

DALY weight sources include the following options:
Global Burden of Disease (GBD)

- GBD 1990_____(cite reference)
- GBD 2001-2002____(cite reference)
- GBD 2004_____(cite reference)
- GBD 2010_____(cite reference)
- Other________ (cite reference)

Other _______________ (cite references)

B. The data source for life expectancy or mortality is reported in a text field. Please include both the reference number and citation information

7. **Sample population types**

The population queried to elicit utility values. This population may be the same as the study population, or it may be different.

Community: Community means a representative sample of non-patients.

Clinician: Clinician judgment was the basis for the utility weights, or if clinicians were surveyed.

Patient: Patients in the health state in question were queried.

Author: When the authors made an assumption about the disability weight for the disease state.

Relatives/Proxies: If the patient's parent, significant other or child was queried.

Unknown/Not Stated: No information provided.

Other: Other than choices above

8. **Sample Size**: The sample size refers to the utility study sample size, not the population in the Ratio table. This information is frequently not reported for utilities from secondary data.
9. **Comments Notes**: Any comments the reader found noteworthy during the review of the disutility or disability weight(s).
