# Supplementary material for: Comparing the cost-per-QALYs gained and cost-per-DALYs averted literatures
Source: Gates Open Res. 2018 Mar 5;2:5. Originally published 2018 Jan 18. [Version 2] doi: 10.12688/gatesopenres.12786.2 (PMC5801595; doi:10.12688/gatesopenres.12786.2)
Supplement: Peer review history

# Peer review: Referee response for version 1

**DOI:** 10.21956/gatesopenres.13846.r26225

**Published:** 2018-02-05

**Authors:** Rachel Nugent

**Affiliations:** 1 RTI International, Seattle, WA, USA; 2 Department of Global Health, University of Washington, Seattle, WA, USA

The authors have provided a useful summary of the up-to-date contents of the Tufts Medical Center CEA Registry and Global Health CEA Registry, which they manage. In particular, they contrast the contents of the two databases in regard to number of studies, geographic, disease burden, and disease-specific content. This provides a useful - if somewhat simplistic - overview of the availability and contents of current CEA studies. A few comments regarding the results as presented are provided below, along with a few suggestions about additional ways to interrogate the databases.

1. I am interested to see the time series of cost per QALY and cost per DALY studies presented in Figure 1. There is nothing especially surprising here for anyone working in the field, and it is heartening to see the steady increase in economic evaluations for health. I would appreciate the authors highlighting a few aspects of the databases that help one interpret the data. The methods clearly state that the databases draw from English-language articles indexed in PubMed, but it would be worth underscoring that selection creates a downward bias on the true number of cost per DALY studies. It would be very interesting to know if any literature has assessed the change over time of economic evaluations in local-language journals, which would provide an additional signal of the state of economic capacity in LMIC regions.
2. The authors have a rich longitudinal database that could be further analyzed to assess such questions as how changes in the funding sources or disease patterns over time affect the number of cost per QALY or cost per DALY studies. Looking specifically at the cost per DALY numbers over time, how does one understand the growth in study numbers? Is it strongly correlated with growth in global health funding? (I would guess so), and are numbers of disease-specific studies correlated with change in disease burden? (I would guess not). The authors could show rates of growth year by year which would make comparisons across years and types of studies easier.
3. The metric of "under-" and "over-" studied as determined by the DALY burden is also interesting and mostly unsurprising. Perhaps more could be said about the countries and sub-regions that show up green on both maps - such as North Africa, Middle East, and parts of Latin America. Those are the regions truly deficient in economic evaluations. Another point about the literature coverage relative to disease burden is to consider the demographics of the respective Super Regions. Since sub-Saharan Africa and South Asia have younger populations, they also merit more analysis of childhood conditions. If an age-specific disease burden measure were used as the scalar, would the conclusions about "over-" and "under-" studied be the same?
4. Like other reviewers, I find some issue with the statement about "historic proclivities" driving the choice between cost per QALY and cost per DALY, but for a different reason. The methodological underpinnings of the two measures require different types of data, some of which is culturally or contextually determined. Measuring disease prevalence is more straightforward - albeit not simple - than measuring attributes and states of health, and therefore more readily available in countries with limited data capacity; thus creating the means to produce more cost per DALY studies.

I have read this submission. I believe that I have an appropriate level of expertise to confirm that it is of an acceptable scientific standard.

# Response

**Published:** 2018-02-27

**Authors:** Peter Neumann

**Comments from Rachel Nugent** , RTI International, Seattle, WA, USA;  Department of Global Health, University of Washington, Seattle, WA, USA

***Comment #1.  The authors have provided a useful summary of the up-to-date contents of the Tufts Medical Center CEA Registry and Global Health CEA Registry, which they manage. In particular, they contrast the contents of the two databases in regard to number of studies, geographic, disease burden, and disease-specific content. This provides a useful - if somewhat simplistic - overview of the availability and contents of current CEA studies. A few comments regarding the results as presented are provided below, along with a few suggestions about additional ways to interrogate the databases***.

***I am interested to see the time series of cost per QALY and cost per DALY studies presented in Figure 1. There is nothing especially surprising here for anyone working in the field, and it is heartening to see the steady increase in economic evaluations for health. I would appreciate the authors highlighting a few aspects of the databases that help one interpret the data. The methods clearly state that the databases draw from English-language articles indexed in PubMed, but it would be worth underscoring that selection creates a downward bias on the true number of cost per DALY studies. It would be very interesting to know if any literature has assessed the change over time of economic evaluations in local-language journals, which would provide an additional signal of the state of economic capacity in LMIC regions***.

Response:

We have added the following text to a new limitations section of the Discussion:

*… the databases we used are restricted to English-language articles indexed in PubMed.  This restriction may have depressed the number of cost-per-DALY studies we identified to a greater extent proportionally than it may have depressed the number of cost-per-QALY studies we identified because a smaller proportion of the cost-per-DALY literature focuses on English-speaking countries.*

***Comment #2.  The authors have a rich longitudinal database that could be further analyzed to assess such questions as how changes in the funding sources or disease patterns over time affect the number of cost per QALY or cost per DALY studies. Looking specifically at the cost per DALY numbers over time, how does one understand the growth in study numbers? Is it strongly correlated with growth in global health funding? (I would guess so), and are numbers of disease-specific studies correlated with change in disease burden? (I would guess not). The authors could show rates of growth year by year which would make comparisons across years and types of studies easier.***

Response:

These are interesting questions, although we believe they go beyond the scope of what we set out to address.  We have added text to the Discussion section of the paper to note areas for future research, including trends in the CEA literature in terms of diseases and geographic regions covered, funding patterns among donor organizations, and whether published studies correspond to society’s most pressing needs.

***Comment #3.  The metric of "under-" and "over-" studied as determined by the DALY burden is also interesting and mostly unsurprising. Perhaps more could be said about the countries and sub-regions that show up green on both maps - such as North Africa, Middle East, and parts of Latin America. Those are the regions truly deficient in economic evaluations. Another point about the literature coverage relative to disease burden is to consider the demographics of the respective Super Regions. Since sub-Saharan Africa and South Asia have younger populations, they also merit more analysis of childhood conditions. If an age-specific disease burden measure were used as the scalar, would the conclusions about "over-" and "under-" studied be the same?***

Response:

These points are likewise interesting.  We defer to future researchers to organize the data as needed and conduct these analyses.

***Comment 4.  Like other reviewers, I find some issue with the statement about "historic proclivities" driving the choice between cost per QALY and cost per DALY, but for a different reason. The methodological underpinnings of the two measures require different types of data, some of which is culturally or contextually determined. readily available in countries with limited data capacity; thus creating the means to produce more cost per DALY studies.***

Response:

We have added text offering further explanation for the discrepancy between use of QALYs and DALYs by country wealth level (see response to Comment #1 from Michael Drummond).

# Peer review: Referee response for version 1

**DOI:** 10.21956/gatesopenres.13846.r26223

**Published:** 2018-02-01

**Authors:** Kalipso Chalkidou, Alec Morton

**Affiliations:** 1 Center for Global Development, London, UK; 2 Department of Management Science, University of Strathclyde, Glasgow, UK

Thank you for the chance to review this paper. 

- This is a useful and timely study presenting informative analyses of the cost-per-QALY gained and cost-per-DALY averted literatures, relating them to geographical regions and to diseases and conditions prominent in these regions, as well as to wealth levels, types of intervention assessed and funding source. The authors draw conclusions as to the respective literatures’ evolution over the years and to things such as how well these link to disease burdens in their respective geographies.
- We would challenge the classification of pharmaceuticals as “tertiary prevention/treatment”. According to WHO, pharmaceuticals make up the bulk of OOP spending in most LICs (~77% based on the 2011 World Medicines Situation) and given fees, access and availability of facilities, self-medication is a major component of healthcare systems in LICs and LMICs.
- We were surprised almost half of the DALY studies have received government funding. Is it possible to tell whether this is national governments of LICs and LMICs or donor governments? Given DALYs are mostly used in LICS and LMICs, are the governments of these countries commissioning this work? According to another study which we believe is worth citing ^1^, BMGF seems to be the single most commonly cited funder of DALY studies in LMICs. Our analysis as part of this paper (unpublished data) found that LIC government funded studies in malaria, TB and HIV studies (using DALYs (mostly) as an outcome measure), made up only 13%, 5% and 7% of the total in each disease area, respectively. Perhaps a more nuanced (eg broken down by decision maker global and domestic) analysis of funding source may reveal important messages assuming the data are available? Such a study would supplement nicely the PLoS paper cited earlier.
- Though probably not for this paper, perhaps a discussion as to why the discrepancy between QALYs and DALYs by wealth level and what the message may be for transitioning countries, is warranted. So the database could be expanded perhaps in the future to include data on the country of origin of authors, which would in turn allow capturing a likely (but unproven) trend from poorer countries where publications come from mostly western authors funded by foreign donor foundations or governments, focus on MDGs and using DALYs to MICs/HICs where local authors funded by local money dominate, and with the focus shifting to NCDS and the use of QALYs. Such an analysis, if it confirms our hypothesis (and there are plenty of anecdotes from countries like the Philippines, China, Thailand, Brazil, Mexico and South Africa where national reference cases use QALYs as the outcome measure of preference), could then help reflect on what this might mean for countries in transition and the type of data and capacity they need to support their transition.
- Figure 1 is useful, but it would be helpful to show the time trends not just in the counts of the papers but in the composition of papers by GBD category and super-region, as well as intervention. A more developed Figure 1 would set up nicely a discussion of what the future might hold and which we touch on earlier in our discussion regarding transition.
- One of us (AM) has downloaded the cost/QALY data set to take a look and found that for 5895 out of 6438 records the field for publication year is blank. This info is needed to generate Figure 1 and so it should be there. It considerably lowers confidence in the integrity of the analysis when one discovers these things within a few seconds of downloading the database. 
- Figure 4 is also very useful but why not show similar analysis for the other GBD regions? Why so few regions? In an increasingly multipolar world it seems highly appropriate to conclude that research priorities in each region should be different. If one could generate graphs for all the GBD regions that would strengthen that case and give a sense of the extent of global diversity.
- The authors write: “The contrast seems to reflect the historical proclivities rather than any inherent advantages for one metric’s use for a particular category of countries” – what might such “inherent advantages” be or indeed the historical proclivities (might the funding source have a role to play given the preference by BMGF a major funder of this work, for DALYs - https://beta.nice.org.uk/Media/Default/About/what-we-do/NICE-International/projects/MEEP-report.pdf)? Why not standardise on the QALY as both the methodological (see Airoldi and Morton ^2^) and empirical foundations of the method are more well-established and there is evidence that when domestic payers make investment decisions in HICs and UMICs, QALY is their preferred outcome?
- The findings relating to under-and over-studied conditions seem to us to be very interesting and relevant (perhaps more so than the QALY/DALY debate). Could the paper be retitled and/or the abstract rewritten to give these findings more prominence?

We have read this submission. We believe that we have an appropriate level of expertise to confirm that it is of an acceptable scientific standard.

## References

[1] SantatiwongchaiBChantarastapornchitVWilkinsonTThiboonboonKRattanavipapongWWalkerDGChalkidouKTeerawattananonY: Methodological variation in economic evaluations conducted in low- and middle-income countries: information for reference case development. *PLoS One*.2015;10(5) : 10.1371/journal.pone.0123853 e0123853 10.1371/journal.pone.0123853 25950443PMC4423853

[2] AiroldiMMortonA: Adjusting life for quality or disability: stylistic difference or substantial dispute?. *Health Econ*.2009;18(11) : 10.1002/hec.1424 1237-47 10.1002/hec.1424 19097040

# Response

**Published:** 2018-02-27

**Authors:** Peter Neumann

**Comments from Kalipso Chalkidou** , Center for Global Development, London, UK

**Alec Morton** , Department of Management Science, University of Strathclyde, Glasgow, UK

**Approved**

***Comment #1.  This is a useful and timely study presenting informative analyses of the cost-per-QALY gained and cost-per-DALY averted literatures, relating them to geographical regions and to diseases and conditions prominent in these regions, as well as to wealth levels, types of intervention assessed and funding source. The authors draw conclusions as to the respective literatures’ evolution over the years and to things such as how well these link to disease burdens in their respective geographies***.

Response:

No response needed.

***Comment #2.  We would challenge the classification of pharmaceuticals as “tertiary prevention/treatment”. According to WHO, pharmaceuticals make up the bulk of OOP spending in most LICs (~77% based on the 2011 World Medicines Situation) and given fees, access and availability of facilities, self-medication is a major component of healthcare systems in LICs and LMICs***.

Response: 

Note that we make no assumptions about an intervention’s prevention stage based on its type. For example, we do not assume that pharmaceuticals are tertiary treatments.  Instead, we assign the prevention level based on how the article describes the disease and the treatment. 

While we had intended the original text to provide examples of typical primary and tertiary treatments, we see that the presentation of the results may have been confusing.  We have therefore eliminated those examples and just report the overall proportion of articles in two categories. The text now reads:

*Tertiary prevention (treatment) dominates the cost-per-QALY registry (62%), whereas the cost-per-DALY registry focuses far more on primary prevention (59%).*

***Comment #3a.  We were surprised almost half of the DALY studies have received government funding. Is it possible to tell whether this is national governments of LICs and LMICs or donor governments? Given DALYs are mostly used in LICS and LMICs, are the governments of these countries commissioning this work? According to another study which we believe is worth citing1, BMGF seems to be the single most commonly cited funder of DALY studies in LMICs. Our analysis as part of this paper (unpublished data) found that LIC government funded studies in malaria, TB and HIV studies (using DALYs (mostly) as an outcome measure), made up only 13%, 5% and 7% of the total in each disease area, respectively. Perhaps a more nuanced (eg broken down by decision maker global and domestic) analysis of funding source may reveal important messages assuming the data are available? Such a study would supplement nicely the PLoS paper cited earlier.***

Response:

We do not have the information needed to assess whether the governments of these countries are commissioning the work. The final paragraph of the Discussion now cites both papers identified by the reviewer and notes the need for further research on this and on other issues.

***Comment #3b.  Though probably not for this paper, perhaps a discussion as to why the discrepancy between QALYs and DALYs by wealth level and what the message may be for transitioning countries, is warranted. So the database could be expanded perhaps in the future to include data on the country of origin of authors, which would in turn allow capturing a likely (but unproven) trend from poorer countries where publications come from mostly western authors funded by foreign donor foundations or governments, focus on MDGs and using DALYs to MICs/HICs where local authors funded by local money dominate, and with the focus shifting to NCDS and the use of QALYs. Such an analysis, if it confirms our hypothesis (and there are plenty of anecdotes from countries like the Philippines, China, Thailand, Brazil, Mexico and South Africa where national reference cases use QALYs as the outcome measure of preference), could then help reflect on what this might mean for countries in transition and the type of data and capacity they need to support their transition.***

Response:

We have added text offering further explanation for the discrepancy between use of QALYs and DALYs by country wealth level (see response to Comment #1 from Michael Drummond) and on the need for further research in this area. 

***Comment #4.  Figure 1 is useful, but it would be helpful to show the time trends not just in the counts of the papers but in the composition of papers by GBD category and super-region, as well as intervention. A more developed Figure 1 would set up nicely a discussion of what the future might hold and which we touch on earlier in our discussion regarding transition.***

******

Response:

We appreciate that providing time trends for other study characteristics, including GBD and super-region would be useful and could provide insight regarding the direction of the literature. In the revised paper, we have noted that as an area for future research and believe that as the cost-per-DALY literature in particular increases in size, the inferences that can be drawn will increase.

***Comment #5.  One of us (AM) has downloaded the cost/QALY data set to take a look and found that for 5895 out of 6438 records the field for publication year is blank. This info is needed to generate Figure 1 and so it should be there. It considerably lowers confidence in the integrity of the analysis when one discovers these things within a few seconds of downloading the database***. 

Response:

We very much appreciate the reviewers pointing out errors in our data extract.  In response, we have regenerated the data extract, this time doing so by implementing all steps in a computer program to reduce the risk of introducing errors through manual manipulation of the original data. We are posting the computer program (written in STATA) and the extracted data.  We have checked the distributions of the extracted data to make sure they appear to be reasonable. 

Note that because we used the original dataset for our statistical analysis in verstion #1 of this paper, the errors in the extract did not affect the results.

***Comment #6.  Figure 4 is also very useful but why not show similar analysis for the other GBD regions? Why so few regions? In an increasingly multipolar world it seems highly appropriate to conclude that research priorities in each region should be different. If one could generate graphs for all the GBD regions that would strengthen that case and give a sense of the extent of global diversity***.

Response:

We have chosen to include figures for only the three regions with the largest number of studies. But to address the reviewer’s comment, we have alsoadded a table that reports the standardized residual for each disease in each super region, relative to the regression line.  We also report the mean and median residual for each disease (across all seven super regions) to characterize which diseases tend to be over- and under-studied in general.

***Comment #7.  The authors write: “The contrast seems to reflect the historical proclivities rather than any inherent advantages for one metric’s use for a particular category of countries” – what might such “inherent advantages” be or indeed the historical proclivities (might the funding source have a role to play given the preference by BMGF a major funder of this work, for DALYs - https://beta.nice.org.uk/Media/Default/About/what-we-do/NICE-International/projects/MEEP-report.pdf)? Why not standardise on the QALY as both the methodological (see Airoldi and Morton2) and empirical foundations of the method are more well-established and there is evidence that when domestic payers make investment decisions in HICs and UMICs, QALY is their preferred outcome?***

Response:

Making recommendations as to what measure the field should use is beyond the scope of this paper. We do, however, provide expanded text in an effort to explain why these measures are each used, and why the QALY measure is used more in high-income countries, and the DALY measure more in lower- and middle-income countries. See response to Comment #1 from Michael Drummond.

***Comment #8.  The findings relating to under-and over-studied conditions seem to us to be very interesting and relevant (perhaps more so than the QALY/DALY debate). Could the paper be retitled and/or the abstract rewritten to give these findings more prominence?***

Response:

As this paper is the first comprehensive effort to describe the cost-per-DALY literature and compare it to the cost-per-QALY literature, we prefer to stick with emphasizing this aspect of the work in the title.

# Peer review: Referee response for version 1

**DOI:** 10.21956/gatesopenres.13846.r26221

**Published:** 2018-01-29

**Authors:** Michael Drummond

**Affiliations:** 1 Centre for Health Economics, University of York, York, UK

Neumann et al. examine the literature on economic evaluation through 2016, focusing specifically on studies measuring the health benefits in quality-adjusted life-years (QALYs) and disability-adjusted life-years (DALYs). A number of the findings of their research are as expected. First, cost per QALY studies have tended to focus of upper-middle and high-income countries, whereas cost per DALY studies have tended to focus on low and lower-middle income countries. This is likely to reflect the greater availability of preference values for health states in higher income countries and the preference of international donors, such as WHO and the World Bank, for studies estimating DALYs in lower income countries. Secondly, while the literature in both cost per QALY and cost per DALY studies is growing over time, there are more than 10 times the number of studies using QALYs than those using DALYs. This is likely to reflect the higher number of economist researchers and greater availability of funding for studies in high-income countries.

 However, another finding of the research is not so easily explained. While the focus on topics for research, tertiary prevention (treatment) for studies using QALYs and primary prevention for studies using DALYs, it is surprising that the literature coverage is not closely aligned to disease burden in either high income or low income countries. Neumann et al. suggest that ‘the most commonly studied diseases, regions and interventions may reflect the financial interests of the CEA funders’. One can see why this might be the case in higher income countries, where many studies are funded by pharmaceutical countries, but it’s not clear why international donors might be favouring some diseases over others in lower income countries. 

 The analysis by Neumann et al. cannot directly answer that question, but one important factor driving economic evaluation in all countries is the number of promising interventions or programmes to evaluate. In this sense, the literature on economic evaluation mostly follows the priorities for research of technology manufacturers or public health specialists. For example, in recent years the research priorities of pharmaceutical companies in higher income countries have focused on specialty drugs for diseases such as cancer. This could be driven by discoveries in basic research or the pursuit of profits, or both. However, in all countries one might expect priorities for research to be driven not by the absolute level of disease burden, but the potential for *modifying* that burden through the development and implementation of health care treatments and programmes.

 One final issue touched on in the paper by Neumann et al. concerns the analytic choice between QALYs and DALYs in conducting economic evaluations. In commenting on the contrast in approach between higher and lower income countries, the authors state that ‘this contrast seems to reflect the historic proclivities of health economist researchers, rather than any inherent advantages for one metric’s use for a particular category of countries’. In my view this issue deserves much deeper investigation.

 In many lower income countries, health economist researchers may not have a realistic choice of approach, as QALYs may not exist for the country concerned. But which approach should the analyst use in a country for which both QALYs and DALYs are available? Comparisons between QALYs and DALYs and the implications for health policy decisions have been discussed in the papers by Airoldi and Morton (2009) ^1^ and Robberstad (2005) ^2^, with the conclusion that different decisions might be reached.

 Although there are some minor differences in the theorectical constructs of QALYs and DALYs, two practical issues may be critical to the choice of approach. On the one hand QALYs are likely to be more ‘bespoke’ to the country where the study is being conducted and are more likely to reflect the health state preferences in the country concerned. However, on the other hand there is considerable variability in the methods used to elicit the preferences for health states in QALYs, which may threaten any standardized approach to decision-making. This issue has been recognized by the National Institute for Health and Care Excellence (NICE) in the United Kingdom, which, while recommending the use of QALYs, specifies the characteristics of the instrument that should be used to estimate them ( NICE, 2013). By an extension of the same argument, an international donor requiring some standardization of approach to evaluation across several countries is likely to recommend the use of DALYs.

 I answered 'Partly' to the question "Are sufficient details of methods and analysis provided to allow replication by others?" as access to the databases would be required for full replication.

I have read this submission. I believe that I have an appropriate level of expertise to confirm that it is of an acceptable scientific standard.

## References

[1] AiroldiMMortonA: Adjusting life for quality or disability: stylistic difference or substantial dispute?. *Health Econ*.2009;18(11) : 10.1002/hec.1424 1237-47 10.1002/hec.1424 19097040

[2] RobberstadB: QALYs vs DALYs vs LYs gained: What are the differences, and what difference do they make for health care priority setting?. *Norsk Epidemiologi*.2009;15(2) : 10.5324/nje.v15i2.217 10.5324/nje.v15i2.217

# Response

**Published:** 2018-02-27

**Authors:** Peter Neumann

**Comments from Michael Drummond** , Centre for Health Economics, University of York, York, UK

**Approved**

***Comment #1.  Neumann et al. examine the literature on economic evaluation through 2016, focusing specifically on studies measuring the health benefits in quality-adjusted life-years (QALYs) and disability-adjusted life-years (DALYs). A number of the findings of their research are as expected. First, cost per QALY studies have tended to focus of upper-middle and high-income countries, whereas cost per DALY studies have tended to focus on low and lower-middle income countries. This is likely to reflect the greater availability of preference values for health states in higher income countries and the preference of international donors, such as WHO and the World Bank, for studies estimating DALYs in lower income countries. Secondly, while the literature in both cost per QALY and cost per DALY studies is growing over time, there are more than 10 times the number of studies using QALYs than those using DALYs. This is likely to reflect the higher number of economist researchers and greater availability of funding for studies in high-income countries*** **.**

Response:

We agree with the reviewer comments and have revised the Discussion to incorporate these points. We have added the following text to the Discussion:

*Several factors may explain why cost-per-QALY studies predominate in high-income countries, while cost-per-DALY studies are more popular in lower and middle-income countries.  The differences could, for example, reflect the availability of health utility weights in high-income countries and the lack of such information in lower-income settings.  Researchers conducting CEAs in countries with limited data capacity may find it easier and less expensive to use the cost-per-DALY metric.*

*The differences could also reflect the preferences and traditions of organizations that fund CEA studies.  Foundations funding global health research may prefer the DALY metric, given the historic use of DALYs to measure global disease burden.  In contrast, health authorities in high-income countries (e.g., the National Institute for Health and Care Excellence (NICE) in the United Kingdom) have tended to recommend the use of QALYs in CEAs.  The geographic differences between the cost-per-QALY and cost-per-DALY literature deserve further investigation, as our effort did not gather information on why authors used these measures.*

***Comment #2.However, another finding of the research is not so easily explained. While the focus on topics for research, tertiary prevention (treatment) for studies using QALYs and primary prevention for studies using DALYs, it is surprising that the literature coverage is not closely aligned to disease burden in either high income or low income countries. Neumann et al. suggest that ‘the most commonly studied diseases, regions and interventions may reflect the financial interests of the CEA funders’. One can see why this might be the case in higher income countries, where many studies are funded by pharmaceutical countries, but it’s not clear why international donors might be favouring some diseases over others in lower income countries***. 

Response:

We agree with the reviewer and have added the following paragraph to the Discussion:

*There is no clear explanation for these inconsistencies.  As we have noted elsewhere, decisions to fund or conduct economic evaluations reflect not just the disease burden imposed by the targeted condition, but also the number of promising interventions or programs 19, 20. Because specialty drugs for diseases such as cancer represent important new interventions in high-income countries, and because pharmaceutical companies have the resources and incentive to characterize value for those interventions, much of the cost-per-QALY literature has recently focused on specialty drug therapies.  These financial incentives are less pronounced in the lower- and middle-income countries that are much more the focus of the cost-per-DALY literature.  In addition to disease burden, priorities in the cost-per-DALY literature may reflect the visibility and emotional salience of diseases, the influence of advocacy groups, the vagaries of reimbursement decisions ^19^, and institutional priorities of the organizations sponsoring the research.*

***Comment #3.  The analysis by Neumann et al. cannot directly answer that question, but one important factor driving economic evaluation in all countries is the number of promising interventions or programmes to evaluate. In this sense, the literature on economic evaluation mostly follows the priorities for research of technology manufacturers or public health specialists. For example, in recent years the research priorities of pharmaceutical companies in higher income countries have focused on specialty drugs for diseases such as cancer. This could be driven by discoveries in basic research or the pursuit of profits, or both. However, in all countries one might expect priorities for research to be driven not by the absolute level of disease burden, but the potential for modifying that burden through the development and implementation of health care treatments and programmes***.

Response:

See response to Comment #3.

***Comment #4.  One final issue touched on in the paper by Neumann et al. concerns the analytic choice between QALYs and DALYs in conducting economic evaluations. In commenting on the contrast in approach between higher and lower income countries, the authors state that ‘this contrast seems to reflect the historic proclivities of health economist researchers, rather than any inherent advantages for one metric’s use for a particular category of countries’. In my view this issue deserves much deeper investigation.***

******

***In many lower income countries, health economist researchers may not have a realistic choice of approach, as QALYs may not exist for the country concerned. But which approach should the analyst use in a country for which both QALYs and DALYs are available? Comparisons between QALYs and DALYs and the implications for health policy decisions have been discussed in the papers by Airoldi and Morton (2009)*** ***^1^*** ***and Robberstad (2005)*** ***^2^*** ***, with the conclusion that different decisions might be reached.***

******

***Although there are some minor differences in the theorectical constructs of QALYs and DALYs, two practical issues may be critical to the choice of approach. On the one hand QALYs are likely to be more ‘bespoke’ to the country where the study is being conducted and are more likely to reflect the health state preferences in the country concerned. However, on the other hand there is considerable variability in the methods used to elicit the preferences for health states in QALYs, which may threaten any standardized approach to decision-making. This issue has been recognized by the National Institute for Health and Care Excellence (NICE) in the United Kingdom, which, while recommending the use of QALYs, specifies the characteristics of the instrument that should be used to estimate them (*** ***NICE, 2013*** ***). By an extension of the same argument, an international donor requiring some standardization of approach to evaluation across several countries is likely to recommend the use of DALYs***.

Response:

See response to Comment #1.

***Comment #5.  I answered 'Partly' to the question "Are sufficient details of methods and analysis provided to allow replication by others?" as access to the databases would be required for full replication***.

Response:

We have made all data used in the analysis available, along with the computer code used for analyses and to create tables and figures.
